# A Self-Adaptive Multi-Learning Strategy Particle Swarm Optimizer for UWB Indoor Positioning Anchors Layout Optimization

**DOI:** 10.3390/s26175535

**Published:** 2026-08-31

**Authors:** Xing Zhou, Zhenyu Li, Liyang Wang, Gongwei Xiao

**Affiliations:** 1 Academy of Surveying and Mapping Engineering of Gansu Province, Lanzhou 730000, China; gscors@163.com (X.Z.); walkmanwangl@163.com (L.W.); 2 BDS Applications and Survey Datum Service Gansu Sub-Center, Lanzhou 730000, China; 3 Emergency Mapping Engineering Research Center of Gansu, Lanzhou 730000, China; 4 Gansu Provincial Ecological Restoration Monitoring and Evaluation Technology Innovation Center, Lanzhou 730000, China; 5 School of Communications and Information Engineering, Xi’an University of Posts and Telecommunications, Xi’an 710121, China; xiaogongwei@xupt.edu.cn

**Keywords:** PSO, self-adaptive selection, social learning, comprehensive learning, generalized opposition-based learning, dimensional learning, UWB anchors layout

## Abstract

Particle swarm optimization (PSO) is a widely used method for solving single-objective optimization problems. However, PSO suffers from diversity loss and premature convergence, leading to suboptimal performance in complex problems. To address these limitations, this paper introduces a novel self-adaptive multi-learning strategy PSO (SMLS-PSO) designed for real-parameter optimization tasks. SMLS-PSO integrates four distinct learning strategy-based PSO variants into a behavior pool and employs a new self-adaptive strategy selection mechanism. This mechanism dynamically chooses the most suitable learning strategy to update the velocities and positions of particles based on fitness information and payoff at different evolutionary stages. To enhance the performance of SMLS-PSO, three key improvements are incorporated: (1) a stagnation counter parameter to minimize wasted fitness evaluations; (2) a boundary symmetry mapping method to manage out-of-range searches; and (3) a Quasi-Newton local search operator to boost local exploitation capabilities. SMLS-PSO is first compared with four component PSO variants on 13 basic benchmark functions. Subsequently, SMLS-PSO is compared with eight state-of-the-art PSO variants on both 30D and 50D CEC2017 test suite problems. Experimental results indicate that SMLS-PSO statistically and significantly outperforms the compared algorithms on the majority of the test problems, showcasing its superior optimization capabilities. Finally, SMLS-PSO was applied to the optimization problem of UWB anchors layout, and it achieved a higher locatable space coverage rate and a better average HDOP value compared to the conventional layout scheme and other PSO variant optimization schemes.

## 1. Introduction

PSO is an Evolutionary Algorithm (EA) that was primarily designed by Kennedy and Eberhart in 1995, particularly to address practical parameter optimization issues [1]. The PSO algorithm has attracted widespread interest in many fields, including engineering design issues [2,3], satellite navigation [4,5], control systems [6], and resource allocation [7]. Despite its apparent simplicity, PSO has been shown to be competitive with more complex optimization algorithms. However, numerous studies have shown that when dealing with issues involving complex fitness landscapes, the search effectiveness of various PSO variants differs noticeably [8]. In other words, a particular PSO version could be effective in some situations while being ineffective in others. An algorithm may also perform differently at different stages of evolution, even for the same task. Finding the proper PSO versions for various situations at various stages is crucial. In the last two decades, numerous modified versions of PSO have emerged with the aim of improving its effectiveness. These PSO variants can be broadly classified into four categories: population neighborhood topology and multi-swarm techniques, parameter control, hybridization with other algorithms, and novel learning strategies.

This paper will not go into great detail on the first three sections. The learning strategy-based PSO (LPSO) is an extension of the traditional PSO. It incorporates learning operators or specific learning mechanisms. This integration endows the algorithm with a certain learning capability. As a result, it promotes more frequent communication within the population. This integration facilitates information sharing among individuals, thereby accelerating convergence speed and improving the precision of locating the optimal solution [9]. A considerable amount of research has been performed on LPSO by numerous researchers. The comprehensive learning PSO (CLPSO) [10] modifies individuals’ speed by learning the most accurate history data of all other particles. By avoiding early convergence, this learning technique maintains population diversity. With the orthogonal learning PSO (OLPSO) [11], all particles can acquire more practical knowledge by combining the *pbest* and *gbest* to create a learning example for each particle. The particle search could be efficiently guided and enhanced by the OL approach. To avoid the issue of the population becoming trapped in local optima, Wang et al. [12] proposed an enhanced version of PSO which utilizes a combination of Cauchy mutation and generalized opposition-based learning strategies, referred to as GOPSO. In the Social Learning PSO (SL-PSO) [13], particles are permitted to draw knowledge from any superior particles, known as demonstrators, within the existing swarm. A unique Genetic Learning PSO (GL-PSO) [14], which consists of two cascading layers, was proposed to enhance global search capabilities. It is the first to use the selection, mutation, and crossover operators of a genetic algorithm to generate a promising learning exemplar. The second stage updates the swarm’s position and velocity using the canonical PSO algorithm. The second layer uses the standard PSO algorithm to update the swarm’s position and velocity. DLPSO [15] employs a dimensional learning strategy (DLS) to generate learning exemplars. Specifically, for each dimension of a particle’s *pbest*, it learns from the corresponding dimension of the *gbest*. Beyond the previously mentioned learning approaches, the evolution of various other learning mechanisms is continuously progressing. These include Bayesian networks learning, cooperative learning, learning automata, Q-learning, and reinforcement learning.

Since many PSO variations only employ one search method to update all particle positions, the population may lack diversity and be unable to flexibly select various velocity and position updating strategies under various fitness circumstances for various issues. However, these various PSO variations can complement one another when combined, which enables us to handle a variety of optimization issues [8]. This approach is also known as an ensemble approach because every particle will use two or more methods to update its position. Additionally, the ensemble approach in EA has become a popular topic of study [16]. The key differences between ensemble techniques are in the control mechanisms for the dynamic adjustment strategy and the suitable updating strategy.

Du and Li [17] introduced a multi-strategy ensemble PSO (MEPSO) designed for dynamic optimization. In this approach, the particles are categorized into two groups. Group I incorporates a Gaussian local search strategy. The purpose of this is to improve the convergence capability. Meanwhile, Group II utilizes a differential mutation strategy. This strategy serves to broaden the search space and boost the divergent performance of the PSO algorithm. Consequently, it is better equipped to handle problems across diverse dynamic environments. Engelbrecht created the heterogeneous PSO (HPSO) method [18], which effectively addresses the exploration–exploitation trade-off problem by allowing particles to adopt various search behaviors chosen from a behavior pool. Following the concept of HPSO, Nepomuceno put forward the frequency-based heterogeneous PSO (*f*_k_-HPSO) [19]. This version utilizes different PSO variants to construct a behavior pool. It monitors the success rate of each behavior across multiple iterations. This data is then leveraged to determine the subsequent behavior of a particle. On the basis of CLS, HCLPSO [20] split up the swarm into two subpopulations: the exploration subpopulation and the exploitation subpopulation, both of which use a CL strategy. The exploitation subpopulation’s velocities were updated using a global version of the CLPSO strategy, while the exploration subpopulation’s velocities were updated using the CLPSO strategy. Wang et al. suggested a HCLDMS-PSO algorithm, in which CLPSO [10] and DMS-PSO [21] algorithms are chosen to construct a behavior pool. To primarily create an exploitation subpopulation exemplar, the CLPSO algorithm is used with the *gbest* of the whole population. The task of creating an exploration subpopulation is given to the upgraded DMS-PSO algorithm. In the two-swarm learning PSO algorithm (TSLPSO) [15], the exploration subpopulation builds learning exemplars using CLS to direct the global search while the exploitation subpopulation uses DLS to guide the local search of the particles.

The aforementioned ensemble PSO variants can be thought of as static ensemble approaches, meaning that throughout the evolutionary process, the probability or proportion of options remains constant. Variants of the dynamic multi-strategy ensemble PSO exist as well. Their main concept is the dynamic control of the adoption probability of each search strategy. Dynamic heterogeneous particle swarm optimization (dHPSO) [18], which randomly chooses a new strategy from the strategy pool (including PSO, cognitive-only PSO, social-only PSO, barebones PSO, and modified barebones PSO). The drawback of dHPSO is that it lacks a selection mechanism. As a result, the best strategy and worst approach are equally likely to be chosen from the strategy pool. Four PSO-based updating strategies—namely, CLPSO, PSO-CL-pbest, DbV, and EbV—were applied by a dynamic method in the self-adaptive learning-based PSO (SLPSO) [22]. A probability model was employed to indicate the likelihood of each of the four strategies being chosen. The model is self-adaptively enhanced in accordance with the strategies’ propensity to produce earlier generations of higher-caliber solutions. The self-learning PSO (SLPSO) algorithm was also put forth by Li et al. [23]. In SLPSO, each particle can independently handle various situations by adopting four different learning strategies based on an adaptive learning framework, i.e., learning from (1) its *pbest* position, (2) a randomly nearby position, (3) *pbest* position of a random particle, and (4) *gbest* position of the entire population by selecting the most effective strategy in accordance with its own local fitness landscape. In EPSO [8], five PSO strategies were combined, and a dynamic approach was used to update each strategy’s adoption probability. Based on the success and failure recollections of five strategies, EPSO updates the probability that a strategy will be adopted. The likelihood that a strategy will be chosen increases with the success rate.

In order to handle large-scale complex optimization issues, Wang et al. [24] recently introduced the multiple-strategy learning PSO method (MSL-PSO). Particles are changed twice during each generation using various learning techniques. The first stage is utilized to increase exploratory potential, and the second is supposed to balance population variety and convergence. Multi-population ensemble particle swarm optimizer (MPEPSO) [2] adaptively uses three existing efficient and simple PSO updating strategies by a dynamic approach. The particles are categorized into three indicator sub-swarms and a single reward sub-swarm. In each learning phase, the reward subpopulation is dynamically assigned to the most effective strategy. In strategy dynamics particle swarm optimizer (SDPSO) [25], four PSO variants (LDWPSO, UPSO, LIPS, and CLPSO) are used as search strategies because they have different features for solving different problems, and combining these different search strategies may improve the universality or robustness of the PSO algorithm. Simultaneously, evolutionary game theory is introduced to self-adaptively and dynamically control the adoption probability of each strategy.

Ultra-Wide Band (UWB) positioning accuracy is superior at the cm level and is utilized extensively in industrial processing, monitoring, tracking, and other applications thanks to its exceptionally high time resolution, huge bandwidth, and low power consumption [26,27]. The UWB positioning system obtains the estimated position of a mobile tag by measuring range and angle information between multiple anchor nodes with known coordinates and the tag. UWB positioning accuracy is determined by four core factors: time synchronization/ranging error [28], anchor nodes position error, non-line-of-sight (NLOS) propagation error [29], and anchor nodes layout configuration [30]. The first two errors are relatively static, while NLOS propagation is the dominant factor affecting UWB tag positioning performance. The layout of the UWB anchors has a significant impact on the positioning system’s construction costs, accuracy, and stability. It has been demonstrated that the influence of anchor layout on location accuracy is greater than measurement noise and the fast fading effect [28,31].

The use of several classical deployments, such as rectangular and diamond layouts, will lead to an insufficient locatable space coverage range with large blind positioning zones and a significant increase in positioning errors. If the UWB anchors layout is too dense, although it can improve the positioning accuracy, it will also cause cost increases and be unsuitable for use. Additionally, according to manual experience and classical anchor layouts, it is more mechanical and archaic, which cannot guarantee the universality and specificity [30]. Optimizing the anchors layout or configuration in accordance with the varied physical contexts and accuracy requirements is essential for the widespread usage of UWB positioning systems. The magnitude of the horizontal dilution of precision (HDOP) and the scope of the locatable space represent the impact of the UWB anchors layout or configuration on positioning accuracy in various application situations. Existing UWB anchors deployment research splits into two separate strands, with few studies jointly optimizing coverage and HDOP performance. Jiang et al. [32] proposed a three-stage HDOP-evaluated framework for automated valet parking, cutting anchor count by 27% while meeting accuracy specs, but treated HDOP as a post-validation metric rather than a co-optimization constraint. Wang et al. [33] confirmed HDOP is unsuitable as a standalone optimization target, yet did not tie it to coverage requirements. Yu et al. [34] used a fused annealing-genetic algorithm with 3D coverage as the sole fitness function, entirely excluding HDOP from the optimization loop. Current approaches either prioritize geometric accuracy at the cost of full coverage, or guarantee coverage while leaving positioning accuracy uneven across the area. A unified framework balancing both factors remains lacking for economical, robust UWB deployment.

In this research, the proposed SMLS-PSO algorithm integrates four straightforward and effective LPSO search strategies. To self-adaptively and dynamically control the adoption probability of each learning strategy, we propose a novel self-adaptive strategy selection mechanism that gradually determines the suitable learning strategy to update each particle’s velocities and position according to the fitness information payoff at different evolution stages. To further enhance performance, three auxiliary optimization mechanisms are incorporated. First, a stagnation counter is established for each particle. When the counter exceeds the threshold parameter, the GOBL strategy will be used for the particles, which can reduce the waste of fitness evaluation before the local search optimum. Secondly, a boundary symmetry mapping method is used to handle out-of-range search to avoid particles crossing the boundary and even leading to search divergence. Lastly, we incorporate the Quasi-Newton local search operator to boost the local exploitation capability in the later stages of evolution. To assess the effectiveness of the proposed SMLS-PSO algorithm, we conducted test experiments on 13 basic benchmark functions and the CEC2017 test suite. The results were compared with those of its constituent PSO variants and eight cutting-edge PSO variants. Finally, the real-world layout problem of UWB anchor nodes is formulated as an optimization objective or fitness function, and the proposed SMLS-PSO algorithm is applied to obtain the optimal deployment parameters, which is beneficial for the positioning availability of UWB in real environments.

## 2. Related Work

### 2.1. Linearly Decreasing Inertia Weight PSO (LDWPSO)

In LDWPSO [35], each particle is treated as a separate solution with its own position and velocity. Each population individual gravitates toward the optimal position based on its best historical experiences, *pbest* and *gbest*, of the whole population. The particle swarm evolution equation is as follows:

(1) $$v_{i}^{d}(t+1)=\omega *v_{i}^{d}(t)+c_{1}*r_{1}*[{\mathrm{pbest}}_{i}^{d}(t)-x_{i}^{d}(t)]+c_{2}*r_{2}*[{\mathrm{gbest}}_{i}^{d}(t)-x_{i}^{d}(t)]$$

(2) $$x_{i}^{d}(t+1)=x_{i}^{d}(t)+v_{i}^{d}(t+1)$$

where d=1,2,…,D is the dimension of the optimization problem. The *i*th particle position and velocity are $x_{i}^{d}(t)$ and $v_{i}^{d}(t)$ (i=1,2,…,N), respectively. ω is the inertia weight parameter, $c_{1}$ and $c_{2}$ are the acceleration coefficients, and $r_{1}$ and $r_{2}$ are two random numbers generated within the range of [0, 1]. In addition, $pbest_{i}^{d}$ and ${gbest}^{d}$ are the *pbest* and *gbest* position for the *i*th particle in the *d*th dimension. The particle velocity update is divided into three components in this manner. The particle’s inertia, which keeps it moving at its former speed and demonstrates “memory,” is the first component. The second component, known as “cognition,” represents the particle’s own knowledge. The third component, referred to as “socialization,” is the information exchange and teamwork amongst particles.

### 2.2. Social Learning PSO

A unique social learning PSO was proposed by Cheng et al. (SL-PSO) [13]. PSO’s *pbest* and *gbest* are abandoned by SLPSO, which instead draws its learning from the average position of the current population (Average) and better particles (demonstrators) that are each better than it in one dimension. This method of learning clearly reflects sociality. Initially, the population is arranged according to the fitness values of the individual particles. Subsequently, each imitator particle $x_{i}(t)$ (1 ≤ i < N) will draw knowledge from its respective distinct demonstrators $x_{k}(t)$ (i < k ≤ N) in the following manner:

(3) $$x_{i}^{d}(t+1)=\left\{\begin{array}{ll} x_{i}^{d}(t)+\Delta x_{i}^{d}(t+1), & if\;\;\;p_{i}(t)\leq PL_{i}\\ x_{i}^{d}(t), & otherwise \end{array}\right.$$

where $PL_{i}$ is the learning probability for each particle $x_{i}(t)$, $\Delta x_{i}^{d}(t+1)$ is the behavior correction, which consists of three components as follows:

(4) $$\Delta x_{i}^{d}(t+1)=r_{1}\cdot \Delta x_{i}^{d}(t)+r_{2}\cdot I_{i}^{d}(t)+r_{3}\cdot \varepsilon \cdot C_{i}^{d}(t)$$

with

(5) $$\left\{\begin{array}{l} I_{i}^{d}(t)=x_{k}^{d}(t)-x_{i}^{d}(t)\\ C_{i}^{d}(t)={\bar{X}}^{d}(t)-x_{i}^{d}(t)\\ \varepsilon =\beta \times D/N\\ {\bar{X}}^{d}(t)=\sum_{i=1}^{N}x_{i}^{d}(t)/N \end{array}\right.$$

where $r_{1}$, $r_{2}$ and $r_{3}$ are the random values within [0, 1]. In the imitation component $I_{i}^{d}(t)$, $x_{k}(t)$ represents the *d*-th dimension in the behavior vector of particle k. In the social influence component $C_{i}^{d}(t)$, ${\bar{X}}^{d}(t)$ is the mean behavior of all individuals. ε is the society influence factor, and β is a predefined parameter.

From Equation (4), the first item is the random inertia component, which has strong randomness and is responsible for increasing population diversity. The second item is the imitation component, in which a particle $x_{k}(t)$ is selected (k is selected randomly) to improve the population diversity of PSO, too. The third element serves as the societal impact component, primarily facilitating the sharing of population information. Overall, SLPSO addresses the issue of PSO’s tendency to become trapped in local optima and boasts robust global search capabilities. The third item is the social influence component, which is mainly used to share the information of the population. In general, SLPSO has strong global search capability.

### 2.3. Comprehensive Learning PSO

Particles in the standard PSO gain knowledge from *pbest* and *gbest* positions. All individuals will quickly fall into the local optimum and have a tendency to convergence prematurely if *gbest* does not arrive at the ideal solution. In CLPSO [10], all particles update their velocity by considering both their *pbest* position and the *pbest* of other particles across different dimensions according to Equation (6):

(6) $$v_{i}^{d}(t+1)=\omega *v_{i}^{d}(t)+c_{1}*r_{1}*[{\mathrm{pbest}}_{\mathrm{fi}(d)}^{d}(t)-x_{i}^{d}(t)]$$

where $f_{i}(d)=[f_{i}(1),f_{i}(2),\ldots f_{i}(D)]$ determines which particle’s *pbest* is to be followed in the *d*th dimension. Each individual has a distinct learning probability value, denoted as *Pc*, which is computed using Equation (7):

(7) $$Pc_{i}=a+b*\frac{\left(\exp\left(\frac{10(i-1)}{ps-1}\right)-1\right)}{\left(\exp(10)-1\right)}$$

where “*ps*” represents the population size, a=0.05, and b=0.45. A tournament selection method is employed to select the guiding particle. The specific or detailed implementation process can be referred to in the original literature. Accordingly, the learning example $pbest_{\mathrm{fi}(d)}^{d}$ is a new position that channels the particles in the search space into a new orientation. The refresh gap m is defined as the number of evaluations that must pass in order to prevent the function evaluations from being squandered in the wrong direction. A fresh $pbest_{\mathrm{fi}(d)}^{d}$ will be generated until the particle stops improving if the subsequent moves in generation m are not improved. In this manner, the flying direction of the particle is updated using the most accurate historical data available for all particles. This guarantees that the population diversity can be preserved by the CLPSO algorithm and premature convergence can be avoided. The test shows that while the CLPSO is less effective at handling unimodal problems, it has clear advantages for solving multimodal challenges.

### 2.4. Generalized Opposition-Based Learning PSO

Tizhoosh initially introduced opposition-based learning (OBL) [36] back in 2005, and it has been effectively used to solve a number of optimization issues. Computing the opposite solution to a problem while analyzing a solution x will give researchers another opportunity to identify a potential solution that is nearer the overall optimum. Let $X_{i}=(x_{i}^{1},x_{i}^{2},\ldots ,x_{i}^{D})$ be an D-dimension vector, where $x_{i}^{d}\in \left[a_{i}^{d},b_{i}^{d}\right]$, d∈1,2,…,D. The opposite number vector $X_{i}^{*}=(x_{i}^{1*},x_{i}^{2*},\ldots ,x_{i}^{D*})$ is defined as:

(8) $$X_{i}^{*}=a_{i}+b_{i}-X_{i}$$

If $f(X_{i}^{*})$ is better than $f(X_{i})$, then update $X_{i}$ with $X_{i}^{*}$; otherwise keep the current vector $X_{i}$. In the OBL strategy, we can find that the search space boundary is fixed with $x_{i}^{d*},x_{i}^{d}\in \left[a_{i}^{d},b_{i}^{d}\right]$. The opposite solution is dispersed over a large region and is adverse to the optimal solution at a later stage in evolution when all particles may focus on a limited search space. Based on the aforementioned issues, Wang et al. [12] presented a generalized opposition-based learning technique (GOBL) that uses dynamically updated interval boundaries to calculate opposite particles as follows:

(9) $$\left\{\begin{array}{l} X_{i}^{*}(t)=k[a_{i}(t)+b_{i}(t)]-X_{i}(t)\\ a_{i}(t)=\min(X_{i}(t)),b_{i}(t)=\max(X_{i}(t)),\\ X_{i}^{*}(t)=rand(a_{i}(t),b_{i}(t)),If\;\;\;X_{i}^{*}(t)<X_{\min}||X_{i}^{*}(t)>X_{\max} \end{array}\right.$$

where *k* is a random value in [0, 1]. $a_{i}(t)$ and $b_{i}(t)$ are the minimum and maximum values of the *d*th dimension in the current population, respectively. $rand(a_{i}(t),b_{i}(t))$ is a random value in $\left[a_{i}(t),b_{i}(t)\right]$, $\left[X_{\min},X_{\max}\right]$ is the boundary constraint. By using the GOBL strategy in the population, we can obtain fitter candidate solutions.

### 2.5. Dimension Learning PSO

Xu et al. [15] proposed Dimension Learning PSO (DLPSO), in which the person best experience *pbest* learns from the population best position *gbest* in a way of dimension by dimension to construct a learning exemplar $pe\_best_{i}^{d}$. This strategy allows the excellent information of *gbest* to be flowed to the exemplar $pe\_best_{i}^{d}$, promoting the spreading and protecting of *gbest*, avoiding some dimension degeneration which results in the phenomenon of “two steps forward, one step back” and “oscillation”. DL strategy guarantees that $pe\_best_{i}^{d}$ is not worse than the canonical PSO *pbest*, whose velocity update formula is as follows:

(10) $$v_{i}^{d}(t+1)=\omega *v_{i}^{d}(t)+c_{1}*r_{1}*[{\mathrm{pe}\_\mathrm{best}}_{i}^{d}(t)-x_{i}^{d}(t)]+c_{2}*r_{2}*[{\mathrm{gbest}}_{i}^{d}(t)-x_{i}^{d}(t)]$$

where $pe\_best_{i}^{d}$ is the *i*th particle person best position learning exemplar, taking the temporary exemplar $pbest_{i}^{d}$ learns only from the dimension of the population best position $gbest_{i}^{d}$, if the fitness of the temporary exemplar surpasses that of the current learning exemplar, the learning exemplar is then updated to match the temporary exemplar; otherwise, the current learning exemplar does not change and continues learning for the next dimension. The search procedure for the DL exemplar is depicted in Algorithm 1.
**Algorithm 1** Pseudo code for generating a DL exemplar $pe\_best_{i}^{d}$ for particle *i*1:**If** flag(*i*) = 1 2:    $pe\_best_{i}=pbest_{i};$
3:    **For** each dimension *d* **do**4:        $temp^{d}=pe\_best_{i}^{d}$
5:        **If** $temp^{d}=gbest^{d}$ **then**6:              **continue**7:        **End if**8:$temp^{d}=gbest^{d}$9:Evaluate f(temp)
10:        **If**
$f(temp)<f(pe\_best_{i})$ **then**11:              $pe\_best_{i}^{d}=gbest^{d}$
12:**End if**13:**End for**14:**End if**

From Equation (10), we can know that when particles adopt the DL strategy learn not only from the exemplar $pe\_best_{i}^{d}$ in which the excellent information of the entire population’s best experience $gbest_{i}^{d}$ is embedded, but also learn from $gbest_{i}^{d}$. Therefore, the DLS particles have stronger exploitation ability. Therefore, the learning exemplar $pe\_best_{i}^{d}$ could ensure that the current learning exemplar $pe\_best_{i}^{d}$ will not be degraded.

## 3. The Proposed SMLS-PSO Method

The primary objective of the self-adaptive multiple learning strategy framework introduced in this paper is to enhance the versatility or general applicability of PSO when tackling a diverse array of problems with distinct characteristics. The core concept is to regulate numerous efficient velocity update methods for learning strategies in parallel and adaptively. More specifically, different execution probabilities can be assigned to various learning strategies at various stages of the evolutionary process for different problems, such as unimodal or multimodal problems, depending on the feedback of solution quality or payoff previously generated to prevent premature convergence or getting trapped in local optima.

In general, four learning strategies were chosen because they each offer unique advantages and qualities that may be used to address a variety of topics. Prior to introducing the SMLS-PSO algorithm, we first discuss the characteristics of the four potential velocity updating strategies that will be used in SMLS-PSO: SL-PSO, CLPSO, GOPSO, and DLPSO.

- SL-PSO is very different from traditional PSO, which updates velocity based on historical information. SL-PSO exhibits strong social characteristics. In this approach, each particle is capable of drawing knowledge from any superior particle within the current group. This learning process occurs without the need for individual trial and error, thus avoiding the associated costs. It can trade off exploration and exploitation and solve many problems with much higher convergence speed and reliable.
- CLPSO has proven to have decent exploration ability. Despite having a poor convergence speed for unimodal problems, it is very effective at addressing multi-modal situations.
- GOPSO and DLPSO are specifically created to produce better exploitation but slightly lower exploration abilities. They can quickly converge for unimodal problems but are easy to enter local optima for multimodal problems.

### 3.1. Self-Adaptive Selection Mechanism

Four state-of-the-art learning-series PSO versions, SL-PSO, CLPSO, GOPSO, and DLPSO, are used in the proposed SMLS-PSO algorithm. Utilizing self-adaptive multi-learning strategies serves the purpose of selecting the best learning techniques for various tasks based on feedback from prior performance. The core concept of the self-adaptive multi-learning strategy selection framework used in SMLS-PSO is similar to that of SLPSO [22], although it is implemented differently. Four PSO-based update algorithms were used by SLPSO adaptively in a dynamic manner. To assign weights to particles during the learning period (LP), SLPSO sorts particles based on fitness values. A probability model was utilized. It quantified the likelihood of each of the four options being chosen. The weakness of SLPSO is that it did not update the adoption probability of the strategies using the fitness improvement information, which prevents it from fully utilizing the unique properties of various strategies. By using fitness improvement information to update each strategy’s execution probability, we present a novel execution probability selection strategy in this study. One of the advantages of this study is that the fitness improvement values that the selection strategy calculated are used to assess each learning strategy’s performance. The following steps are a full presentation of the self-adaptive selection technique in SMLS-PSO:

**Step I:** Each learning strategy is initially given equal execution probabilities $proSTR_{k}=0.25,k=1,2,\ldots ,4$ and set fitness improvement accumulators of four learning strategies $\Delta F_{k}=0$. The roulette wheel selection rule is employed to pick the candidate learning strategy for each individual, based on proSTR.

**Step II**: A particle updates its position by using learning strategy k at each generation t. If the *pbest*_i_ fitness successful update within a learning period (LP), the fitness improvement values are added to the accumulators $\Delta F_{k}$ of their associated updating strategies as follows to evaluate the reward of each learning strategy:

(11) $$\Delta F_{k}(t)=\Delta F_{k}(t)+\frac{f(pbest_{i})-f(x_{i})}{f(pbest_{i})}$$

**Step III**: If the *k*th strategy improvement is $\Delta F_{k}=0$, set $\Delta F_{k}=0.1*\min(\left[\Delta F_{1},\ldots ,\Delta F_{4}\right])$ after a predetermined number of generations LP in order to prevent potential strategy improvements equaling zeros. Set $\Delta F_{k}=1,k=1,\ldots ,4$ if all strategy improvements are zero. Each strategy fitness improvement will then be standardized to $p_{k}\in \left[0,1\right]$ as follows:

(12) $$p_{k}=\frac{\Delta F_{k}(t)}{\sum_{k=1}^{4}\Delta F_{k}(t)}$$

**Step IV**: The formula for updating the execution probability of selecting learning strategies from the behavior pool is as follows:

(13) $$proSTR_{j}^{\prime }=(1-r)proSTR+r\cdot p_{k}$$

In Equation (13), r=1/6 is the learning coefficient, which controls the updating proportion, in a manner similar to the SLPSO [22]. The learning techniques that result in high-payoff solutions should gradually enhance the likelihood that they will be chosen. In other words, the self-adaptive selection strategy eventually chooses the best PSO approach for a particular problem.

### 3.2. Some Improved Approaches in SMLS-PSO

#### 3.2.1. Added Stagnation Threshold Parameters in GOPSO

In the GOPSO algorithm, the GOBL approach is applied to every particle in each generation, leading to considerable resource expenditure on fitness evaluations before particles potentially become trapped in local optima. We recommend incorporating the stagnated counter parameter *oblCounter* for all particles in order to reduce the waste of fitness evaluation before the local search optimum. The *oblCounter* will be incremented if the particle’s *pbest* position update stagnates. When the particle position stagnates beyond the OBL_limit threshold, the GOBL technique will update the particle’s position. A suitable OBL_limit parameter is beneficial for obtaining a better solution, and its specific value will be discussed in Section 5.2.

#### 3.2.2. Out-of-Range Search Handling in SMLS-PSO

All swarm intelligence optimization algorithms encounter the issue of out-of-range search or boundary crossing. This problem significantly affects the algorithm’s performance. If not addressed promptly, it can cause the algorithm to diverge rapidly. Conversely, if not handled properly, it may lead to premature convergence. Nowadays, most swarm intelligence optimization algorithms handle out-of-range searches of individual places by applying the simple rule “if it goes beyond the boundary, it is equal to the boundary”. This method has the advantage of being simple to use, but because the boundary point is certainly not the optimal solution overall, it is harmful to the optimization process.

To tackle out-of-range search in SMLS-PSO, we introduce a novel approach based on boundary symmetry mapping. Suppose $\left[X_{\min}^{d},X_{\max}^{d}\right]$ is the search boundary in the *d*th dimension of a given issue. If the current location $x_{i}^{d}(t)$ exceeds the boundary, update the current position as follows:

(14) $$x_{i}^{d}{\left(t\right)}^{\prime }=\left\{\begin{array}{c} \min(X_{\max}^{d},2X_{\min}^{d}-x_{i}^{d}(t)),if\;\;\;x_{i}^{d}(t)<X_{\min}^{d}\\ \max(X_{\min}^{d},2X_{\max}^{d}-x_{i}^{d}(t)),if\;\;\;x_{i}^{d}(t)>X_{\max}^{d} \end{array}\right.$$

**Figure 1:**
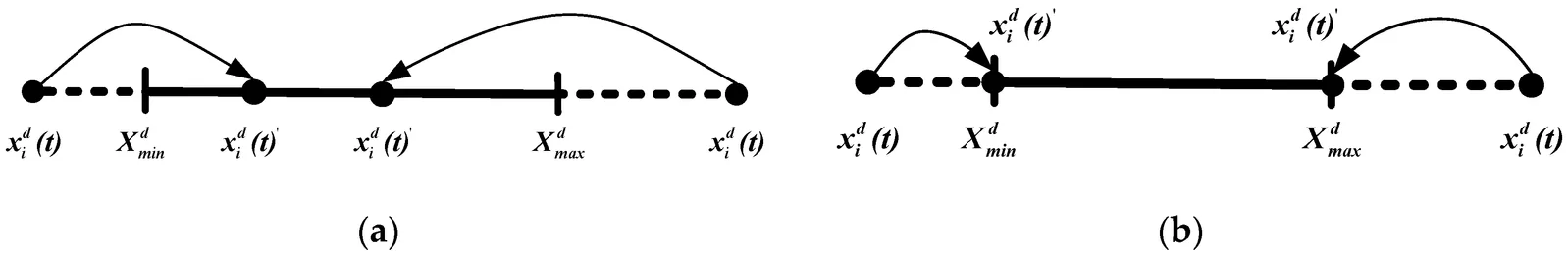
Comparison of two methods of transboundary treatment. (**a**) Boundary symmetry mapping transboundary handling method; (**b**) Ordinary PSO transboundary handling method.

The difference between the boundary-symmetric mapping processing method and the ordinary PSO transboundary handling strategy is shown in Figure 1. The boundary symmetric mapping method induces the out-of-range search particles to immediately return to the optimization space instead of remaining at the boundary. Additionally, this technique speeds up optimization even more.

#### 3.2.3. Quasi-Newton Local Search Operator

We incorporate the widely used BFGS Quasi-Newton method [37,38] in SMLS-PSO as a local search operator. Only *gbest* is chosen to conduct local searches throughout the final stages of development. We assign [0.05 ∗ MaxFEs] evaluations to refine *gbest* in the local-searching operator using the BFGS Quasi-Newton approach. The BFGS Quasi-Newton local-searching operator is implemented in this research using the function “*fminunc*” in MATLAB R2021a to make things simpler.

### 3.3. Self-Adaptive Multi-Learning Strategy PSO

The proposed SMLS-PSO algorithm’s detailed pseudocode is provided in Algorithm 2, along with the aforementioned properties. The MATLAB source code for SMLS-PSO can be accessed by contact to corresponding author.
**Algorithm 2** The pseudocode of the proposed SMLS-PSO algorithm1:Initialize parameter for SL-PSO, CLPSO, GOPSO, and DLPSO;2:Set generation *iter* = 1, learning period *LP* = 10, learning coefficient r=1/6;
3:Set the selection probability of 4 learning strategy $proSTR_{k}=0.25,k=1,\ldots ,4;$
4:Set the fitness improvement accumulators $\Delta F_{k}=0,k=1,\ldots ,4.$
5:Initialize all particles positions *X_i_* and velocity *V_i_* (*i* = 1,2,…, *N*);6:Evaluate the fitness values $Fit=\left[fit_{1},fit_{2},\ldots ,fit_{ps}\right],$ let *fes* = *N*;7:Update the particles’ *pbest_i_* = *X_i_* and *gbest = min*(*pbest_i_*);8:Initialize the CL exemplar and DL exemplar;9:**While** *fes* < 0.95 × MaxFEs **do**10:    **For** *i* = 1:*N*11:        Select the *k*th learning strategy by roulette wheel selection based on *proSTR*;12:        Update the position *X_i_* and velocity *V_i_* by the selected strategy;13:        Out-of-Range Search Handling by Equation (14);14:        Evaluate the fitness value $fit_{i}$ of the new particle *X_i_*, *fes* = *fes*+1;15:        Update *pbest_i_* and *gbest;*16:        Update fitness improvement by Equation (11);17:    **End for**18:    **If** mod(*iter*, LP) = = 019:        Update the standardized strategy improvement values *p_k_* by Equation (12);20:        Update $proSTR_{j}$ by Equation (13);21:        Set $\Delta F_{k}=0,$
k=1,…,4
22:    **End if**23:    *iter* = *iter* + 1; 24:**End while**25:**While** *fes* < MaxFEs **do**26:        Refine the gbest position adopt the BFGS Quasi-Newton local search operator.27:**End while**

### 3.4. Comparison of the Adaptive Strategy-Selection Mechanisms

In order to highlight the distinctive features of our adaptive selection mechanism compared with SLPSO, EPSO, and SDPSO, we examined the core components of these four algorithms and prepared a systematic comparison table (see Table 1 below). This table clarifies the essential differences in five key dimensions: (i) strategy pool composition, (ii) payoff/reward calculation, (iii) probability update rule (with or without inertia), (iv) learning period (LP), and (v) auxiliary mechanisms and population structure.

There are four aspects in novelty and essential differences of the proposed SMLS-PSO selection mechanism:

(1) Unlike SLPSO’s ranking weights and EPSO’s binary success/failure records, SMLS-PSO uses the sum of actual *pbest* improvements as the reward. This captures the magnitude of improvement rather than just the occurrence, providing a more accurate measure of each strategy’s contribution, while being simpler than SDPSO’s average-improvement method, which requires an extra division by evaluation count.
(2) Our update rule retains an inertia term (r = 1/6), avoiding the abrupt fluctuations of EPSO’s memory-less replacement. Compared with SLPSO (which only relies on fitness ordering), our method directly reflects the optimization payoff. In contrast to SDPSO’s replicator-dynamics equation, our formula is more straightforward and computationally lighter.
(3) Unlike EPSO and SDPSO, which employ two sub-populations or complex multi-swarm designs, SMLS-PSO uses a single swarm in which all particles share the same strategy probabilities. This reduces structural overhead and parameter tuning, while the stagnation-triggered GOBL mechanism effectively compensates for potential diversity loss.
(4) When a particle’s *pbest* fails to improve for a predefined number of generations, the GOBL operator is activated to generate a new candidate solution. This auxiliary step is not present in any of the compared adaptive PSO variants and serves to reduce wasted fitness evaluations while enhancing local exploitation.

Overall, SMLS-PSO distinguishes itself from SLPSO, EPSO, and SDPSO through its reward granularity, update smoothness, structural simplicity, and the unique stagnation-driven opposition-based correction.

## 4. UWB Indoor Positioning Anchor Layout Optimization Model

UWB indoor positioning anchor layout optimization problem can be modeled as a single-objective optimization problem and then solved using the proposed SMLS-PSO algorithm outlined in Section 3. The key lies in establishing a suitable fitness function model, and this section proposes a new model for UWB anchor layout optimization, taking into account the locatable space coverage rate and HDOP value.

### 4.1. Locatable Space Coverage Rate

In UWB 2D indoor positioning, the locatable space coverage rate of UWB signals is a significant indicator. The mobile tag must receive at least three LOS signals to be considered a valid positioning point; otherwise, it is considered a blind positioning point. In this paper, a planar grid segmentation method is used to segment the indoor positioning area, and the indoor spatial positioning signal coverage is calculated using the K-coverage model [39,40].

Suppose the area of the indoor positioning plane is S, the length is L, the width is W, and the length of the small grid side is l, and that the indoor plane area S is divided into M∗N small grids, namely, M=L/l and N=W/l. Let the central coordinate of each grid be $C_{m,n}(x_{m},y_{n})$, m=(1,…,M),n=(1,…,N). The grid area is replaced by the coordinates of this center point; that is, the coverage rate of each small grid area is calculated using center point $C_{m,n}(x_{m},y_{n})$ approximation. Set $Anchor=(BS_{1},BS_{2},\ldots ,BS_{N})$ as the location set of UWB anchors. The UWB anchor ranging coverage radius is R and $d_{i}=\sqrt{{\left(X_{i}-x_{m}\right)}^{2}+{\left(Y_{i}-y_{n}\right)}^{2}}$ is the distance between $BS_{i}(X_{i},Y_{i})$ and grid point $C_{m,n}(x_{m},y_{n})$ if $d_{i}\leq R$ indicates that grid point $C_{m,n}$ is covered by $BS_{i}$. When the effective coverage of the anchor for this grid point is greater than 3, the grid point can be located, and the ratio of all the locatable grid points is calculated as the whole space coverage rate, as shown in Equations (15) and (16)

(15) $$cov(m,n)=\left\{\begin{array}{ll} 1, & E\geq 3\\ 0, & E<3 \end{array}\right.$$

(16) $$cover\_rate=\frac{\sum_{P\in M\times N}cov(m,n)}{M\times N}$$

### 4.2. UWB HDOP Calculation Principle

**Figure 2:**
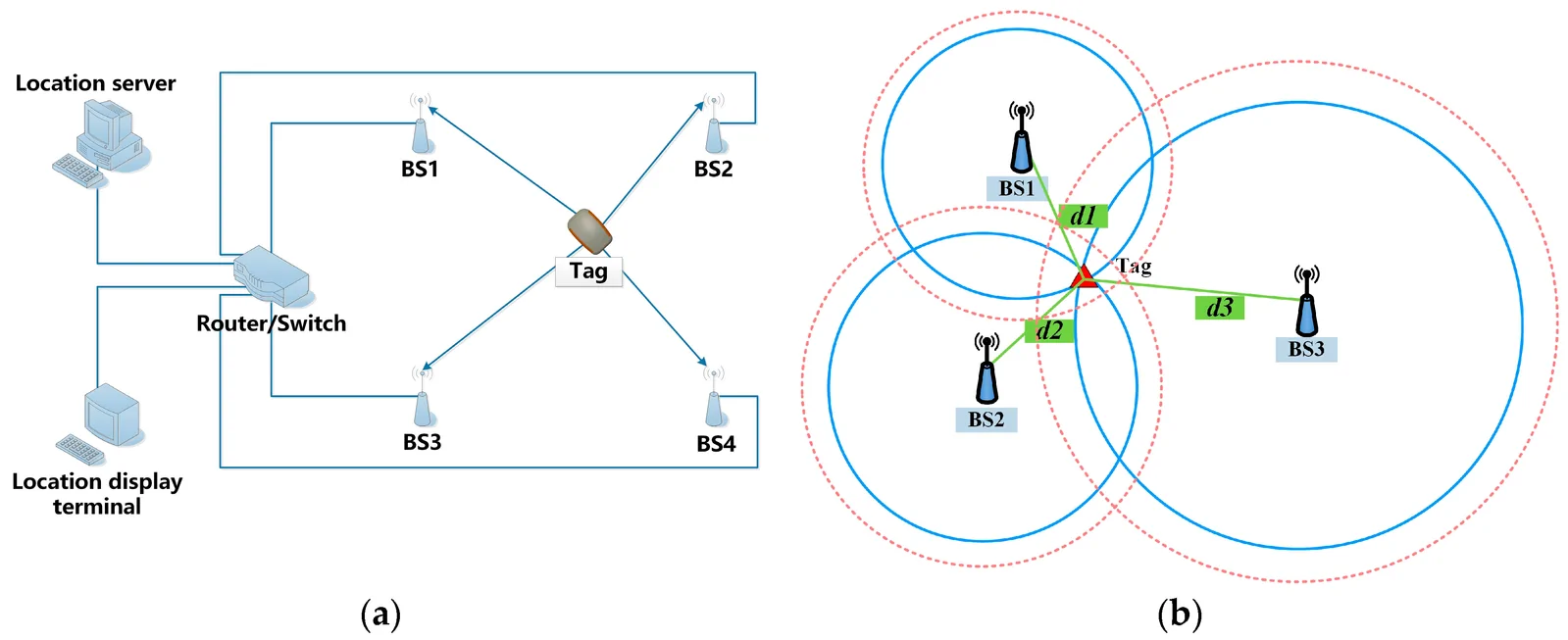
UWB indoor position system (**a**) and measurement range value positioning principle (**b**). The blue solid line in (**b**) represents the true value of the measurement range; ideally, the three circles meet at a point, and the pink dashed line represents the actual measurement range value containing the error, and the three circles meet to form a region.

UWB positioning systems are widely used to obtain mobile tag positions using Time-of-Arrival (TOA)/Time-of-Flight (TOF)/two-way-ranging (TWR) measurements. As shown in Figure 2, the basic UWB positioning principle using TOF in the 2D plane is the intersection of multiple circles to calculate Tag position coordinates.

Set $BS_{i}(X_{i},Y_{i}),i=1,2,\ldots ,N$ as the location of the anchor. The tag’s location is Tag(x,y), and its measurement distance to the BS is $d_{i}$, respectively. At least three anchors are needed to establish the following observation equations:

(17) $$\left\{\begin{array}{c} d_{1}=\sqrt{{\left(X_{1}-x\right)}^{2}+{\left(Y_{1}-y\right)}^{2}}+\varepsilon_{1}\\ d_{2}=\sqrt{{\left(X_{2}-x\right)}^{2}+{\left(Y_{2}-y\right)}^{2}}+\varepsilon_{2}\\ \vdots \\ d_{i}=\sqrt{{\left(X_{i}-x\right)}^{2}+{\left(Y_{i}-y\right)}^{2}}+\varepsilon_{i} \end{array}\right.$$

The approximate position of tag $\left(x_{0},y_{0}\right)$ can be obtained using Caffery’s Linear Localization with Optimization algorithm (Caffery-LLOP) [41], and Equation (17) is linearly expanded by the Taylor series, retaining the first-order terms to obtain the following formula:

(18) AX=l,P

where $A=\left[\begin{array}{ll} \frac{X_{1}-x_{0}}{d_{1}^{0}} & \frac{Y_{1}-y_{0}}{d_{1}^{0}}\\ \frac{X_{2}-x_{0}}{d_{2}^{0}} & \frac{Y_{2}-y_{0}}{d_{2}^{0}}\\ \vdots & \vdots \\ \frac{X_{i}-x_{0}}{d_{i}^{0}} & \frac{Y_{i}-y_{0}}{d_{i}^{0}} \end{array}\right]$, $X=\left[\begin{array}{c} dx\\ dy \end{array}\right]$, and $l=\left[\begin{array}{c} d_{1}-d_{1}^{0}\\ d_{2}-d_{2}^{0}\\ \vdots \\ d_{i}-d_{i}^{0} \end{array}\right]$. The weighted least squares algorithm in Equation (19) is adopted to iterate calculations until the threshold value is satisfied. The UWB positioning error is affected by the geometric deployment of the anchor in addition to the ranging error. Equation (20) gives the formula for calculating the HDOP value, which is essentially the amplification effect of the final positioning error on the ranging error.

(19) $$X={\left(A^{T}PA\right)}^{-1}A^{T}Pl$$

(20) $$HDOP=\sqrt{tr{\left(A^{T}A\right)}^{-1}}$$

### 4.3. UWB Anchor Layout Optimization Fitness Function Model

This paper proposes an optimization *fitness* function for UWB anchor layout by weighting the locatable spatial coverage rate and the HDOP value, that is:

(21) $$fitness=\omega_{1}\times \frac{1}{cover\_rate}+\omega_{2}\times HDOP$$

where $\omega_{1}\in \left[0,1\right]$ and $\omega_{2}\in \left[0,1\right]$ represent the weight parameters for the locatable spatial coverage rate and the average HDOP value, which is empirically determined to be the most appropriate for achieving both high coverage and reliable accuracy in typical UWB indoor positioning scenarios, as demonstrated by the experimental evidence. Different weights indicate the importance and priority of different indicators, and they can be adjusted and combined as needed according to one’s own requirements. For instance, in scenarios where ultra-high precision is paramount regardless of minor coverage loss, the weight for HDOP could be increased.

## 5. Experimental Results and Discussions

### 5.1. Experimental Setting

In this research, the performance of the proposed SMLS-PSO algorithm is evaluated using a set of 42 test functions. These functions consist of 13 basic benchmark functions (detailed in Appendix A Table A1), which are categorized into 8 unimodal functions and 5 multimodal functions. Additionally, 29 more functions are selected from the comprehensive test suite of the CEC2017 special session on real-parameter optimization [42]. The search scope is ${\left[-100,100\right]}^{D}$ for all issues. Among these, F1 and F3 serve as unimodal functions; F2 was excluded from our evaluation as it is known to exhibit unstable behavior in high-dimensional search spaces. Moreover, the functions F4 to F10 represent basic multimodal functions. Functions F11 to F20 are hybrid in nature, while functions F21 to F30 are composite functions, combining multiple test challenges into a more intricate and complex environment.

The population size for all compared PSO variants in this work was fixed at 80. The algorithm terminates when the maximum number of fitness evaluations (MaxFEs) reaches 10,000 times the dimension D. All algorithms are implemented in MATLAB R2021a and executed on a laptop with an Intel Core i5 11400 CPU (2.60 GHz) and 8GB RAM, running Microsoft Windows 10. In order to evaluate the performance of the proposed SMLS-PSO against other state-of-the-art PSO variants, the mean error and standard deviation are calculated and subsequently ranked. The ultimate ranking of each algorithm is established based on its mean ranking value. Additionally, a Wilcoxon rank sum test is run at a significance threshold of 0.05 between SMLS-PSO and each comparator algorithm. The symbols “+”, “=” and “−” respectively indicate that the performance of the SMLS-PSO algorithm is significantly better than, without significant difference, and worse than the compared algorithm. To support the experimental results, the Friedman test is also conducted to determine whether the variations in algorithmic results are significant.

### 5.2. Parameter Tuning

Just two parameters—*LP* and *OBL_limit*—need to be changed in the proposed SMLS-PSO algorithm. To ensure the robustness of these settings and prevent overfitting to simple scenarios, we conducted a comprehensive sensitivity analysis using an expanded test suite of 42 benchmark functions. This suite includes the original 13 basic functions plus 29 complex functions from the CEC2017 benchmark, covering unimodal, multimodal, composition, and hybrid landscapes. The problem dimension, maximum function evaluations (MaxFEs), and number of independent runs were set to 30, 300,000, and 30, respectively. To isolate the effects of these parameters, the embedded BFGS Quasi-Newton local search component was temporarily disabled during this tuning phase. First, with *OBL_limit* fixed at 30, we evaluated *LP* values ranging from 3 to 50. As summarized in Table 2, while lower *LP* values generally performed well, the statistical ranking across the 42 functions revealed that *LP =* 7 achieved the best overall performance. Although *LP =* 5 showed competitive results on simpler functions, *LP =* 7 demonstrated superior stability and effectiveness on the more complex CEC2017 problems. Consequently, *LP =* 7 was selected as the default setting for the subsequent experiments.

Second, the *OBL_limit* parameter governs the computational budget allocated to the GOBL strategy, where an inappropriate value may hinder convergence. We therefore assessed *OBL_limit* values ranging from 10 to 50. As presented in Table 3, *OBL_limit =* 30 emerged as the optimal setting across the entire 42-function suite. While *OBL_limit =* 20 also performed competitively on some functions, it yielded the worst solutions for specific cases (e.g., F13 in the basic classical function and F10–F11, F15–F17, F22 and F26 in C × 10C2017). Specifically, *OBL_limit =* 30 yielded the lowest frequency of worst-case rankings across the 42 test functions, providing the most reliable trade-off between exploration and exploitation. Thus, *OBL_limit =* 30 was adopted as the optimal choice for the proposed algorithm.

### 5.3. Search Behavior of SMLS-PSO on Simple Benchmark Problems

**Figure 3:**
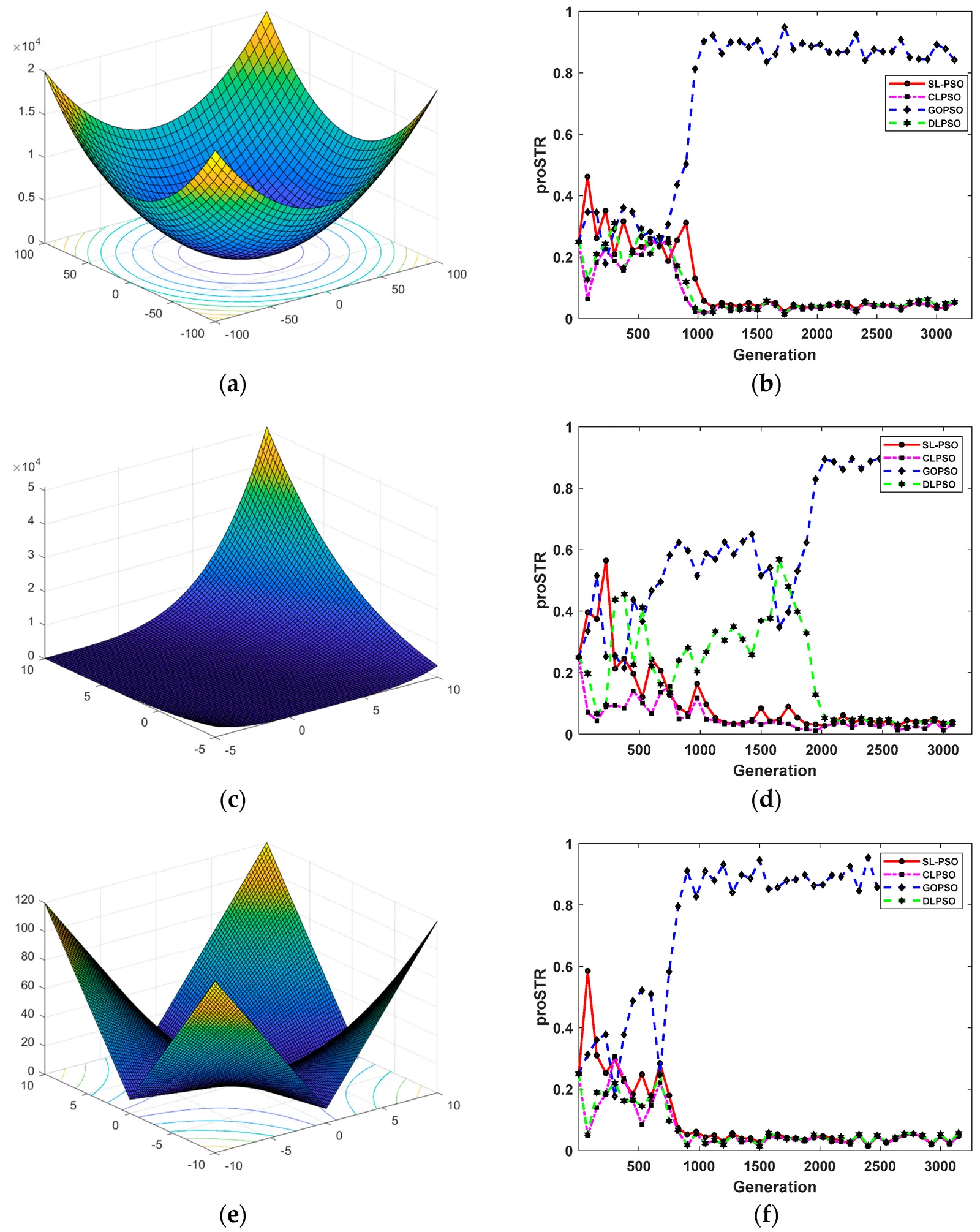
Self-adaptive selection characteristics of average execution probabilities (*proSTR*) using different learning strategies of SMLS-PSO on unimodal benchmark functions. (**a**) F2: Sphere function; (**b**) The *proSTR* curve for F2 function; (**c**) F5: Zakharov function; (**d**) The *proSTR* curve for F5 function; (**e**) F6: Schwefel 2.22 function; (**f**) The *proSTR* curve for F6 function.

**Figure 4:**
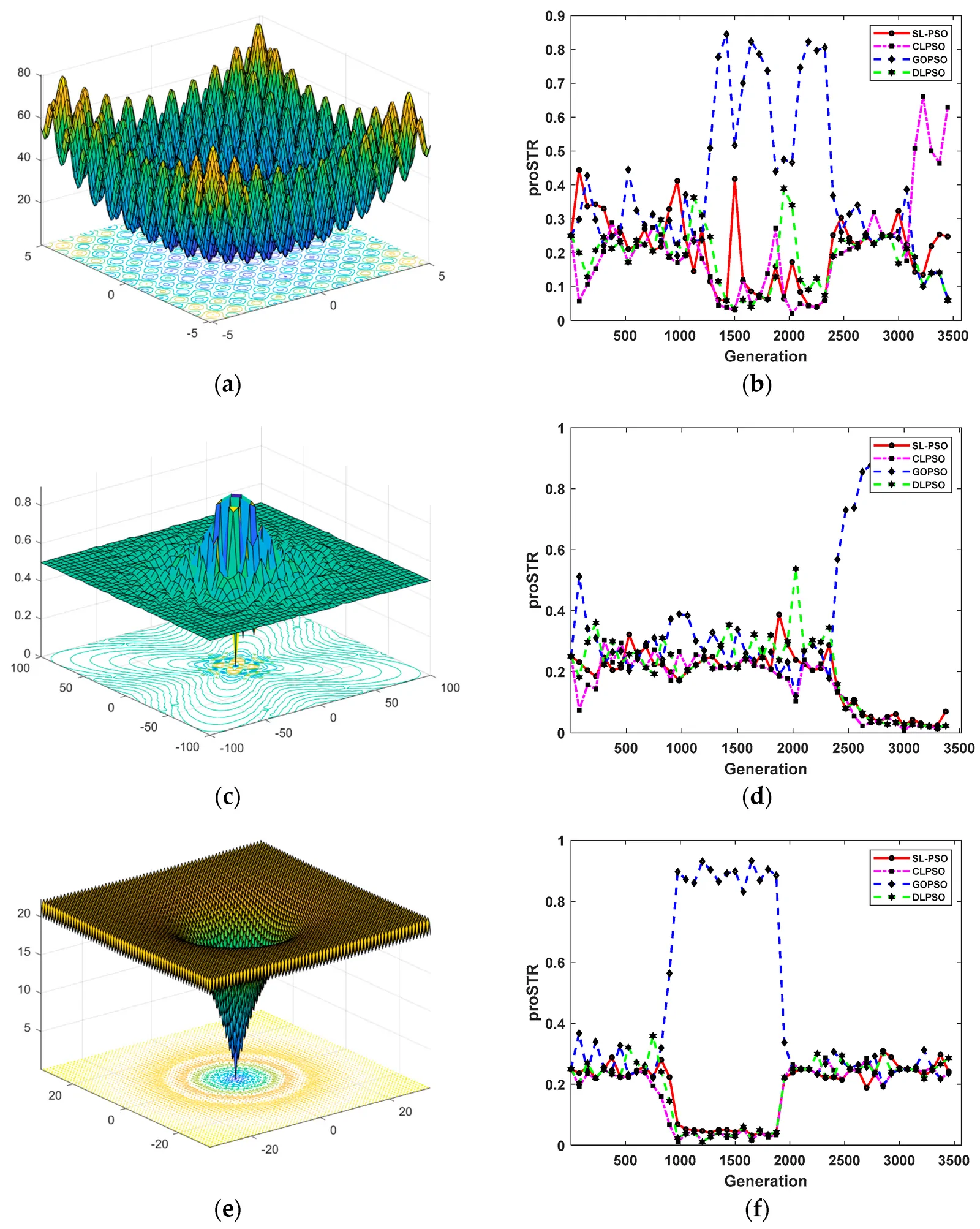
Self-adaptive selection characteristics of average execution probabilities (*proSTR*) using different learning strategies of SMLS-PSO on multimodal benchmark functions. (**a**) F9: Rastrigin function; (**b**) The *proSTR* curve for F9 function; (**c**) F10: Schaffer6 function; (**d**) The *proSTR* curve for F10 function; (**e**) F13: Ackley function; (**f**) The *proSTR* curve for F13 function.

To further comprehend the search behavior and efficacy of the proposed SMLS-PSO’s self-adaptive update mechanism, we first analyzed the execution probabilities (*proSTR*) of the four component learning strategies using six 30D simple benchmark functions. The execution probabilities curve was created using the middle of 31 independently executed benchmark routines. Figure 3 and Figure 4 display the average execution probabilities for each learning approach as well as the 3D maps for the test benchmark function.

In Figure 3a,b,e,f, which shows search behaviors on the F2 and F6 functions, we can see that all learning techniques were chosen early on, and GOPSO was thereafter encouraged to rapidly converge. The likelihood of adopting SL-PSO, CLPSO, and DLPSO at this point soon declines to almost zero. The self-adaptive selection mechanism gives GOPSO higher execution probabilities since they perform better. The Zakharov function fitness landscape appears to be comparatively flat and wide in Figure 3c,d, and GOPSO and DLPSO have a considerably greater selection probability when the generation is less than 2000. The highest selection probability for local exploitation is then held by GOPSO. The SL-PSO and CLPSO strategies have a low probability of being used in all unimodal function tests. In SL-PSO, particles update their positions by referencing superior particles (demonstrators) and the overall trend of the current swarm, instead of relying on the historical best positions as in traditional PSO. Meanwhile, CLPSO incorporates learning from both a particle’s own best position (*pbest*) and the *pbest* of other particles across various dimensions. These distinct learning mechanisms in SL-PSO and CLPSO generally result in a more gradual improvement in performance, rendering these two approaches relatively less effective compared to other strategies.

The Rastrigin function, Schaffer6 function, and Ackley function are example multi-modal problems that have numerous local minima, as opposed to the unimodal test functions, and whose 3D fitness landscape is seen in Figure 4. As depicted in Figure 4a,b, the primary evolutionary phase is characterized by the crossover and oscillation patterns in the execution probabilities of the four learning strategies. Notably, SL-PSO and CLPSO exhibit higher selection probabilities compared to GOPSO and DLPSO, which is beneficial for enhancing population diversity in the latter stages of evolution. Due to the presence of numerous locally optimal basins, as seen in Figure 4c,d, the Schaffer6 function adopts a selection probability that is close to that of the Rastrigin function before 2500 generations. When the basin containing the global optimum is identified, the issue becomes a unimodal one. In this scenario, GOPSO execution probabilities tend to rise in order to achieve rapid convergence. As illustrated in Figure 4e,f, the global optimum of the Ackley function lies within the central basin of the search space, a particularly difficult area to locate. To prevent early entrapment in local optima during the initial stages of evolution (t < 1000), the self-adaptive selection mechanism assigns nearly uniform probabilities to each learning strategy. Upon identifying the basin containing the global optimum, GOPSO significantly boosts its execution probability between 1000 and 2000 generations. Thereafter (t > 2000), all learning strategies converge to suitable selection probabilities to efficiently explore the narrow basin housing the optimal solution. All of the aforementioned findings demonstrate how the self-adaptive selection mechanism may adjust the probabilities for various problem functions depending on fitness information, reward, or feedback at various optimization stages.

### 5.4. Comparisons of SMLS-PSO with Component Algorithms

In this experiment, the performance of the proposed SMLS-PSO is evaluated against its constituent algorithms, namely CLPSO, GOPSO, SL-PSO, and DLPSO. In Table 4, the detailed parameter set of these contrasted component PSO variations is displayed. For all component PSO algorithms, the population size N is configured to match that of the SMLS-PSO.

Table 5 lists the outcomes of the -PSO and four components PSO variants on the 30D basic benchmark issues F1 to F13. SMLS-PSO and each of its component algorithms are compared using a Wilcoxon rank sum test with a 0.05 threshold of significance. The final rank of the algorithm is recorded as the average rank, and a summary of the total number of the symbols “+,” “=”, and “−” is shown at the bottom of each statistics table. Additionally, each function’s best results are bolded and emphasized.

It is evident that SMLS-PSO ranks first overall for the 13 benchmark functions, particularly for F1, F5, F8, F10, and F11, and second overall for F2, F6, F7, F9, and F13. On F1~F3, F6, F7, and F13, SL-PSO yields the optimum solution. When it comes to multimodal functionalities F9 and F12, CLPSO performs optimally. On the unimodal functions F1 and F4, DLPSO produces the best rank.

### 5.5. Comparisons with Eight Advanced PSO Algorithms on 30D and 50D CEC2017 Problems

To further verify the validity and effectiveness of the proposed SMLS-PSO algorithm, we conducted comparisons with eight other state-of-the-art PSO variants using the CEC2017 test suite functions. The LDWPSO [35], OLPSO [11], HCLPSO [20], GL-PSO [14], EPSO [8], TSLPSO [15], HCLDMS-PSO [43], and SDPSO [25] are some of these PSO variants. The specific parameter configurations for all the aforementioned PSO variants, including the SMLS-PSO algorithm, are detailed in Table 6. Among these comparing algorithms, LDWPSO is introduced in Section 2.1. OLPSO creates learning exemplars using an orthogonal learning technique. GL-PSO employs a two-cascading-layer structure to generate high-quality superior exemplars. The first to use the selection, mutation, and crossover operators of a genetic algorithm to generate a promising learning exemplar. The second layer uses the canonical PSO algorithm to update the particle’s position and velocity. HCLPSO, TSLPSO, and HCLDMS-PSO belong to static ensemble approach. EPSO and SDPSO adopt the dynamic multi-strategy ensemble approach to self-adaptive control the probability of component strategies to adapt different problem situations.

#### 5.5.1. Results Analysis for 30D Problems

The following are the unified parameters for the SMLS-PSO and 8 advanced PSO variants on each of the 30 CEC2017 test problems. Population size is 80, problem dimension is 30, run times are 31, and MaxFEs are 300,000. Table 6 displays the experimental outcomes. The best experimental result out of the nine algorithms is denoted in boldface in Table 7. The outcomes of the Wilcoxon rank sum test are shown in Table 8. The Friedman test rankings of all compared algorithms for 30D tasks are displayed in Table 9.

The most effective solutions are provided by SMLS-PSO for F1 and F3 functions. To put it another way, the SMLS-PSO method performs exceptionally well for unimodal functions. SDPSO ranks first only on function F4 for basic multimodal functions (F4~F10). Among the six test functions, HCLDMS-PSO achieves the best performance on four multimodal functions, namely F5, F7, F8, and F9. The best results on F6 and F10 are obtained using TSLPSO and EPSO, respectively. SMLS-PSO demonstrates superior performance across all hybrid functions (F11~F20), with the exception of F16 and F17, for which it also offers the second-best solutions. The finest solutions for the F16 and F17 functions are from HCLDMS-PSO.

For the composition functions (F21~F30), SMLS-PSO achieves the optimal solutions on F25, F27, and F30, while also delivering the second-best solutions on F22. Among the hybrid functions (F21 to F24 and F29), which account for five out of the ten test functions, HCLDMS-PSO demonstrates the best performance. The best performance on F26 is provided by EPSO. F28 is the best for GL-PSO. In the last row of Table 9, the count of (Best/2nd Best/Worst) results is summarized for each algorithm. Overall, the proposed SMLS-PSO method outperforms previous PSO algorithms on 14 out of 29 benchmark issues, with SMLS-PSO finishing first and HCLDMS-PSO coming in second. With average rankings of 4.31 and 4.38, respectively, HCLPSO and EPSO achieved a fairly similar result.

Moreover, the SMLS-PSO algorithm demonstrates superior performance compared to eight other prominent PSO variants on most of the 30D CEC2017 benchmark problems. Specifically, on functions F1, F3, F4, F12 to F15, F18 to F20, F25, and F30, SMLS-PSO generates significantly better solutions than all other compared PSO variants, as evidenced by the Wilcoxon rank sum test results in Table 8. On the other hand, the Friedman test p-value of 1.0960 × 10^−17^ in the last row of Table 9 from the Friedman ranks of all examined algorithms clearly suggests that there are substantial differences among the 9 algorithms. With an average rank of 3.15 in solving the benchmark functions for the 30D CEC2017, it can be seen that SMLS-PSO is the PSO version that performs the best in the comparison.

**Figure 5:**
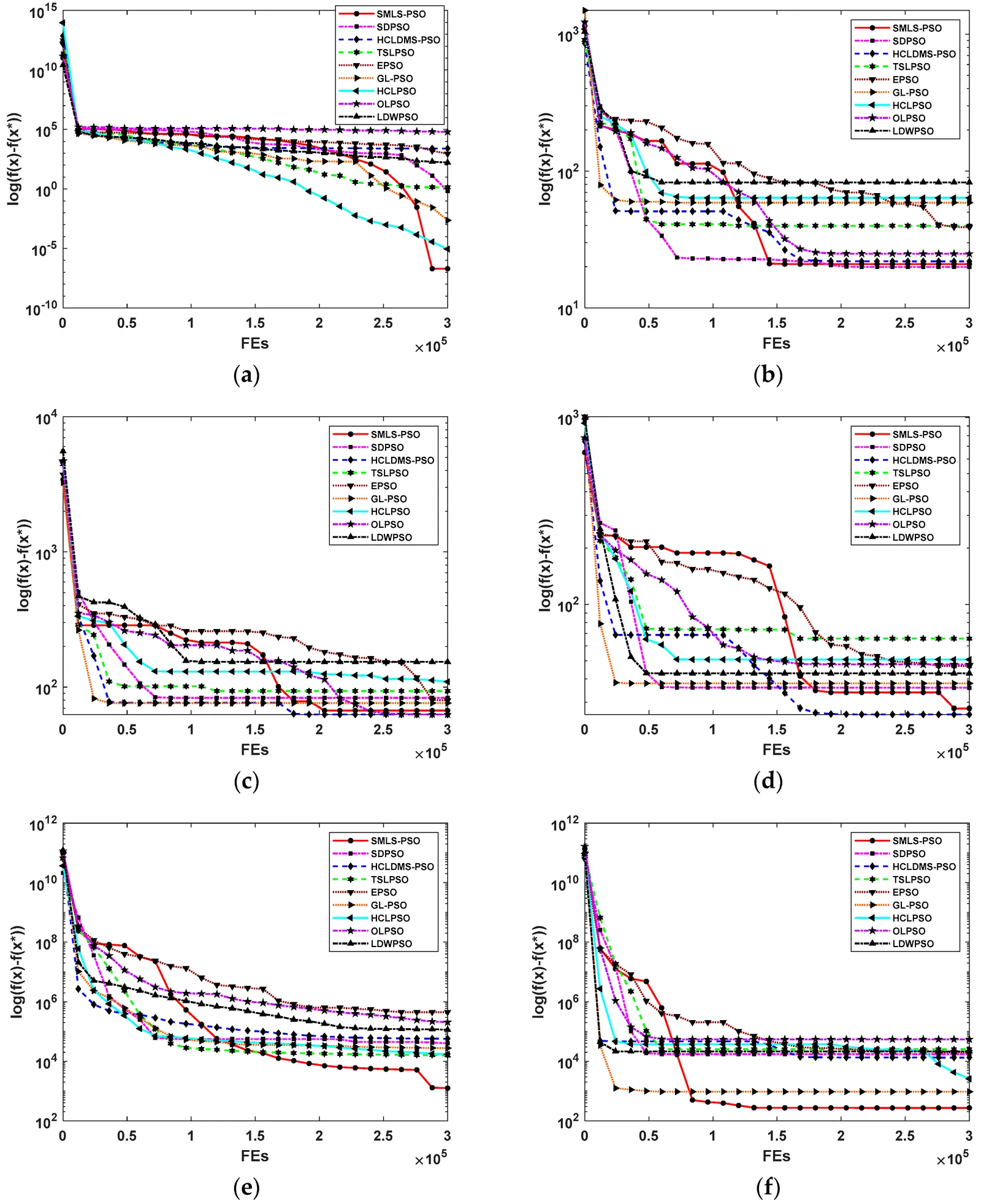
Median convergence graphs of six 30D CEC2017 test suite functions. (**a**) F3 function; (**b**) F5 function; (**c**) F7 function; (**d**) F8 function; (**e**) F12 function; (**f**) F13 function.

**Figure 6:**
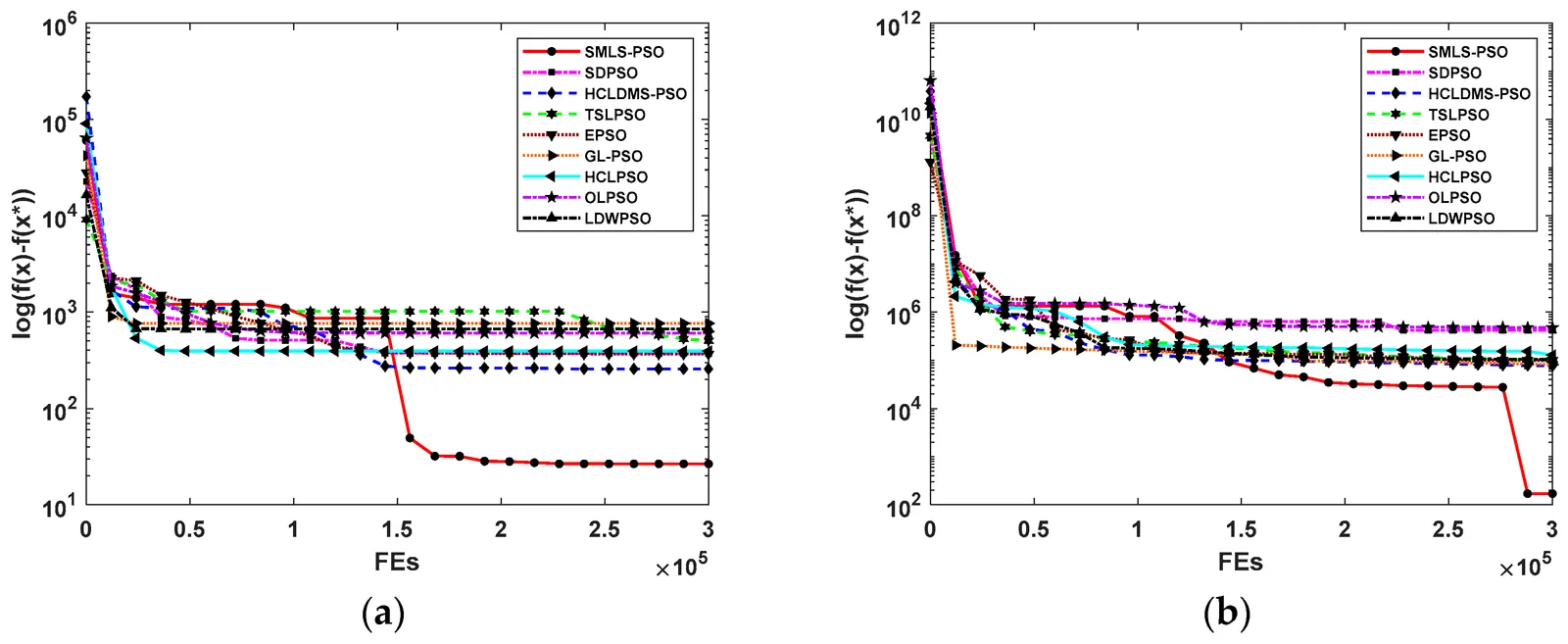

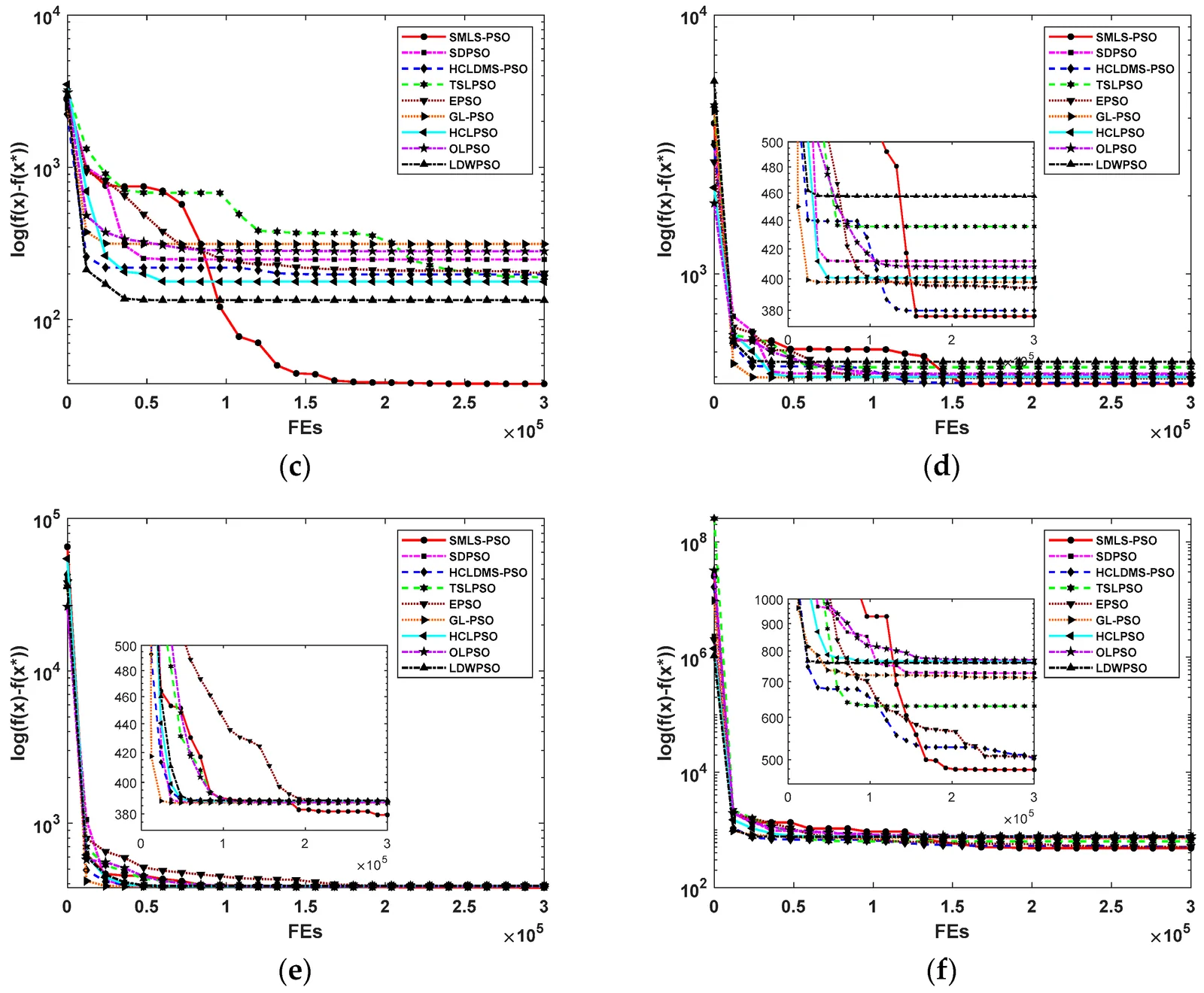
Median convergence graphs of six 30D CEC2017 test suite functions. (**a**) F16 function; (**b**) F18 function; (**c**) F20 function; (**d**) F23 function; (**e**) F25 function; (**f**) F29 function.

To evaluate the convergence performance, several median convergence graphs from 31 runs of the 30D problems are employed. The convergence characteristic graphs of SMLS-PSO and eight other advanced PSO variants for some CEC2017 benchmark functions are presented in Figure 5 and Figure 6. The functions encompass one unimodal function (F3), three multimodal functions (F5, F7, F8), five hybrid functions (F12, F13, F16, F18, F20), and three composition functions (F23, F25, F29).

Figure 5 and Figure 6 show that SMLS-PSO’s initial convergence speed for these functions is not very high, but that after a learning time, once the global optimal basin is discovered, the convergence pace of SMLS-PSO quickly rises and becomes comparable to that of other PSO algorithms. For F3, F12, F13, F18, F20, and F25 functions, where SMLS-PSO performs best, this tendency is considerably more obvious. That means the cooperation of different learning strategies in SMLS-PSO works quite well; that is, SMLS-PSO is able to adaptively tune the probabilities of using different learning strategies to suit different problems and different optimization stages. In addition, the Quasi-Newton local search operator will be fully utilized to enhance exploitation capabilities at the later stage of evolution, as demonstrated in the F3, F8, F12, and F18. In summary, the analysis of convergence performance indicates that the proposed SMLS-PSO achieves the best performance in the majority of 30D problems while also maintaining a competitive convergence rate.

#### 5.5.2. Results Analysis for 50D Problems

The population size, run times, and MaxFEs parameter are all uniformly set to 80, 51, and 500,000 for the 50D CEC2017 test suite functions. In Table 10, the experimental findings are displayed. Table 11 and Table 12 exhibit the findings of the Friedman test and the Wilcoxon rank sum test, respectively. The convergence diagrams are omitted in this section, as they closely mirror those of 30D problems. It is evident that SMLS-PSO obtains the highest performance for F1, F3, and F4. Among the multimodal functions F5, F7, F8, and F9, the optimal results are achieved by the HCLDMS-PSO algorithm. TSLPSO performs best on F6. EPSO offers the top F10 solutions. SMLS-PSO produces exceptional results on F12~F14, F18, and F19, or 5 out of 10 test issues, for hybrid functions F11–F20. F16, F17, and F20 offer the optimum performance for HCLDMS-PSO. OLPSO and EPSO demonstrate the highest performance on functions F11 and F15, respectively. For the composite functions F21 to F30, SMLS-PSO achieves the best results on F25 and F27 to F30, accounting for five out of the ten test problems. Meanwhile, HCLDMS-PSO delivers the best solutions on F21, F23, and F24. EPSO also shows strong performance by obtaining the best solutions on F22 and F26. In terms of average ranks, SMLS-PSO has the optimal mean rank of 2.52, followed closely by HCLDMS-PSO with a mean rank of 2.72, as indicated in Table 10. This outcome is comparable to the 30D test issues in that neither has the worst possible solution.

As evidenced by the Wilcoxon rank sum test results in Table 11, SMLS-PSO significantly surpasses other cutting-edge PSO variants on most of the 50-dimensional CEC2017 benchmark functions. Additionally, Table 12 displays the 50D problem-solving performance rankings of all 9 analyzed algorithms. The Friedman test’s *p*-value, which is 6.7239 × 10^−18^ and is reported in the last row of Table 12, strongly suggests that there are substantial differences between the nine algorithms. With a rank of 2.82, SMLS-PSO is the comparison’s top-performing PSO variant.

#### 5.5.3. Compared 9 PSO Variants Computation Time

This subsection presents the comparative results of the average computational times for nine different PSO variants. The detail computation consume time during every run were records for each CEC2017 problem. The average computation time (Unit: second) of each algorithm in CEC2017 30D and 50D is listed in Table 13. Of the compared methods, GL-PSO and OLPSO require the least amount of calculation time. SMLS-PSO has a higher computational cost than other algorithms, but it is less expensive than the other two self-adaptive PSO variations (SDPSO and EPSO) and HCLDMS-PSO on 30D and 50D. The CL strategy significantly enhances the search performance of SDPSO, HCLDMS-PSO, TSLPSO, EPSO, and HCLPSO, albeit at the expense of increased computational time. In contrast to EPSO and SDPSO, which allocate a dedicated subpopulation for the CL strategy to maintain diversity, SMLS-PSO is more straightforward. It consists of only a single swarm, and its computational time is primarily influenced by the frequency of the CL strategy’s application. Overall, given the excellent performance of the proposed SMLS-PSO, its computational time is deemed acceptable and competitive.

### 5.6. UWB Anchor Layout Optimization and Kinematic Positioning Experimental Results and Analysis

**Figure 7:**
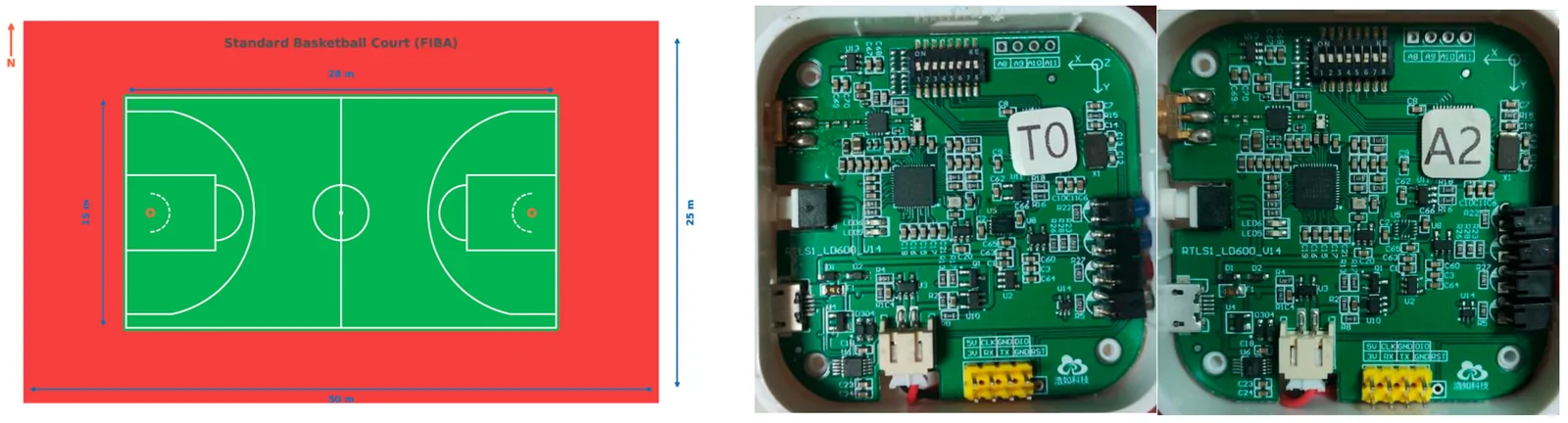
Schematic plan of basketball court (**left**) and physical photos of UWB tags and anchors (**right**).

To compare and analyze the UWB positioning accuracy under different layout schemes and verify the optimization performance of the SMLS-PSO algorithm for UWB anchor layout, UWB dynamic positioning experiments were carried out on a real-world basketball court with dimensions of 25 m × 50 m, as shown in Figure 7. The HR-RTLS ULM1 series modules equipped with the Decawave DWM1000 chip were adopted in the test. The system consists of four anchor base stations (A0~A3) and one mobile tag (T0), and the stable and effective ranging coverage radius of each anchor is 30 m. The basketball court experiment forms a two-stage verification workflow. First, offline layout optimization under ideal ranging conditions quantifies the blind zone defects of the conventional rectangular and diamond layout and the full-space coverage advantage of the SMLS-PSO optimized scheme; second, real dynamic positioning tests under fluctuating ranging and NLOS occlusion (i.e., non-occlusion, metal occlusion, and human-body occlusion) further verify the practical performance gap between the two layouts.

#### 5.6.1. UWB Anchor Offline Layout Optimization Results and Analysis

We set the length of the grid to l=0.5m, and all anchors can be deployed in the range of 3 m near the wall. In this experiment, the outcomes of the rectangular layout, diamond layout, random layout scheme, and 9 state-of-the-art PSO variants in the Section 5.5 optimization layout are compared and analyzed. The weight coefficients of the fitness function are set as $\omega_{1}=0.7$ and $\omega_{2}=0.3$ in this experiment scenario. The parameter settings of the 9 PSO variants algorithm are consistent, where the population size is 80 and MaxFEs is 50,000.

**Figure 8:**
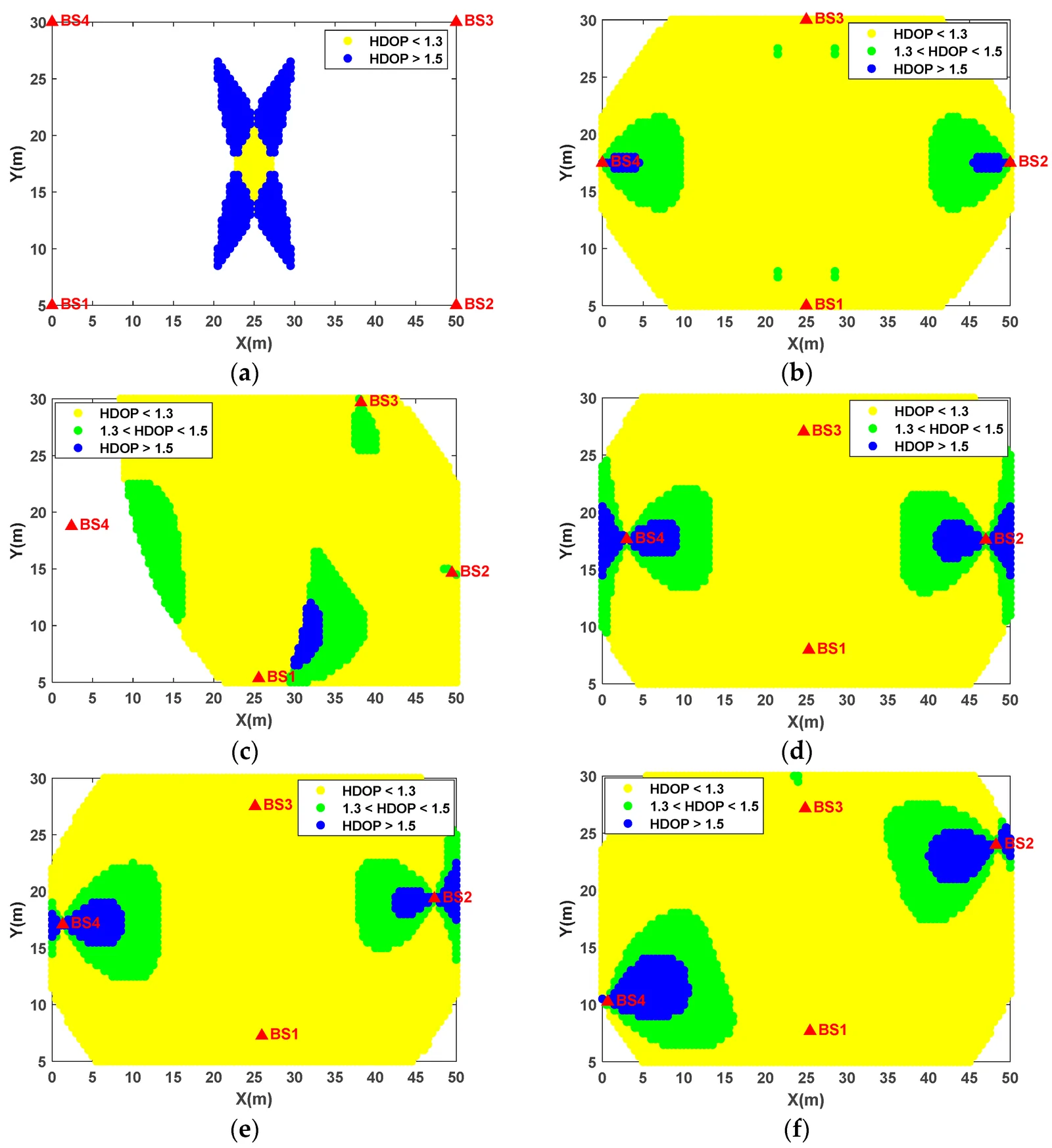

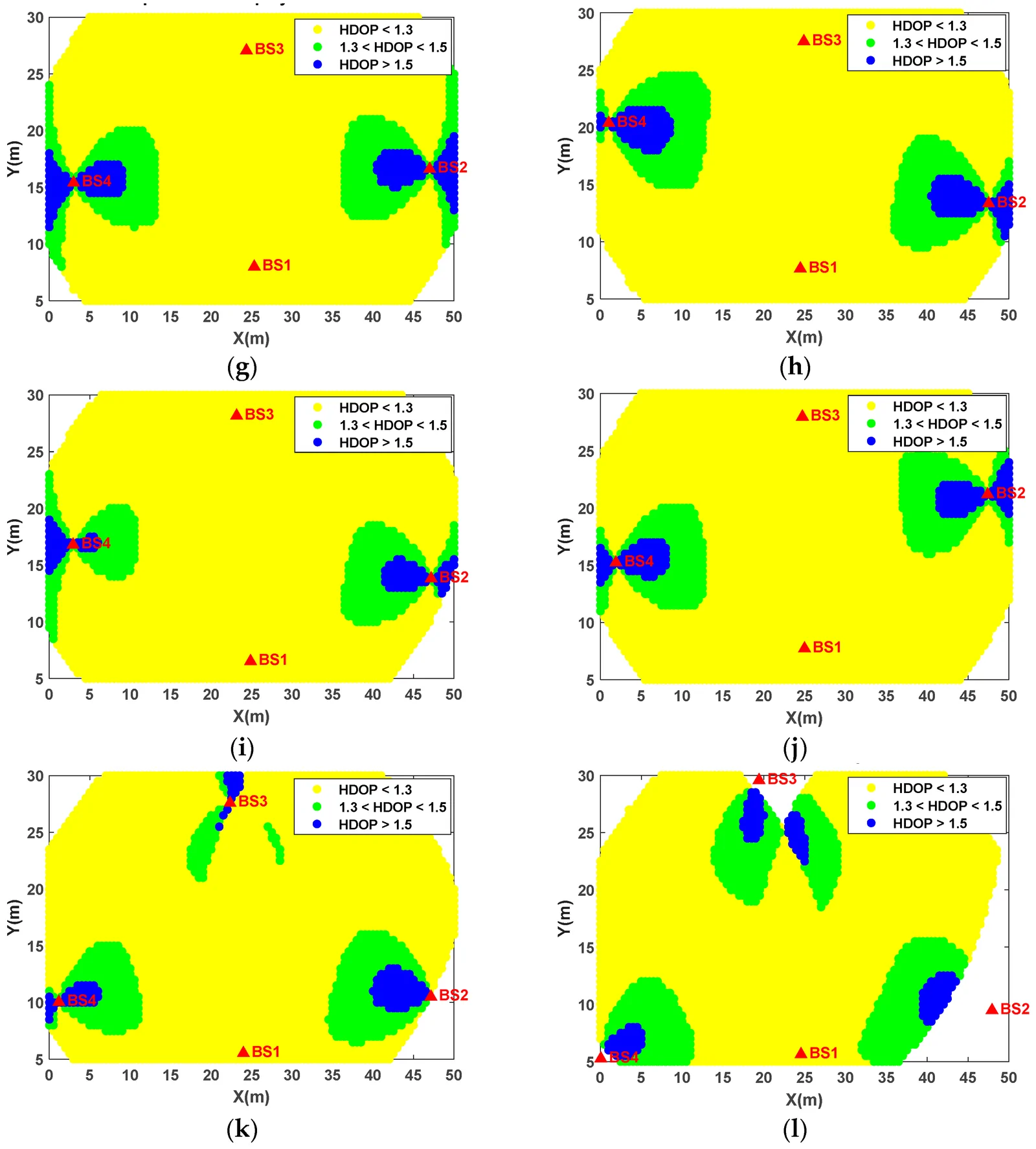
Comparison of the locatable space coverage areas and HDOP value distribution of 9 different PSO variants optimization layout models, where yellow areas indicate HDOP values less than 1.3, green area indicates that the HDOP value is between 1.3 and 1.5, blue area indicates that the HDOP value is greater than 1.5, and white areas represent blind areas that cannot be located. (**a**) rectangular layout; (**b**) diamond layout; (**c**) random layout; (**d**) SMLS-PSO optimization layout; (**e**) SDPSO optimization layout; (**f**) HCLDMS-PSO optimization layout; (**g**) TSLPSO optimization layout; (**h**) EPSO optimization layout; (**i**) GL-PSO optimization layout; (**j**) HCLPSO optimization layout; (**k**) OLPSO optimization layout; (**l**) PSO optimization layout.

Figure 8 illustrates the spatial distribution of HDOP values across the above 12 layout schemes. Red triangles denote the locations of anchors, while blank areas indicate blind zones where positioning is not feasible. The HDOP values at the center points of various spatial grids are represented by yellow, blue, and green. Table 14 presents the best fitness function value, the locatable space coverage rate, and average HDOP values for the above 12 layout schemes. The table includes the mean, standard deviation (Std), rank, and Wilcoxon rank sum test results over repeated runs.

As depicted in Figure 8 and detailed in Table 14, the rectangular layout exhibits the largest localization blind area, only achieving a 7.62% locatable space coverage rate, and the average HDOP value is 1.5292. The diamond layout and random layout achieved 89.12% and 73.44% locatable space coverage rates, respectively. The average HDOP for the locatable area is near 1.2. In 9 state-of-the-art PSO variants, SMLS-PSO obtains the best fitness value of 1.0900. It outperforms TSLPSO and EPSO, which rank second and third, respectively. The SMLS-PSO optimization anchors layout scheme achieved the highest locatable space coverage rate of 95.69% and an average HDOP value of 1.1950. Apart from the original LDWPSO, the other 8 advanced PSO variants all achieved a locatable space coverage rate of more than 91%, and the average HDOP value is approximately 1.2. The Wilcoxon rank sum test results (indicated by “+”) demonstrate that SMLS-PSO significantly outperforms the other 8 comparative algorithms. The relatively low standard deviation of SMLS-PSO indicates that our algorithm produces highly stable and reliable optimization results for UWB anchor layout.

**Figure 9:**
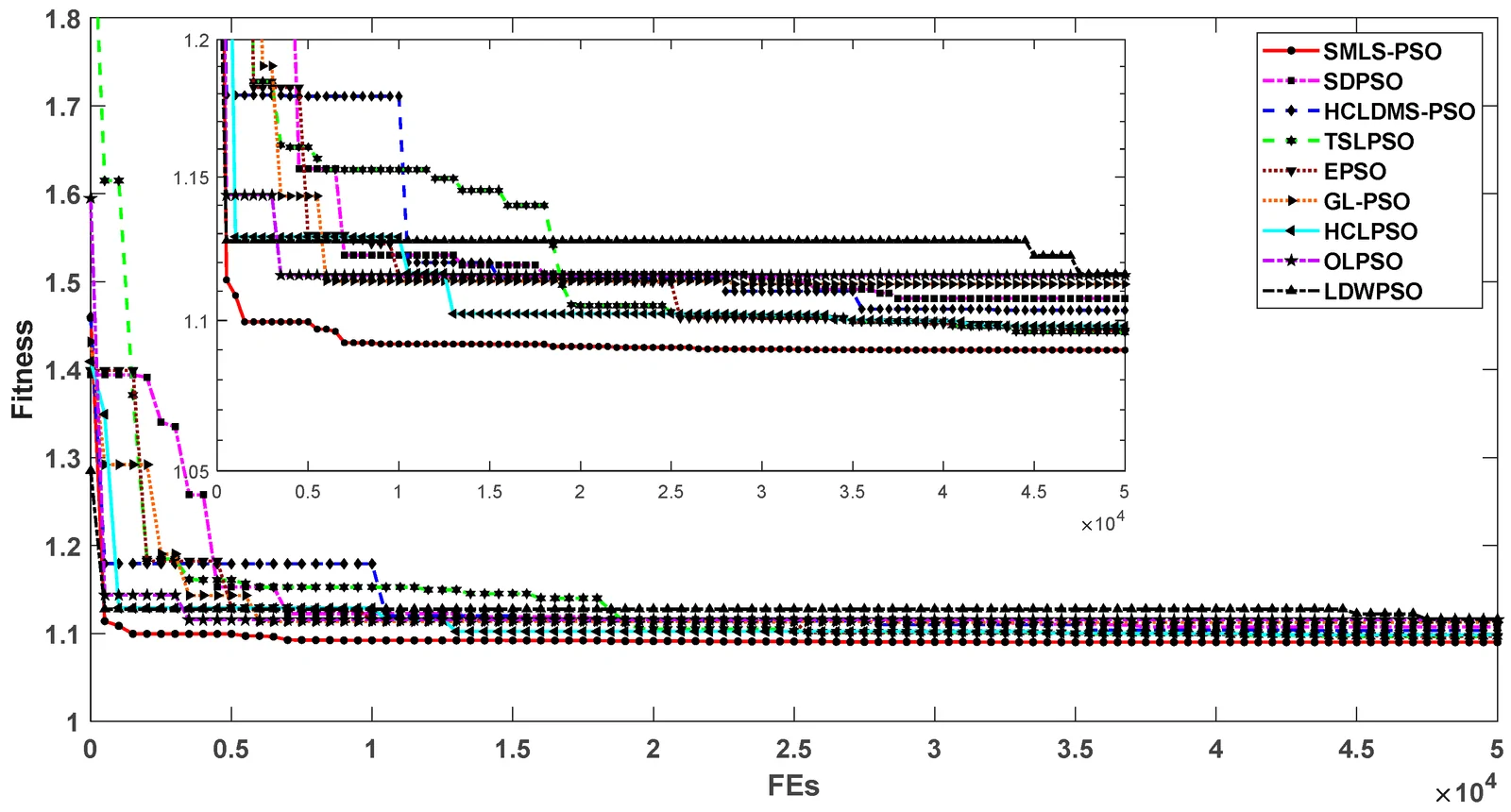
Comparison of the convergence curves of the fitness function values for the UWB anchor node layout optimized by 9 different state-of-the-art PSO variant algorithms.

Figure 9 compares the convergence curves of nine state-of-the-art PSO variants for UWB anchor offline layout optimization in the basketball court experiment. As shown in the plot, SMLS-PSO achieves the lowest fitness value and shows the fastest convergence rate across the whole evolutionary process. It sustains reasonable population diversity to support global exploration, and presents highly stable performance in the late optimization phase. By contrast, several competing PSO variants, including HCLDMS-PSO and OLPSO, drop quickly in the early iterations, yet they fall into premature convergence afterward. The magnified subplot clearly reveals obvious fitness stagnation and oscillations for these algorithms. Owing to its adaptive selection probability and boundary-mapping strategies, SMLS-PSO strikes a favorable balance between global exploration and local exploitation. It obtains the best final fitness with a relatively smooth convergence trajectory.

#### 5.6.2. UWB Kinematic Positioning Results and Analysis

**Figure 10:**
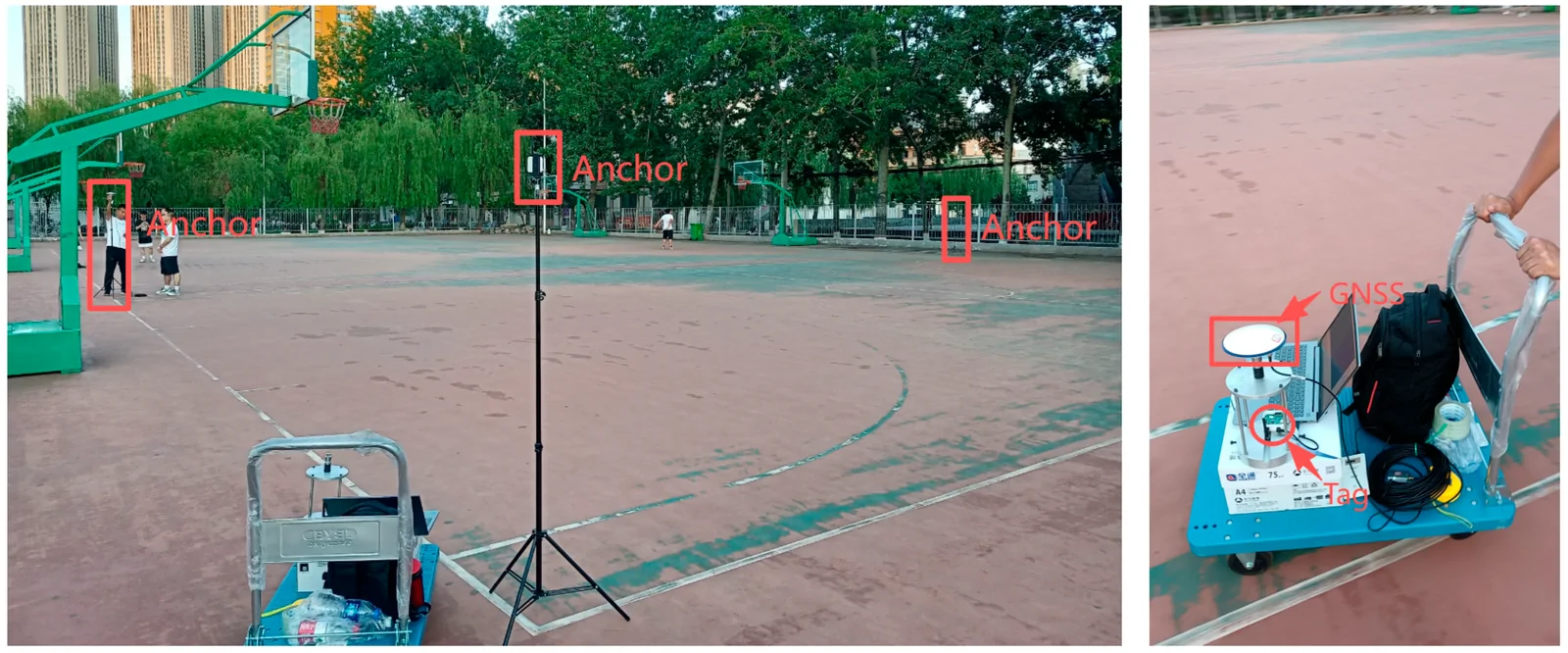
Site photograph of the dynamic trolley experiment on the basketball court.

To compare and verify the practical positioning performance of different UWB anchor layout schemes, two groups of UWB dynamic positioning experiments were conducted on a basketball court. This group experiment share identical court size, anchor quantity, and deployment boundary constraints with Section 5.6.1; the only difference lies in whether ideal fixed ranging or real fluctuating ranging with NLOS interference is adopted. The conventional rectangular layout and the SMLS-PSO optimized layout, which show significant differences in locatable space coverage rate described in Section 5.6.1, were adopted in the tests. Three working conditions, including non-occlusion, metal occlusion, and human body occlusion, were set, and the experimental scenario is shown in Figure 10. The sampling frequency of UWB was uniformly set to 10 Hz. GNSS RTK positioning results with accuracy better than 2 cm were taken as the reference ground truth for statistical analysis. Let $\left(x_{i},y_{i}\right)$ denote the coordinate estimated by UWB, $\left({\hat{x}}_{i},{\hat{y}}_{i}\right)$ denote the ground truth coordinate from GNSS RTK, where i=1,2,…,N, and N is the total number of valid positioning epochs.

The definitions of mean error (Mean) and root mean square error (RMS) are expressed as follows:

(22) $$\left\{\begin{array}{l} Mean=\frac{1}{N}\sum_{i=1}^{N}\sqrt{{\left(x_{i}-{\hat{x}}_{i}\right)}^{2}+{\left(y_{i}-{\hat{y}}_{i}\right)}^{2}}\\ RMS=\sqrt{\frac{1}{N}\sum_{i=1}^{N}\left[{\left(x_{i}-{\hat{x}}_{i}\right)}^{2}+{\left(y_{i}-{\hat{y}}_{i}\right)}^{2}\right]} \end{array}\right.$$

**Figure 11:**
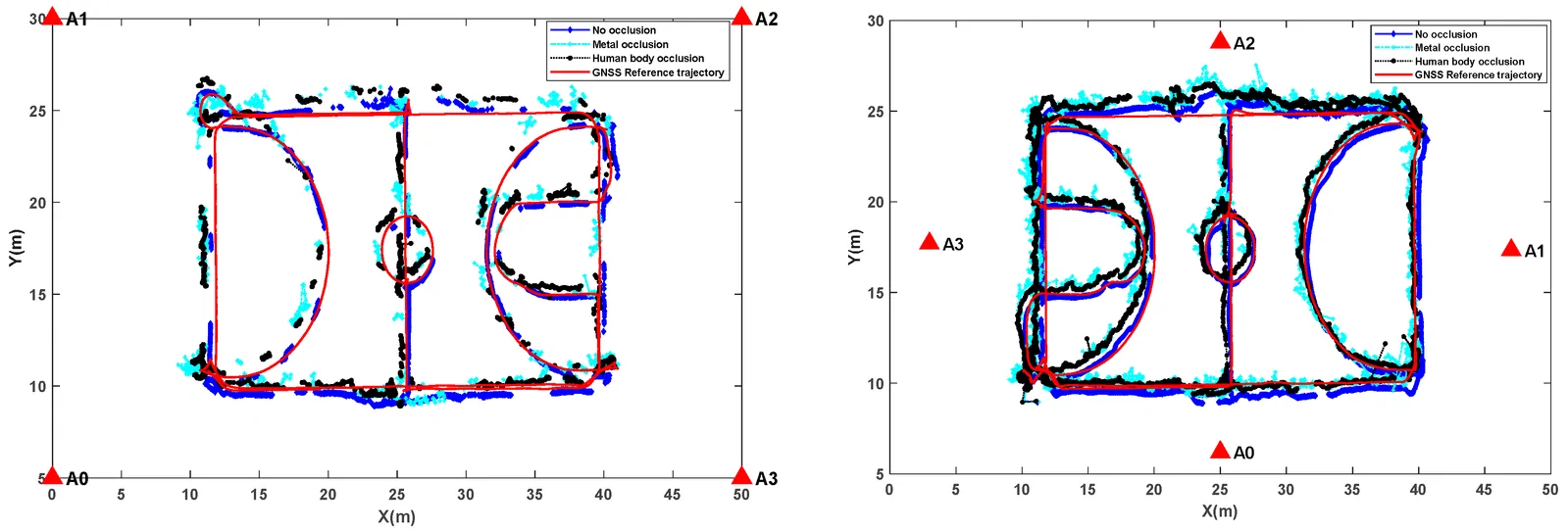
Comparison of dynamic positioning trajectories for rectangular layout and SMLS-PSO optimized layout.

Figure 11 shows the positioning trajectories of the dynamic trolley test under two UWB anchor layouts, and Table 15 summarizes the number and proportion of valid positioning epochs under different occlusion conditions. It can be observed that the proportion of valid positioning epochs for the conventional rectangular anchor layout is generally low: it reaches only 41.31% under non-occlusion, drops to 21.39% under metal occlusion, and is 29.57% under human body occlusion. Affected by its geometric configuration, the rectangular layout is prone to ranging information loss, so a positioning solution cannot be obtained for a large number of epochs, which leads to a remarkable decline in positioning availability. By adopting the SMLS-PSO optimized anchor layout, the localizable capability of the system is significantly improved. The proportion of valid epochs reaches 94.06% in the non-occlusion scenario; even under occlusion interference, it remains at 81.35% and 91.11% for metal occlusion and human body occlusion, respectively. The experimental results verify the effectiveness of the SMLS-PSO optimized layout in improving positioning availability. Comparing the two occlusion conditions, metal occlusion causes more severe degradation of UWB ranging performance and positioning availability than human body occlusion.

Although actual occlusion and ranging shrinkage cause deviations between theoretical coverage in Section 5.6.1 and on-site valid epoch proportions in this section, the SMLS-PSO layout still achieves far higher continuous tracking availability, which provides direct real-world evidence for the effectiveness of the proposed anchor layout optimization method.

**Figure 12:**
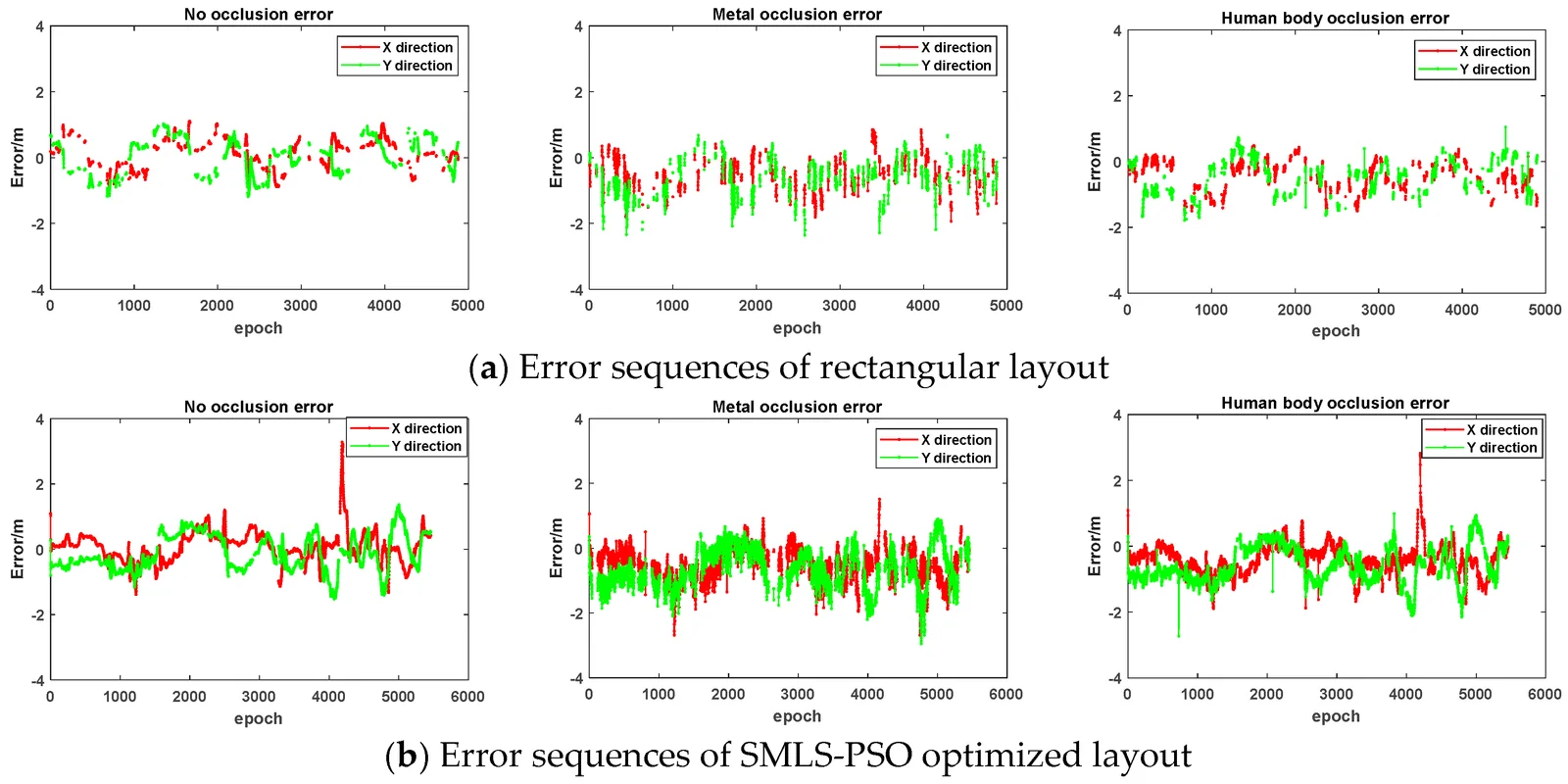
Dynamic positioning error sequences of two layouts under different working conditions.

Table 16 summarizes the Mean and RMS of positioning for the two UWB anchor layouts under non-occlusion, metal occlusion, and human body occlusion in the dynamic trolley experiment, and the corresponding error sequences are illustrated in Figure 12. The results show that the positioning errors of the rectangular layout and the SMLS-PSO optimized layout are generally at the same magnitude. Under the non-occlusion condition, both layouts achieve comparable positioning performance. The Mean and RMS of the rectangular layout are 0.660 m and 0.704 m, respectively, while those of the SMLS-PSO optimized layout are 0.659 m and 0.725 m, with stable fluctuations in error sequences. Under occlusion interference, the positioning accuracy of both layouts degrades, and metal occlusion induces more severe error deterioration than human body occlusion. For metal occlusion, the rectangular layout yields Mean = 0.976 m and RMS = 1.085 m, and the SMLS-PSO optimized layout yields Mean = 1.018 m and RMS = 1.134 m. For human body occlusion, the Mean and RMS are 0.799 m and 0.892 m for the rectangular layout, and 0.897 m and 0.998 m for the SMLS-PSO optimized layout.

### 5.7. Discussions

The comparisons and experiments discussed above demonstrate the robust performance of the proposed SMLS-PSO algorithm across a diverse range of problem types, including unimodal, multimodal, hybrid, and composite functions. The algorithm exhibits notable strengths in terms of convergence accuracy, convergence rate, and overall reliability. SMLS-PSO comprehensively chooses four frequent learning strategies as sub-strategies, in contrast to existing dynamic strategy PSO variations, and a unique self-adaptive learning strategy selection mechanism is created to dynamically utilize their own potential at various stages of evolution. The search behavior and experimental findings using 13 basic benchmark functions showed that the proposed self-adaptive selection mechanism may adaptively modify the probabilities for various issue functions depending on the fitness information payout or feedback at various evolution phases. According to experimental findings on the 30D and 50D CEC2017 test suite, the SMLS-PSO consistently outperforms the other 8 advanced PSO variants, yielding either the best or second-best results on the majority of the test problems. However, SMLS-PSO does not perform well on some functions, such as F6, F10, F21, and F22. This may be explained through the “no free lunch” theorem [44]. According to this theorem, it is impossible for any single algorithm to outperform all others across every type of problem.

Based on the observed convergence graphs, SMLS-PSO maintains population diversity during the initial evolutionary search phase to avoid premature convergence. However, once the global optimal region is identified, the convergence rate rapidly increases. According to the computation cost, SMLS-PSO uses less time when compared to three other great ensemble PSO versions, including SDPSO, HCLDMS-PSO, and EPSO, as well as when compared to TSLPSO, HCLPSO, and LDWPSO. SMLS-PSO was successfully applied into UWB anchors layout optimization problem and achieved the best locatable space coverage rate and average HDOP value. Overall, SMLS-PSO is not only effective for a wide range of benchmark functions but also well-suited to address real-world engineering problems.

## 6. Conclusions

In this paper, a self-adaptive multi-learning strategy PSO (SMLS-PSO) is proposed in which four different characteristics learning strategies (SL-PSO, CLPSO, GOPSO, and DLPSO) constitute the behavior pool. Then, a novel self-adaptive selection mechanism is constructed to gradually determine the suitable learning strategy to update each particle’s velocity and position according to the fitness information payoff or feedback at different evolution stages. In addition, some improvement strategies were introduced to further improve the performance of SMLS-PSO, such as the stagnated counter parameter in GOPSO to reduce the waste of fitness evaluation, a boundary symmetry mapping method to handle out-of-range search, and a Quasi-Newton local search operator to enhance the exploitative ability. To assess the efficiency and performance of the SMLS-PSO algorithm, a comprehensive comparison was conducted with its component algorithms and several state-of-the-art PSO variants using the CEC 2017 test suite. The results indicate that SMLS-PSO demonstrates significant improvements in convergence accuracy and reliability, outperforming both its component PSO variants and other advanced PSO algorithms. SMLS-PSO is not only effective for a wide range of benchmark functions but also well-suited to address real-world UWB anchors layout engineering problems.

This work is also expected to call for more attention to using self-adaptive learning strategy-based mechanisms in swarm intelligence. Several limitations and future research directions need to be noted regarding the present study. In the future, more novel learning strategies need to be consider to this self-adaptive selection framework. Second, the study of SMLS-PSO for large-scale optimization, or even multi-objective optimization, is still very valuable. Our follow-up studies will also include the application of the proposed SMLS-PSO algorithm to some complex practical engineering problems.

## Figures and Tables

**Table 1:** Comparison of the adaptive strategy-selection mechanisms in SLPSO, EPSO, SDPSO, and the proposed SMLS-PSO.

Aspect	SLPSO (2011)	EPSO (2017)	SDPSO (2022)	SMLS-PSO (This Work)
Strategy pool	CLPSO, PSO-CL-pbest, DbV, EbV	PSO, LIPS, FDR-PSO, HPSO-TVAC, CLPSO-gbest	LDWPSO, UPSO, LIPS, CLPSO	**SL-PSO, CLPSO, GOPSO, DLPSO** (balanced exploration/exploitation)
Payoff calculation	Ranking-based weights: better-fitness particles receive higher weights; accumulated to the strategy that generated them	Success/failure counts: only records whether a strategy improved the pbest; $success\_rate=\frac{successes}{successes+failures}$	Average *pbest* improvement per fitness evaluation: $\Delta F_{h}=\sum \frac{f(pbest_{old})-f(pbest_{new})}{f(pbest_{old})}/FEs_{h}$	Total *pbest* improvement (without division by evaluation count, directly reflects cumulative contribution): $\Delta F_{h}=\sum \frac{f(pbest_{old})-f(pbest_{new})}{f(pbest_{old})}$
Probability update formula (with memory)	$proSTR_{j}=(1-\alpha )proSTR_{j}+\alpha \frac{S_{j}}{G_{s}}$, with inertia (α = 1/6)	$p_{k}=\frac{success\_rate_{k}+0.01}{\sum \left(success\_rate_{k}+0.01\right)}$, no inertia, fully replaced each period	$x_{h}(t+1)=x_{h}(t)+\alpha x_{h}(t)(u_{h}(t)-\bar{u}(t))$, with inertia (α = 0.1)	$proSTR_{j}=(1-r)proSTR_{j}+r\cdot p_{k}$, with inertia (r = 1/6)—balances historical experience and current performance
Learning period (LP)	Gs = 10 generations	*LP* = 50 evaluations	*LP* = 200 evaluations	*LP* = 7 generations (tuned for fast response and stability)
Auxiliary mechanisms & population structure	Single population; no extra mechanism	Two sub-populations (large sub-swarm adaptive, small sub-swarm fixed CLPSO)	Two sub-populations + restart (stage-wise reset) + mutation (minimum probability 0.02)	Single population (lightweight) + stagnation-triggered GOBL to escape local optima (unique to SMLS-PSO)

**Table 2:** The results of 30D benchmark test problems with different *LP* values.

Functions	Criteria	LP = 3	LP = 5	LP = 7	LP = 10	LP = 20	LP = 30	LP = 40	LP = 50
F1(Classical)	mean	6.16 × 10^−34^	1.34 × 10^−33^	**0.00 × 10^+00^**	1.03 × 10^−34^	8.22 × 10^−34^	8.32 × 10^−33^	4.11 × 10^−34^	4.01 × 10^−33^
	std	1.88 × 10^−33^	5.22 × 10^−33^	**0.00 × 10^+00^**	5.63 × 10^−34^	3.96 × 10^−33^	4.01 × 10^−32^	2.25 × 10^−33^	1.54 × 10^−32^
	rank	4	6	**1**	2	5	8	3	7
F2(Classical)	mean	**1.93 × 10^−110^**	4.54 × 10^−109^	1.74 × 10^−89^	2.49 × 10^−107^	2.59 × 10^−92^	3.14 × 10^−93^	4.07 × 10^−86^	1.92 × 10^−86^
	std	**1.05 × 10^−109^**	2.41 × 10^−108^	9.51 × 10^−89^	7.84 × 10^−107^	1.42 × 10^−91^	1.53 × 10^−92^	2.22 × 10^−85^	8.81 × 10^−86^
	rank	**1**	2	6	3	5	4	8	7
F3(Classical)	mean	1.52 × 10^−101^	**3.02 × 10^−111^**	2.82 × 10^−109^	2.12 × 10^−108^	2.56 × 10^−100^	1.30 × 10^−92^	3.10 × 10^−91^	6.50 × 10^−87^
	std	8.31 × 10^−101^	**1.35 × 10^−110^**	1.29 × 10^−108^	9.77 × 10^−108^	1.40 × 10^−99^	5.28 × 10^−92^	1.16 × 10^−90^	2.53 × 10^−86^
	rank	4	**1**	2	3	5	6	7	8
F4(Classical)	mean	3.09 × 10^−124^	**8.84 × 10^−205^**	4.68 × 10^−203^	2.05 × 10^−198^	3.44 × 10^−182^	1.61 × 10^−175^	4.64 × 10^−160^	5.29 × 10^−151^
	std	1.69 × 10^−123^	**0.00 × 10^+00^**	0.00 × 10^+00^	0.00 × 10^+00^	0.00 × 10^+00^	0.00 × 10^+00^	2.54 × 10^−159^	2.03 × 10^−150^
	rank	8	**1**	2	3	4	5	6	7
F5(Classical)	mean	6.07 × 10^−19^	3.86 × 10^−19^	**5.52 × 10^−20^**	1.06 × 10^−19^	3.93 × 10^−13^	2.47 × 10^−11^	2.86 × 10^−10^	1.19 × 10^−12^
	std	1.12 × 10^−18^	8.71 × 10^−19^	**1.63 × 10^−19^**	5.66 × 10^−19^	2.15 × 10^−12^	8.60 × 10^−^11	1.47 × 10^−09^	4.62 × 10^−12^
	rank	4	3	**1**	2	5	7	8	6
F6(Classical)	mean	6.77 × 10^−54^	**2.49 × 10^−61^**	7.51 × 10^−45^	7.44 × 10^−37^	2.83 × 10^−56^	4.13 × 10^−45^	5.10 × 10^−48^	3.02 × 10^−45^
	std	3.71 × 10^−53^	**4.07 × 10^−61^**	4.11 × 10^−44^	4.07 × 10^−36^	7.35 × 10^−56^	2.26 × 10^−44^	1.87 × 10^−47^	1.32 × 10^−44^
	rank	3	**1**	7	8	2	6	4	5
F7(Classical)	mean	4.26 × 10^−06^	9.43 × 10^−07^	7.30 × 10^−06^	4.36 × 10^−09^	2.93 × 10^−07^	9.48 × 10^−07^	6.35 × 10^−03^	**1.73 × 10^−10^**
	std	2.27 × 10^−05^	4.89 × 10^−06^	3.81 × 10^−05^	2.27 × 10^−08^	1.59 × 10^−06^	5.19 × 10^−06^	3.47 × 10^−02^	**3.21 × 10^−10^**
	rank	6	4	7	2	3	5	8	**1**
F8(Classical)	mean	2.38 × 10^−01^	3.57 × 10^−01^	1.86 × 10^−01^	2.44 × 10^−01^	3.11 × 10^−01^	**1.78 × 10^−01^**	2.45 × 10^−01^	3.01 × 10^−01^
	std	3.99 × 10^−01^	4.65 × 10^−01^	3.17 × 10^−01^	3.27 × 10^−01^	3.38 × 10^−01^	**3.00 × 10^−01^**	3.27 × 10^−01^	3.55 × 10^−01^
	rank	3	8	2	4	7	**1**	5	6
F9(Classical)	mean	6.30 × 10^−01^	**3.32 × 10^−01^**	3.98 × 10^−01^	7.97 × 10^−01^	6.30 × 10^−01^	6.64 × 10^−01^	1.06 × 10^+00^	7.09 × 10^−01^
	std	8.85 × 10^−01^	**6.03 × 10^−01^**	7.66 × 10^−01^	1.12 × 10^+00^	9.94 × 10^−01^	9.17 × 10^−01^	1.04 × 10^+00^	8.65 × 10^−01^
	rank	3	**1**	2	7	4	5	8	6
F10(Classical)	mean	**1.72 × 10^+00^**	1.96 × 10^+00^	1.80 × 10^+00^	1.99 × 10^+00^	1.85 × 10^+00^	2.08 × 10^+00^	1.85 × 10^+00^	2.01 × 10^+00^
	std	**6.40 × 10^−01^**	7.26 × 10^−01^	6.67 × 10^−01^	6.96 × 10^−01^	6.56 × 10^−01^	6.51 × 10^−01^	6.60 × 10^−01^	1.09 × 10^+00^
	rank	**1**	5	2	6	3	8	4	7
F11(Classical)	mean	1.97 × 10^+01^	1.56 × 10^+01^	**1.50 × 10^+01^**	1.59 × 10^+01^	2.10 × 10^+01^	2.28 × 10^+01^	1.92 × 10^+01^	2.08 × 10^+01^
	std	1.62 × 10^+01^	1.06 × 10^+01^	**1.22 × 10^+01^**	1.70 × 10^+01^	2.04 × 10^+01^	1.85 × 10^+01^	1.60 × 10^+01^	1.23 × 10^+01^
	rank	5	2	**1**	3	7	8	4	6
F12(Classical)	mean	5.92 × 10^−17^	**0.00 × 10^+00^**	2.32 × 10^−14^	3.29 × 10^−04^	1.96 × 10^−03^	5.41 × 10^−07^	7.38 × 10^−04^	1.30 × 10^−16^
	std	2.33 × 10^−16^	**0.00 × 10^+00^**	1.21 × 10^−13^	1.80 × 10^−03^	8.70 × 10^−03^	2.96 × 10^−06^	4.04 × 10^−03^	6.89 × 10^−16^
	rank	2	**1**	4	6	8	5	7	3
F13(Classical)	mean	**7.22 × 10^−15^**	1.36 × 10^−14^	1.18 × 10^−14^	1.61 × 10^−14^	1.91 × 10^−14^	1.53 × 10^−14^	1.84 × 10^−14^	2.39 × 10^−14^
	std	**2.72 × 10^−15^**	1.77 × 10^−14^	1.23 × 10^−14^	2.87 × 10^−14^	2.19 × 10^−14^	1.06 × 10^−14^	1.90 × 10^−14^	4.32 × 10^−14^
	rank	**1**	3	2	5	7	4	6	8
F1(CEC2017)	mean	2.26 × 10^+03^	1.82 × 10^+03^	2.64 × 10^+03^	2.35 × 10^+03^	3.07 × 10^+03^	2.76 × 10^+03^	2.32 × 10^+03^	**1.39 × 10^+03^**
	std	3.16 × 10^+03^	2.49 × 10^+03^	4.95 × 10^+03^	3.89 × 10^+03^	3.71 × 10^+03^	2.88 × 10^+03^	3.34 × 10^+03^	**1.57 × 10^+03^**
	rank	3	2	6	5	8	7	4	**1**
F3(CEC2017)	mean	9.32 × 10^−01^	1.96 × 10^+00^	5.82 × 10^−01^	**1.26 × 10^−01^**	5.41 × 10^−^01	1.59 × 10^+00^	3.95 × 10^−01^	8.81 × 10^−01^
	std	2.56 × 10^+00^	4.69 × 10^+00^	1.78 × 10^+00^	**2.12 × 10^−01^**	1.03 × 10^+00^	2.50 × 10^+00^	5.19 × 10^−01^	1.95 × 10^+00^
	rank	6	8	4	**1**	3	7	2	5
F4(CEC2017)	mean	8.98 × 10^+01^	8.59 × 10^+01^	8.51 × 10^+01^	9.86 × 10^+01^	1.02 × 10^+02^	**8.48 × 10^+01^**	9.46 × 10^+01^	9.92 × 10^+01^
	std	1.70 × 10^+01^	1.50 × 10^+01^	7.71 × 10^+00^	1.30 × 10^+01^	1.45 × 10^+01^	**1.10 × 10^+01^**	1.25 × 10^+01^	1.39 × 10^+01^
	rank	4	3	2	6	8	**1**	5	7
F5(CEC2017)	mean	4.08 × 10^+01^	4.54 × 10^+01^	4.28 × 10^+01^	**4.00 × 10^+01^**	4.65 × 10^+01^	4.08 × 10^+01^	4.09 × 10^+01^	4.69 × 10^+01^
	std	9.68 × 10^+00^	1.36 × 10^+01^	1.09 × 10^+01^	**1.25 × 10^+01^**	1.20 × 10^+01^	9.80 × 10^+00^	1.25 × 10^+01^	1.01 × 10^+01^
	rank	3	6	5	**1**	7	2	4	8
F6(CEC2017)	mean	5.02 × 10^−03^	1.27 × 10^−02^	2.54 × 10^−02^	1.20 × 10^−02^	2.07 × 10^−02^	3.72 × 10^−02^	1.09 × 10^−02^	**3.20 × 10^−03^**
	std	5.45 × 10^−03^	1.71 × 10^−02^	3.22 × 10^−02^	1.19 × 10^−02^	3.71 × 10^−02^	4.33 × 10^−02^	8.40 × 10^−03^	**4.84 × 10^−03^**
	rank	2	5	7	4	6	8	3	**1**
F7(CEC2017)	mean	8.34 × 10^+01^	**7.91 × 10^+01^**	7.99 × 10^+01^	9.26 × 10^+01^	9.88 × 10^+01^	9.41 × 10^+01^	8.22 × 10^+01^	8.93 × 10^+01^
	std	2.19 × 10^+01^	**1.55 × 10^+01^**	1.59 × 10^+01^	1.50 × 10^+01^	2.25 × 10^+01^	1.61 × 10^+01^	1.42 × 10^+01^	2.03 × 10^+01^
	rank	4	**1**	2	6	8	7	3	5
F8(CEC2017)	mean	**3.97 × 10^+01^**	4.80 × 10^+01^	4.53 × 10^+01^	4.65 × 10^+01^	5.14 × 10^+01^	4.79 × 10^+01^	4.84 × 10^+01^	4.58 × 10^+01^
	std	**1.14 × 10^+01^**	1.52 × 10^+01^	1.32 × 10^+01^	1.30 × 10^+01^	1.58 × 10^+01^	1.85 × 10^+01^	1.69 × 10^+01^	8.90 × 10^+00^
	rank	**1**	6	2	4	8	5	7	3
F9(CEC2017)	mean	**7.50 × 10^+00^**	1.17 × 10^+01^	1.45 × 10^+01^	2.15 × 10^+01^	2.29 × 10^+01^	1.58 × 10^+01^	1.99 × 10^+01^	1.31 × 10^+01^
	std	**4.33 × 10^+00^**	1.15 × 10^+01^	1.69 × 10^+01^	3.05 × 10^+01^	1.71 × 10^+01^	8.61 × 10^+00^	2.23 × 10^+01^	2.10 × 10^+01^
	rank	**1**	2	4	7	8	5	6	3
F10(CEC2017)	mean	3.09 × 10^+03^	3.25 × 10^+03^	2.70 × 10^+03^	2.86 × 10^+03^	**2.69 × 10^+03^**	2.80 × 10^+03^	3.37 × 10^+03^	2.88 × 10^+03^
	std	8.34 × 10^+02^	9.27 × 10^+02^	4.89 × 10^+02^	8.12 × 10^+02^	**4.88 × 10^+02^**	4.32 × 10^+02^	1.04 × 10^+03^	7.47 × 10^+02^
	rank	6	7	2	4	**1**	3	8	5
F11(CEC2017)	mean	4.19 × 10^+01^	5.23 × 10^+01^	5.58 × 10^+01^	3.92 × 10^+01^	4.71 × 10^+01^	**3.48 × 10^+01^**	3.69 × 10^+01^	4.40 × 10^+01^
	std	2.80 × 10^+01^	3.46 × 10^+01^	3.75 × 10^+01^	2.63 × 10^+01^	2.13 × 10^+01^	**2.02 × 10^+01^**	2.08 × 10^+01^	3.05 × 10^+01^
	rank	4	7	8	3	6	**1**	2	5
F12(CEC2017)	mean	2.16 × 10^+04^	2.32 × 10^+04^	2.41 × 10^+04^	2.27 × 10^+04^	2.48 × 10^+04^	1.99 × 10^+04^	2.18 × 10^+04^	**1.05 × 10^+04^**
	std	1.19 × 10^+04^	1.80 × 10^+04^	1.62 × 10^+04^	1.40 × 10^+04^	8.38 × 10^+03^	9.09 × 10^+03^	1.36 × 10^+04^	**5.43 × 10^+03^**
	rank	3	6	7	5	8	2	4	**1**
F13(CEC2017)	mean	1.81 × 10^+04^	1.00 × 10^+04^	1.69 × 10^+04^	1.62 × 10^+04^	1.63 × 10^+04^	**9.17 × 10^+03^**	2.10 × 10^+04^	9.71 × 10^+03^
	std	1.34 × 10^+04^	1.24 × 10^+04^	2.21 × 10^+04^	9.60 × 10^+03^	1.64 × 10^+04^	**1.52 × 10^+04^**	1.88 × 10^+04^	1.09 × 10^+04^
	rank	7	3	6	4	5	**1**	8	2
F14(CEC2017)	mean	6.08 × 10^+03^	4.70 × 10^+03^	**3.13 × 10^+03^**	3.33 × 10^+03^	5.54 × 10^+03^	3.73 × 10^+03^	3.44 × 10^+03^	5.19 × 10^+03^
	std	4.65 × 10^+03^	2.71 × 10^+03^	**2.57 × 10^+03^**	3.19 × 10^+03^	7.05 × 10^+03^	2.33 × 10^+03^	1.65 × 10^+03^	5.00 × 10^+03^
	rank	8	5	**1**	2	7	4	3	6
F15(CEC2017)	mean	1.25 × 10^+04^	8.58 × 10^+03^	9.34 × 10^+03^	1.41 × 10^+04^	1.04 × 10^+04^	**6.69 × 10^+03^**	1.07 × 10^+04^	8.80 × 10^+03^
	std	1.13 × 10^+04^	7.27 × 10^+03^	1.32 × 10^+04^	1.21 × 10^+04^	1.20 × 10^+04^	**7.27 × 10^+03^**	7.02 × 10^+03^	8.61 × 10^+03^
	rank	7	2	4	8	5	**1**	6	3
F16(CEC2017)	mean	5.14 × 10^+02^	2.89 × 10^+02^	4.24 × 10^+02^	4.58 × 10^+02^	**2.61 × 10^+02^**	3.48 × 10^+02^	3.89 × 10^+02^	5.22 × 10^+02^
	std	2.50 × 10^+02^	2.13 × 10^+02^	2.09 × 10^+02^	2.04 × 10^+02^	**2.53 × 10^+02^**	1.85 × 10^+02^	2.40 × 10^+02^	1.85 × 10^+02^
	rank	7	2	5	6	**1**	3	4	8
F17(CEC2017)	mean	1.01 × 10^+02^	1.22 × 10^+02^	1.10 × 10^+02^	8.33 × 10^+01^	9.01 × 10^+01^	1.11 × 10^+02^	**7.68 × 10^+01^**	9.14 × 10^+01^
	std	7.07 × 10^+01^	5.80 × 10^+01^	7.04 × 10^+01^	3.75 × 10^+01^	5.61 × 10^+01^	6.51 × 10^+01^	**4.15 × 10^+01^**	3.18 × 10^+01^
	rank	5	8	6	2	3	7	**1**	4
F18(CEC2017)	mean	1.20 × 10^+05^	1.91 × 10^+05^	1.09 × 10^+05^	**5.29 × 10^+04^**	6.98 × 10^+04^	1.15 × 10^+05^	1.42 × 10^+05^	2.16× 10^+05^
	std	4.57 × 10^+04^	2.69 × 10^+05^	8.30 × 10^+04^	**3.36 × 10^+04^**	4.81 × 10^+04^	1.03 × 10^+05^	7.75 × 10^+04^	1.77 × 10^+05^
	rank	5	7	3	**1**	2	4	6	8
F19(CEC2017)	mean	1.11 × 10^+04^	6.79 × 10^+03^	1.27 × 10^+04^	**6.20 × 10^+03^**	1.14 × 10^+04^	9.91 × 10^+03^	2.12 × 10^+04^	1.25 × 10^+04^
	std	1.52 × 10^+04^	1.20 × 10^+04^	1.18 × 10^+04^	**6.52 × 10^+03^**	1.61 × 10^+04^	1.22 × 10^+04^	1.13 × 10^+04^	1.36 × 10^+04^
	rank	4	2	7	**1**	5	3	8	6
F20(CEC2017)	mean	1.18 × 10^+02^	1.26 × 10^+02^	1.03 × 10^+02^	1.35 × 10^+02^	1.14 × 10^+02^	**5.50 × 10^+01^**	1.22 × 10^+02^	1.26 × 10^+02^
	std	7.87 × 10^+01^	7.38 × 10^+01^	1.11 × 10^+02^	1.05 × 10^+02^	7.54 × 10^+01^	**4.03 × 10^+01^**	8.70 × 10^+01^	9.46 × 10^+01^
	rank	4	7	2	8	3	**1**	5	6
F21(CEC2017)	mean	2.39 × 10^+02^	2.43 × 10^+02^	2.49 × 10^+02^	2.45 × 10^+02^	2.53 × 10^+02^	2.51 × 10^+02^	2.43 × 10^+02^	**2.38 × 10^+02^**
	std	1.02 × 10^+01^	1.03 × 10^+01^	1.49 × 10^+01^	1.14 × 10^+01^	1.19 × 10^+01^	1.36 × 10^+01^	1.30 × 10^+01^	**1.22 × 10^+01^**
	rank	2	3	6	5	8	7	4	**1**
F22(CEC2017)	mean	1.01 × 10^+02^	1.00 × 10^+02^	1.01 × 10^+02^	**1.00 × 10^+02^**	1.00 × 10^+02^	1.00 × 10^+02^	1.01 × 10^+02^	1.00 × 10^+02^
	std	1.47 × 10^+00^	1.17 × 10^+00^	1.46 × 10^+00^	**1.36 × 10^−12^**	1.04 × 10^+00^	1.24 × 10^+00^	1.44 × 10^+00^	7.76 × 10^−01^
	rank	7	3	6	**1**	5	4	8	2
F23(CEC2017)	mean	3.92 × 10^+02^	3.93 × 10^+02^	**3.87 × 10^+02^**	3.99 × 10^+02^	3.91 × 10^+02^	3.94 × 10^+02^	3.91 × 10^+02^	3.95 × 10^+02^
	std	9.27 × 10^+00^	1.46 × 10^+01^	**1.10 × 10^+01^**	1.56 × 10^+01^	6.70 × 10^+00^	1.34 × 10^+01^	8.97 × 10^+00^	1.37 × 10^+01^
	rank	4	5	**1**	8	3	6	2	7
F24(CEC2017)	mean	4.67 × 10^+02^	**4.56 × 10^+02^**	4.60 × 10^+02^	4.61 × 10^+02^	4.71 × 10^+02^	4.66 × 10^+02^	4.61 × 10^+02^	4.71 × 10^+02^
	std	2.73 × 10^+01^	**7.86 × 10^+00^**	1.23 × 10^+01^	7.95 × 10^+00^	1.51 × 10^+01^	1.20 × 10^+01^	1.04 × 10^+01^	1.35 × 10^+01^
	rank	6	**1**	2	3	8	5	4	7
F25(CEC2017)	mean	3.88 × 10^+02^	3.88 × 10^+02^	**3.87 × 10^+02^**	3.87 × 10^+02^	3.88 × 10^+02^	3.88 × 10^+02^	3.88 × 10^+02^	3.88 × 10^+02^
	std	6.56 × 10^−01^	5.12 × 10^−01^	**1.96 × 10^+00^**	1.31 × 10^+00^	7.14 × 10^−01^	8.68 × 10^−01^	1.25 × 10^+00^	8.24 × 10^−01^
	rank	4	5	**1**	2	6	8	3	7
F26(CEC2017)	mean	1.38 × 10^+03^	1.41 × 10^+03^	1.39 × 10^+03^	**1.24 × 10^+03^**	1.39 × 10^+03^	1.54 × 10^+03^	1.34 × 10^+03^	1.56 × 10^+03^
	std	3.99 × 10^+02^	4.26 × 10^+02^	4.37 × 10^+02^	**5.46 × 10^+02^**	4.50 × 10^+02^	1.77 × 10^+02^	3.78 × 10^+02^	1.78 × 10^+02^
	rank	3	6	5	**1**	4	7	2	8
F27(CEC2017)	mean	**5.10 × 10^+02^**	5.12 × 10^+02^	5.13 × 10^+02^	5.18 × 10^+02^	5.15 × 10^+02^	5.16 × 10^+02^	5.17 × 10^+02^	5.17 × 10^+02^
	std	**5.40 × 10^+00^**	5.26 × 10^+00^	6.27 × 10^+00^	6.66 × 10^+00^	6.48 × 10^+00^	6.46 × 10^+00^	8.92 × 10^+00^	5.66 × 10^+00^
	rank	**1**	2	3	8	4	5	7	6
F28(CEC2017)	mean	4.03 × 10^+02^	3.88 × 10^+02^	**3.54 × 10^+02^**	3.94 × 10^+02^	3.65 × 10^+02^	3.67 × 10^+02^	3.97 × 10^+02^	4.31 × 10^+02^
	std	5.80 × 10^+01^	6.71 × 10^+01^	**7.22 × 10^+01^**	6.82 × 10^+01^	6.94 × 10^+01^	7.16 × 10^+01^	5.68 × 10^+01^	3.69 × 10^+01^
	rank	7	4	**1**	5	2	3	6	8
F29(CEC2017)	mean	**5.21 × 10^+02^**	5.57 × 10^+02^	5.42 × 10^+02^	5.85 × 10^+02^	6.15 × 10^+02^	5.78 × 10^+02^	5.42 × 10^+02^	5.73 × 10^+02^
	std	**9.64 × 10^+01^**	8.62 × 10^+01^	1.00 × 10^+02^	9.54 × 10^+01^	1.08 × 10^+02^	8.25 × 10^+01^	5.37 × 10^+01^	5.34 × 10^+01^
	rank	**1**	4	2	7	8	6	3	5
F30(CEC2017)	mean	7.50 × 10^+03^	7.42 × 10^+03^	5.39 × 10^+03^	**5.38 × 10^+03^**	6.71 × 10^+03^	6.72 × 10^+03^	6.83 × 10^+03^	7.16 × 10^+03^
	std	3.20 × 10^+03^	3.59 × 10^+03^	2.52 × 10^+03^	**3.19 × 10^+03^**	3.95 × 10^+03^	3.33 × 10^+03^	3.52 × 10^+03^	3.46 × 10^+03^
	rank	8	7	2	**1**	3	4	5	6
**Average rank**		4.10	3.98	**3.60**	4.12	5.19	4.74	5.02	5.26
**Final rank**		3	2	**1**	4	7	5	6	8
**Best/2nd Best/Worst**		7/3/3	7/8/3	**7/14/1**	7/6/5	2/3/10	6/2/5	1/4/8	5/2/7

**Table 3:** The results of 30D benchmark test problems with different *OBL_limit*.

Functions	*OBL_limit* = 10		*OBL_limit* = 20		*OBL_limit* = 30		*OBL_limit* = 40		*OBL_limit* = 50	
	Mean ± Std	*Rk*	Mean ± Std	*Rk*	Mean ± Std	*Rk*	Mean ± Std	*Rk*	Mean ± Std	*Rk*
F1(Classical)	1.44 × 10^−33^ ± 5.71 × 10^−33^	5	1.03 × 10^−33^ ± 5.63 × 10^−33^	2	1.34 × 10^−33^ ± 5.22 × 10^−33^	4	**0.00 × 10^+00^ ± 0.00 × 10^+00^**	**1**	1.13 × 10^−33^ ± 6.19 × 10^−33^	3
F2(Classical)	2.05 × 10^−59^ ± 1.12 × 10^−58^	5	6.47 × 10^−108^ ± 2.15 × 10^−107^	3	4.54 × 10^−109^ ± 2.41 × 10^−108^	2	**1.14 × 10^−111^ ± 5.68 × 10^−111^**	**1**	2.19 × 10^−60^ ± 1.20 × 10^−59^	4
F3(Classical)	6.36 × 10^−104^ ± 2.34 × 10^−103^	2	5.93 × 10^−91^ ± 3.25 × 10^−90^	3	**3.02 × 10^−111^ ± 1.35 × 10^−110^**	**1**	4.88 × 10^−79^ ± 2.67 × 10^−78^	4	2.85 × 10^−78^ ± 1.56 × 10^−77^	5
F4(Classical)	4.52 × 10^−97^ ± 2.47 × 10^−96^	5	9.47 × 10^−203^ ± 0.00 × 10^+00^	4	8.84 × 10^−205^ ± 0.00 × 10^+00^	3	**2.53 × 10^−207^ ± 0.00 × 10^+00^**	**1**	3.53 × 10^−207^ ± 0.00 × 10^+00^	2
F5(Classical)	3.59 × 10^−12^ ± 1.97 × 10^−11^	5	8.76 × 10^−20^ ± 1.52 × 10^−19^	2	3.86 × 10^−19^ ± 8.71 × 10^−19^	4	**7.26 × 10^−20^ ± 1.61 × 10^−19^**	**1**	1.05 × 10^−19^ ± 3.46 × 10^−19^	3
F6(Classical)	4.05 × 10^−57^ ± 8.18 × 10^−57^	5	1.92 × 10^−59^ ± 6.74 × 10^−59^	3	2.49 × 10^−61^ ± 4.07 × 10^−61^	2	5.79 × 10^−59^ ± 3.17 × 10^−58^	4	**1.15 × 10^−61^ ± 3.28 × 10^−61^**	**1**
F7(Classical)	3.06 × 10^−07^ ± 9.31 × 10^−07^	2	**1.64 × 10^−07^ ± 6.26 × 10^−07^**	**1**	9.43 × 10^−07^ ± 4.89 × 10^−06^	3	3.82 × 10^−03^ ± 2.09 × 10^−02^	5	2.20 × 10^−04^ ± 1.20 × 10^−03^	4
F8(Classical)	3.79 × 10^−01^ ± 3.37 × 10^−01^	5	2.34 × 10^−01^ ± 3.42 × 10^−01^	3	3.57 × 10^−01^ ± 4.65 × 10^−01^	4	**1.11 × 10^−01^ ± 2.53 × 10^−01^**	**1**	2.16 × 10^−01^ ± 3.46 × 10^−01^	2
F9(Classical)	3.65 × 10^−01^ ± 6.65 × 10^−01^	2	3.98 × 10^−01^ ± 7.66 × 10^−01^	3	**3.32 × 10^−01^ ± 6.03 × 10^−01^**	**1**	6.63 × 10^−01^ ± 8.40 × 10^−01^	5	5.97 × 10^−01^ ± 8.10 × 10^−01^	4
F10(Classical)	2.22 × 10^+00^ ± 8.46 × 10^−01^	5	1.84 × 10^+00^ ± 7.75 × 10^−01^	3	1.96 × 10^+00^ ± 7.26 × 10^−01^	4	1.84 × 10^+00^ ± 6.33 × 10^−01^	2	**1.65 × 10^+00^ ± 6.29 × 10^−01^**	**1**
F11(Classical)	2.51 × 10^+01^ ± 2.59 × 10^+01^	5	2.05 × 10^+01^ ± 1.80 × 10^+01^	3	**1.56 × 10^+01^ ± 1.06 × 10^+01^**	**1**	1.65 × 10^+01^ ± 1.43 × 10^+01^	2	2.28 × 10^+01^ ± 1.54 × 10^+01^	4
F12(Classical)	6.57 × 10^−04^ ± 3.60 × 10^−03^	5	1.26 × 10^−16^ ± 6.89 × 10^−16^	2	**0.00 × 10^+00^ ± 0.00 × 10^+00^**	**1**	3.29 × 10^−04^ ± 1.80 × 10^−03^	4	2.47 × 10^−04^ ± 1.35 × 10^−03^	3
F13(Classical)	1.08 × 10^−14^ ± 9.90 × 10^−15^	2	1.37 × 10^−14^ ± 1.83 × 10^−14^	5	1.36 × 10^−14^ ± 1.77 × 10^−14^	4	1.09 × 10^−14^ ± 1.06 × 10^−14^	3	**8.64 × 10^−15^ ± 6.84 × 10^−15^**	**1**
F1(CEC2017)	2.24 × 10^+03^ ± 2.79 × 10^+03^	3	**1.21 × 10^+03^ ± 1.29 × 10^+03^**	**1**	1.82 × 10^+03^ ± 2.24 × 10^+03^	2	3.90 × 10^+03^ ± 4.43 × 10^+03^	5	2.96 × 10^+03^ ± 2.63 × 10^+03^	4
F3(CEC2017)	1.96 × 10^+01^ ± 2.35 × 10^+01^	5	**4.35 × 10^−03^ ± 4.90 × 10^−03^**	**1**	4.49 × 10^−01^ ± 8.01 × 10^−01^	4	6.54 × 10^−02^ ± 1.01 × 10^−01^	2	1.53 × 10^−01^ ± 3.20 × 10^−01^	3
F4(CEC2017)	8.86 × 10^+01^ ± 7.71 × 10^+00^	2	9.07 × 10^+01^ ± 1.92 × 10^+01^	3	9.27 × 10^+01^ ± 1.49 × 10^+01^	4	**8.09 × 10^+01^ ± 3.03 × 10^+01^**	**1**	9.83 × 10^+01^ ± 1.61 × 10^+01^	5
F5(CEC2017)	4.28 × 10^+01^ ± 1.26 × 10^+01^	4	4.24 × 10^+01^ ± 1.19 × 10^+01^	3	3.97 × 10^+01^ ± 1.28 × 10^+01^	2	4.69 × 10^+01^ ± 6.14 × 10^+00^	5	**3.57 × 10^+01^ ± 1.24 × 10^+01^**	**1**
F6(CEC2017)	6.89 × 10^−03^ ± 1.16 × 10^−02^	5	**3.14 × 10^−03^ ± 4.32 × 10^−03^**	**1**	6.19 × 10^−03^ ± 7.19 × 10^−03^	3	6.12 × 10^−03^ ± 5.88 × 10^−03^	2	6.81 × 10^−03^ ± 1.43 × 10^−02^	4
F7(CEC2017)	8.89 × 10^+01^ ± 1.51 × 10^+01^	4	8.24 × 10^+01^ ± 1.49 × 10^+01^	2	8.25 × 10^+01^ ± 1.28 × 10^+01^	3	**7.95 × 10^+01^ ± 1.07 × 10^+01^**	**1**	9.14 × 10^+01^ ± 2.12 × 10^+01^	5
F8(CEC2017)	4.73 × 10^+01^ ± 1.50 × 10^+01^	4	**4.22 × 10^+01^ ± 1.08 × 10^+01^**	**1**	4.63 × 10^+01^ ± 1.76 × 10^+01^	3	4.61 × 10^+01^ ± 1.01 × 10^+01^	2	4.84 × 10^+01^ ± 5.46 × 10^+00^	5
F9(CEC2017)	1.08 × 10^+01^ ± 4.87 × 10^+00^	3	1.08 × 10^+01^ ± 5.74 × 10^+00^	2	1.21 × 10^+01^ ± 8.35 × 10^+00^	4	**7.94 × 10^+00^ ± 7.90 × 10^+00^**	**1**	1.24 × 10^+01^ ± 1.10 × 10^+01^	5
F10(CEC2017)	2.81 × 10^+03^ ± 8.53 × 10^+02^	3	3.61 × 10^+03^ ± 1.51 × 10^+03^	5	**2.80 × 10^+03^ ± 5.73 × 10^+02^**	**1**	2.81 × 10^+03^ ± 5.79 × 10^+02^	2	2.88 × 10^+03^ ± 7.86 × 10^+02^	4
F11(CEC2017)	**3.33 × 10^+01^ ± 2.32 × 10^+01^**	**1**	6.00 × 10^+01^ ± 3.33 × 10^+01^	5	5.26 × 10^+01^ ± 3.20 × 10^+01^	4	3.40 × 10^+01^ ± 2.39 × 10^+01^	2	3.73 × 10^+01^ ± 2.12 × 10^+01^	3
F12(CEC2017)	2.47 × 10^+04^ ± 1.86 × 10^+04^	5	**1.90 × 10^+04^ ± 8.44 × 10^+03^**	**1**	2.29 × 10^+04^ ± 1.84 × 10^+04^	4	2.16 × 10^+04^ ± 1.86 × 10^+04^	3	1.99 × 10^+04^ ± 1.33 × 10^+04^	2
F13(CEC2017)	**1.07 × 10^+04^ ± 1.75 × 10^+04^**	**1**	1.50 × 10^+04^ ± 1.73 × 10^+04^	4	1.47 × 10^+04^ ± 1.30 × 10^+04^	2	2.00 × 10^+04^ ± 1.86 × 10^+04^	5	1.48 × 10^+04^ ± 1.75 × 10^+04^	3
F14(CEC2017)	3.35 × 10^+03^ ± 2.62 × 10^+03^	2	6.50 × 10^+03^ ± 4.68 × 10^+03^	4	4.80 × 10^+03^ ± 3.13 × 10^+03^	3	6.88 × 10^+03^ ± 4.98 × 10^+03^	5	**3.23 × 10^+03^ ± 3.27 × 10^+03^**	**1**
F15(CEC2017)	1.05 × 10^+04^ ± 8.45 × 10^+03^	3	1.23 × 10^+04^ ± 8.89 × 10^+03^	5	**8.88 × 10^+03^ ± 1.02 × 10^+04^**	**1**	1.00 × 10^+04^ ± 8.60 × 10^+03^	2	1.22 × 10^+04^ ± 1.39 × 10^+04^	4
F16(CEC2017)	**3.34 × 10^+02^ ± 1.30 × 10^+02^**	**1**	4.94 × 10^+02^ ± 2.97 × 10^+02^	5	3.55 × 10^+02^ ± 2.27 × 10^+02^	2	4.79 × 10^+02^ ± 1.99 × 10^+02^	4	4.79 × 10^+02^ ± 2.17 × 10^+02^	3
F17(CEC2017)	1.12 × 10^+02^ ± 7.02 × 10^+01^	4	1.15 × 10^+02^ ± 6.09 × 10^+01^	5	**8.05 × 10^+01^ ± 4.19 × 10^+01^**	**1**	8.11 × 10^+01^ ± 4.65 × 10^+01^	2	1.08 × 10^+02^ ± 9.25 × 10^+01^	3
F18(CEC2017)	1.05 × 10^+05^ ± 8.14 × 10^+04^	2	1.61 × 10^+05^ ± 1.05 × 10^+05^	4	2.35 × 10^+05^ ± 2.01 × 10^+05^	5	1.53 × 10^+05^ ± 1.19 × 10^+05^	3	**5.55 × 10^+04^ ± 4.12 × 10^+04^**	**1**
F19(CEC2017)	7.42 × 10^+03^ ± 1.15 × 10^+04^	2	**5.28 × 10^+03^ ± 4.68 × 10^+03^**	**1**	1.83 × 10^+04^ ± 1.72 × 10^+04^	5	1.21 × 10^+04^ ± 1.26 × 10^+04^	4	1.05 × 10^+04^ ± 1.50 × 10^+04^	3
F20(CEC2017)	1.33 × 10^+02^ ± 1.03 × 10^+02^	4	1.17 × 10^+02^ ± 7.75 × 10^+01^	3	1.46 × 10^+02^ ± 7.26 × 10^+01^	5	**7.18 × 10^+01^ ± 5.09 × 10^+01^**	**1**	1.05 × 10^+02^ ± 9.14 × 10^+01^	2
F21(CEC2017)	2.43 × 10^+02^ ± 1.11 × 10^+01^	4	**2.36 × 10^+02^ ± 1.12 × 10^+01^**	**1**	2.42 × 10^+02^ ± 1.40 × 10^+01^	2	2.46 × 10^+02^ ± 9.72 × 10^+00^	5	2.43 × 10^+02^ ± 1.31 × 10^+01^	3
F22(CEC2017)	1.00 × 10^+02^ ± 7.05 × 10^−12^	3	1.01 × 10^+02^ ± 1.54 × 10^+00^	5	1.00 × 10^+02^ ± 2.25 × 10^−12^	2	1.00 × 10^+02^ ± 7.78 × 10^−01^	4	**1.00 × 10^+02^ ± 1.84 × 10^−12^**	**1**
F23(CEC2017)	**3.85 × 10^+02^ ± 5.98 × 10^+00^**	**1**	3.93 × 10^+02^ ± 1.30 × 10^+01^	3	3.86 × 10^+02^ ± 1.12 × 10^+01^	2	3.94 × 10^+02^ ± 1.24 × 10^+01^	5	3.93 × 10^+02^ ± 1.39 × 10^+01^	4
F24(CEC2017)	4.65 × 10^+02^ ± 1.24 × 10^+01^	5	4.65 × 10^+02^ ± 1.17 × 10^+01^	4	**4.59 × 10^+02^ ± 1.85 × 10^+01^**	**1**	4.62 × 10^+02^ ± 1.07 × 10^+01^	3	4.62 × 10^+02^ ± 1.22 × 10^+01^	2
F25(CEC2017)	**3.88 × 10^+02^ ± 6.54 × 10^−01^**	**1**	3.88 × 10^+02^ ± 1.43 × 10^+00^	2	3.88 × 10^+02^ ± 7.75 × 10^−01^	4	3.88 × 10^+02^ ± 7.15 × 10^−01^	3	3.88 × 10^+02^ ± 9.54 × 10^−01^	5
F26(CEC2017)	**1.32 × 10^+03^ ± 4.45 × 10^+02^**	**1**	1.50 × 10^+03^ ± 9.94 × 10^+01^	5	1.34 × 10^+03^ ± 3.94 × 10^+02^	2	1.34 × 10^+03^ ± 3.76 × 10^+02^	3	1.40 × 10^+03^ ± 4.08 × 10^+02^	4
F27(CEC2017)	5.11 × 10^+02^ ± 4.27 × 10^+00^	2	**5.09 × 10^+02^ ± 7.70 × 10^+00^**	**1**	5.11 × 10^+02^ ± 4.73 × 10^+00^	3	5.15 × 10^+02^ ± 5.67 × 10^+00^	5	5.12 × 10^+02^ ± 8.85 × 10^+00^	4
F28(CEC2017)	4.15 × 10^+02^ ± 4.60 × 10^+01^	5	**3.69 × 10^+02^ ± 6.12 × 10^+01^**	**1**	4.13 × 10^+02^ ± 5.55 × 10^+01^	4	3.99 × 10^+02^ ± 6.20 × 10^+01^	3	3.84 × 10^+02^ ± 6.09 × 10^+01^	2
F29(CEC2017)	**5.09 × 10^+02^ ± 4.51 × 10^+01^**	**1**	5.51 × 10^+02^ ± 6.19 × 10^+01^	4	5.13 × 10^+02^ ± 8.15 × 10^+01^	2	5.18 × 10^+02^ ± 4.57 × 10^+01^	3	5.75 × 10^+02^ ± 1.18 × 10^+02^	5
F30(CEC2017)	7.19 × 10^+03^ ± 5.34 × 10^+03^	4	7.04 × 10^+03^ ± 3.01 × 10^+03^	3	6.33 × 10^+03^ ± 3.13 × 10^+03^	2	8.93 × 10^+03^ ± 3.81 × 10^+03^	5	**6.27 × 10^+03^ ± 4.07 × 10^+03^**	**1**
**Average rank**	3.29		2.90		**2.76**		2.98		3.07	
**Final rank**	5		2		**1**		3		4	
**Best/2nd Best/Worst**	7/9/14		10/6/8		**8/12/3**		9/9/10		8/6/7	

**Table 4:** Parameter settings of the four component PSO variants.

Algorithm	Parameters Setting	Year	Reference
CLPSO	w = 0.9~0.2, *c* = 1.49445, *Pc* = 0.05~0.5, *m* = 5	2006	[10]
GOPSO	w = 0.72984, *c*1 = *c*2 = 1.49618, Po = 0.3	2011	[12]
SL-PSO	$N=M+\left\lfloor D/10\right\rfloor$, M = 100, β=0.01 SMLS, α=0.5	2015	[13]
DLPSO	*w =* 0.99~0.2, *c*1 *=* 2.5~0.5, *c*2 *=* 0.5~2.5	2019	[15]

**Table 5:** The experiment results obtained in 13 simple benchmark functions.

Functions	Criteria	SMLS-PSO	SL-PSO		CLPSO		GOPSO		DLPSO	
F1	mean	**0.00 × 10^+00^**	**0.00** × 10**^+00^**	**=**	6.89 × 10^−15^	**+**	4.74 × 10^−32^	**+**	**0.00 × 10^+00^**	**=**
	std	**0.00 × 10^+00^**	**0.00** × 10^+00^		2.38 × 10^−15^		4.88 × 10^−32^		**0.00 × 10^+00^**	
	rank	**1**	**1**		3		2		**1**	
F2	mean	1.12 × 10^−125^	**2.17 × 10^−137^**	**−**	2.53 × 10^−12^	**+**	4.88 × 10^−43^	**+**	2.23 × 10^−119^	**+**
	std	6.25 × 10^−125^	**4.43 × 10^−137^**		1.12 × 10^−12^		1.78 × 10^−42^		7.59 × 10^−119^	
	rank	2	**1**		5		4		3	
F3	mean	1.23 × 10^−100^	**2.04 × 10^−138^**	**−**	3.58 × 10^−13^	**+**	7.92 × 10^−47^	**+**	3.19 × 10^−120^	**−**
	std	6.54 × 10^−100^	**4.01 × 10^−138^**		1.60 × 10^−13^		3.17 × 10^−46^		1.01 × 10^−119^	
	rank	3	**1**		5		4		2	
F4	mean	8.66 × 10^−186^	7.04 × 10^−237^	**−**	4.93 × 10^−26^	**+**	4.60 × 10^−60^	**+**	**9.37 × 10^−240^**	**−**
	std	0.00 × 10^+00^	0.00 × 10^+00^		3.07 × 10^−26^		1.76 × 10^−59^		**0.00 × 10^+00^**	
	rank	3	2		5		4		**1**	
F5	mean	**2.62 × 10^−17^**	2.42 × 10^+00^	**+**	2.01 × 10^+01^	**+**	8.88 × 10^−01^	**+**	6.73 × 10^−06^	**+**
	std	**8.44 × 10^−17^**	2.36 × 10^+00^		5.46 × 10^+00^		9.64 × 10^−01^		1.26 × 10^−05^	
	rank	**1**	4		5		3		2	
F6	mean	1.15 × 10^−65^	**4.52 × 10^−71^**	**−**	2.61 × 10^−08^	**+**	8.25 × 10^−31^	**+**	8.74 × 10^−63^	**+**
	std	3.53 × 10^−65^	**4.79 × 10^−71^**		7.81 × 10^−09^		3.26 × 10^−30^		1.13 × 10^−62^	
	rank	2	**1**		5		4		3	
F7	mean	1.53 × 10^−09^	**1.60 × 10^−11^**	**−**	3.76 × 10^+03^	**+**	1.06 × 10^+01^	**+**	2.66 × 10^−03^	**+**
	std	2.69 × 10^−09^	**1.97 × 10^−11^**		7.08 × 10^+02^		1.25 × 10^+01^		3.17 × 10^−03^	
	rank	2	**1**		5		4		3	
F8	mean	**3.23 × 10^−02^**	6.67 × 10^−01^	**+**	8.32 × 10^−02^	**+**	6.86 × 10^−01^	**+**	1.51 × 10^−01^	**+**
	std	**3.39 × 10^−02^**	1.13 × 10^−16^		3.48 × 10^−02^		1.06 × 10^−01^		2.83 × 10^−01^	
	rank	**1**	4		2		5		3	
F9	mean	4.81 × 10^−01^	1.51 × 10^+01^	**+**	**4.22 × 10^−08^**	**=**	2.24 × 10^+01^	**+**	2.66 × 10^+00^	**+**
	std	6.73 × 10^−01^	4.36 × 10^+00^		**2.01 × 10^−08^**		5.71 × 10^+00^		1.86 × 10^+00^	
	rank	2	4		**1**		5		3	
F10	mean	**2.09 × 10^−01^**	1.14 × 10^+01^	**+**	9.01 × 10^−01^	**+**	6.56 × 10^+00^	**+**	1.31 × 10^+00^	**+**
	std	**1.07 × 10^−01^**	2.22 × 10^−01^		1.65 × 10^−01^		1.53 × 10^+00^		5.50 × 10^−01^	
	rank	**1**	5		2		4		3	
F11	mean	**7.72 × 10^−01^**	2.27 × 10^+01^	**+**	4.12 × 10^+00^	**+**	3.85 × 10^+01^	**+**	1.15 × 10^+01^	**+**
	std	**1.60 × 10^+00^**	2.85 × 10^+01^		1.55 × 10^+00^		2.82 × 10^+01^		2.12 × 10^+01^	
	rank	**1**	4		2		5		3	
F12	mean	5.56 × 10^−04^	4.77 × 10^−04^	**=**	**7.62 × 10^−09^**	**+**	9.19 × 10^−03^	**+**	2.46 × 10^−03^	**=**
	std	3.10 × 10^−03^	1.85 × 10^−03^		**6.79 × 10^−09^**		1.75 × 10^−02^		4.80 × 10^−03^	
	rank	3	2		**1**		5		4	
F13	mean	7.11 × 10^−15^	**6.42 × 10^−15^**	**=**	2.79 × 10^−06^	**+**	7.56 × 10^−15^	**+**	8.60 × 10^−15^	**+**
	std	4.49 × 10^−15^	**1.43 × 10^−15^**		6.85 × 10^−07^		1.77 × 10^−15^		2.87 × 10^−15^	
	rank	2	**1**		5		3		4	
**Average rank**		**1.85**	2.38		3.54		4.00		2.69	
**Final rank**		**1**	2		4		5		3	
**Best/2nd Best/Worst**		5/5/0	6/2/1		2/3/8		0/1/4		2/2/0	
**+/=/−**				5/3/5		12/1/0		13/0/0		9/2/2

**Table 6:** Parameter settings of the eight PSO variants to be compared.

Algorithm	Parameters Setting	Year	Reference
LDWPSO	w = 0.9~0.4, *c*1 = *c*2 = 2	2002	[35]
OLPSO	*w* = 0.9~0.4, *c* = 2.0, *G* = 5	2011	[11]
HCLPSO	w = 0.99~0.29, *c*1 = 2.5~0.5, *c*2 = 0.5~2.5, *K*: 3~1.5, *n*1 = 0.375 * *N*	2015	[20]
GL-PSO	*w* = 0.7298, *c* = 1.49618, *pm* = 0.01, *sg* = 7	2016	[14]
EPSO	Reference the original paper	2017	[8]
TSLPSO	*w*1 = *w*2 = 0.9 ~ 0.4, *c*1 = *c*2 = 1.49445, *c*3 = 0.5~2.5	2019	[15]
HCLDMS-PSO	*w* = 0.99~0.29, *w_2_* = 0.99~0.29, *c*1 = 2.5~0.5, *c*2 = 0.5~2.5, *pm* = 0.1, n1 = 0.375 * N	2020	[43]
SDPSO	Reference the original paper	2022	[25]

**Table 7:** The experiment results obtained in 30D CEC2017 test suite problems.

Functions	Criteria	SMLS-PSO	SDPSO	HCLDMS-PSO	TSLPSO	EPSO	GL-PSO	HCLPSO	OLPSO	LDWPSO
F1	mean	**3.82 × 10^+01^**	2.66 × 10^+03^	5.16 × 10^+02^	2.24 × 10^+03^	2.30 × 10^+03^	3.39 × 10^+03^	1.52 × 10^+03^	2.98 × 10^+03^	5.38 × 10^+03^
	std	**1.19 × 10^+02^**	3.08 × 10^+03^	6.96 × 10^+02^	2.54 × 10^+03^	3.17 × 10^+03^	2.99 × 10^+03^	1.41 × 10^+03^	2.63 × 10^+03^	6.24 × 10^+03^
	rank	**1**	6	2	4	5	8	3	7	9
F3	mean	**4.25 × 10^−07^**	3.32 × 10^+01^	1.09 × 10^+03^	5.54 × 10^+01^	9.61 × 10^+02^	5.65 × 10^+01^	7.54 × 10^−04^	5.18 × 10^+04^	8.01 × 10^+02^
	std	**4.43 × 10^−07^**	1.76 × 10^+02^	1.18 × 10^+03^	1.34 × 10^+02^	4.53 × 10^+02^	2.65 × 10^+02^	1.19 × 10^−03^	1.49 × 10^+04^	4.67 × 10^+02^
	rank	**1**	3	8	4	7	5	2	9	6
F4	mean	**1.03 × 10^+00^**	7.14 × 10^+01^	7.42 × 10^+01^	3.63 × 10^+01^	5.90 × 10^+01^	3.38 × 10^+01^	5.36 × 10^+01^	9.20 × 10^+01^	9.43 × 10^+01^
	std	**1.77 × 10^+00^**	2.59 × 10^+01^	2.16 × 10^+01^	3.57 × 10^+01^	3.08 × 10^+01^	3.28 × 10^+01^	3.48 × 10^+01^	2.33 × 10^+01^	1.79 × 10^+01^
	rank	**1**	6	7	3	5	2	4	8	9
F5	mean	4.33 × 10^+01^	3.26 × 10^+01^	**2.83 × 10^+01^**	5.21 × 10^+01^	4.76 × 10^+01^	5.38 × 10^+01^	5.29 × 10^+01^	3.93 × 10^+01^	7.05 × 10^+01^
	std	1.20 × 10^+01^	8.52 × 10^+00^	**6.74 × 10^+00^**	1.50 × 10^+01^	9.93 × 10^+00^	1.26 × 10^+01^	1.52 × 10^+01^	1.19 × 10^+01^	1.64 × 10^+01^
	rank	4	2	**1**	6	5	8	7	3	9
F6	mean	1.01 × 10^−02^	1.86 × 10^−01^	3.21 × 10^−03^	**3.19 × 10^−13^**	1.09 × 10^−02^	1.06 × 10^−04^	4.69 × 10^−13^	1.29 × 10^−10^	1.25 × 10^−01^
	std	2.07 × 10^−02^	2.17 × 10^−01^	2.75 × 10^−03^	**6.17 × 10^−14^**	7.30 × 10^−03^	1.70 × 10^−04^	2.19 × 10^−13^	1.51 × 10^−10^	1.97 × 10^−01^
	rank	6	9	5	**1**	7	4	2	3	8
F7	mean	8.27 × 10^+01^	8.04 × 10^+01^	**6.47 × 10^+01^**	8.54 × 10^+01^	1.07 × 10^+02^	8.45 × 10^+01^	9.04 × 10^+01^	8.23 × 10^+01^	1.20 × 10^+02^
	std	1.27 × 10^+01^	2.13 × 10^+01^	**6.78 × 10^+00^**	1.41 × 10^+01^	3.01 × 10^+01^	1.30 × 10^+01^	1.54 × 10^+01^	2.04 × 10^+01^	1.80 × 10^+01^
	rank	4	2	**1**	6	8	5	7	3	9
F8	mean	4.66 × 10^+01^	3.17 × 10^+01^	**2.62 × 10^+01^**	5.52 × 10^+01^	5.20 × 10^+01^	5.59 × 10^+01^	5.36 × 10^+01^	3.73 × 10^+01^	6.76 × 10^+01^
	std	1.21 × 10^+01^	1.02 × 10^+01^	**5.56 × 10^+00^**	1.29 × 10^+01^	9.53 × 10^+00^	1.30 × 10^+01^	1.20 × 10^+01^	1.28 × 10^+01^	1.80 × 10^+01^
	rank	4	2	**1**	7	5	8	6	3	9
F9	mean	1.09 × 10^+01^	1.14 × 10^+01^	**5.51 × 10^−02^**	1.20 × 10^+01^	1.46 × 10^+01^	2.95 × 10^+01^	1.84 × 10^+01^	3.16 × 10^−01^	2.15 × 10^+02^
	std	8.17 × 10^+00^	2.30 × 10^+01^	**1.64 × 10^−01^**	1.79 × 10^+01^	9.71 × 10^+00^	6.06 × 10^+01^	2.48 × 10^+01^	4.67 × 10^−01^	3.46 × 10^+02^
	rank	3	4	**1**	5	6	8	7	2	9
F10	mean	2.75 × 10^+03^	2.74 × 10^+03^	2.39 × 10^+03^	2.67 × 10^+03^	**2.06 × 10^+03^**	2.71 × 10^+03^	2.24 × 10^+03^	2.15 × 10^+03^	3.16 × 10^+03^
	std	7.85 × 10^+02^	4.81 × 10^+02^	4.69 × 10^+02^	4.18 × 10^+02^	**4.19 × 10^+02^**	6.10 × 10^+02^	3.27 × 10^+02^	5.48 × 10^+02^	6.20 × 10^+02^
	rank	8	7	4	5	**1**	6	3	2	9
F11	mean	**3.05 × 10^+01^**	1.06 × 10^+02^	3.37 × 10^+01^	4.95 × 10^+01^	6.42 × 10^+01^	6.65 × 10^+01^	4.94 × 10^+01^	4.38 × 10^+01^	8.86 × 10^+01^
	std	**1.58 × 10^+01^**	4.66 × 10^+01^	1.94 × 10^+01^	2.64 × 10^+01^	2.17 × 10^+01^	3.90 × 10^+01^	3.02 × 10^+01^	3.94 × 10^+01^	3.40 × 10^+01^
	rank	**1**	9	2	5	6	7	4	3	8
F12	mean	**1.26 × 10^+03^**	7.69 × 10^+04^	5.40 × 10^+04^	2.55 × 10^+04^	4.25 × 10^+05^	1.91 × 10^+04^	2.86 × 10^+04^	1.84 × 10^+05^	4.60 × 10^+04^
	std	**4.29 × 10^+02^**	4.85 × 10^+04^	3.78 × 10^+04^	2.06 × 10^+04^	3.53 × 10^+05^	1.25 × 10^+04^	1.55 × 10^+04^	2.67 × 10^+05^	2.28 × 10^+04^
	rank	**1**	7	6	3	9	2	4	8	5
F13	mean	**2.25 × 10^+02^**	8.31 × 10^+03^	1.01 × 10^+04^	1.08 × 10^+04^	1.34 × 10^+03^	1.39 × 10^+04^	3.52 × 10^+03^	1.57 × 10^+04^	1.55 × 10^+04^
	std	**4.61 × 10^+02^**	6.62 × 10^+03^	8.24 × 10^+03^	1.17 × 10^+04^	1.16 × 10^+03^	1.46 × 10^+04^	3.37 × 10^+03^	1.94 × 10^+04^	1.75 × 10^+04^
	rank	**1**	4	5	6	2	7	3	9	8
F14	mean	**3.42 × 10^+02^**	6.61 × 10^+03^	1.51 × 10^+03^	1.68 × 10^+04^	9.31 × 10^+03^	4.27 × 10^+03^	7.07 × 10^+03^	2.64 × 10^+04^	1.15 × 10^+04^
	std	**9.56 × 10^+02^**	7.41 × 10^+03^	1.50 × 10^+03^	1.43 × 10^+04^	7.66 × 10^+03^	4.89 × 10^+03^	5.39 × 10^+03^	2.31 × 10^+04^	1.05 × 10^+04^
	rank	**1**	4	2	8	6	3	5	9	7
F15	mean	**2.15 × 10^+02^**	2.71 × 10^+03^	1.97 × 10^+03^	7.01 × 10^+03^	2.22 × 10^+02^	4.50 × 10^+03^	3.67 × 10^+02^	9.65 × 10^+03^	7.40 × 10^+03^
	std	**6.24 × 10^+02^**	3.42 × 10^+03^	3.00 × 10^+03^	9.83 × 10^+03^	1.77 × 10^+02^	4.53 × 10^+03^	3.10 × 10^+02^	1.05 × 10^+04^	7.00 × 10^+03^
	rank	**1**	5	4	7	2	6	3	9	8
F16	mean	4.20 × 10^+02^	4.89 × 10^+02^	**3.28 × 10^+02^**	5.72 × 10^+02^	5.09 × 10^+02^	8.04 × 10^+02^	5.67 × 10^+02^	7.40 × 10^+02^	8.21 × 10^+02^
	std	2.50 × 10^+02^	2.48 × 10^+02^	**1.81 × 10^+02^**	1.43 × 10^+02^	1.67 × 10^+02^	3.11 × 10^+02^	1.53 × 10^+02^	2.59 × 10^+02^	2.42 × 10^+02^
	rank	2	3	**1**	6	4	8	5	7	9
F17	mean	1.17 × 10^+02^	1.20 × 10^+02^	**8.91 × 10^+01^**	1.32 × 10^+02^	1.39 × 10^+02^	3.22 × 10^+02^	1.33 × 10^+02^	2.46 × 10^+02^	2.25 × 10^+02^
	std	7.56 × 10^+01^	9.16 × 10^+01^	**4.97 × 10^+01^**	7.10 × 10^+01^	7.67 × 10^+01^	1.73 × 10^+02^	8.15 × 10^+01^	1.33 × 10^+02^	1.47 × 10^+02^
	rank	2	3	**1**	4	6	9	5	8	7
F18	mean	**7.87 × 10^+01^**	1.60 × 10^+05^	6.57 × 10^+04^	1.15 × 10^+05^	1.19 × 10^+05^	5.85 × 10^+04^	7.78 × 10^+04^	3.34 × 10^+05^	1.74 × 10^+05^
	std	**4.42 × 10^+01^**	8.09 × 10^+04^	3.37 × 10^+04^	8.05 × 10^+04^	5.56 × 10^+04^	3.32 × 10^+04^	5.76 × 10^+04^	3.15 × 10^+05^	2.11 × 10^+05^
	rank	**1**	7	3	5	6	2	4	9	8
F19	mean	**3.49 × 10^+01^**	5.40 × 10^+03^	2.31 × 10^+03^	3.34 × 10^+03^	8.58 × 10^+01^	4.78 × 10^+03^	1.48 × 10^+02^	1.10 × 10^+04^	8.84 × 10^+03^
	std	**2.27 × 10^+01^**	5.87 × 10^+03^	2.66 × 10^+03^	3.67 × 10^+03^	9.13 × 10^+01^	5.02 × 10^+03^	1.03 × 10^+02^	1.34 × 10^+04^	1.10 × 10^+04^
	rank	**1**	7	4	5	2	6	3	9	8
F20	mean	**1.31 × 10^+02^**	2.03 × 10^+02^	1.70 × 10^+02^	1.83 × 10^+02^	1.97 × 10^+02^	3.61 × 10^+02^	1.64 × 10^+02^	2.74 × 10^+02^	2.85 × 10^+02^
	std	**7.78 × 10^+01^**	6.48 × 10^+01^	5.43 × 10^+01^	7.35 × 10^+01^	9.98 × 10^+01^	1.47 × 10^+02^	8.93 × 10^+01^	1.39 × 10^+02^	1.30 × 10^+02^
	rank	**1**	6	3	4	5	9	2	7	8
F21	mean	2.42 × 10^+02^	2.36 × 10^+02^	**2.26 × 10^+02^**	2.55 × 10^+02^	2.32 × 10^+02^	2.58 × 10^+02^	2.38 × 10^+02^	2.46 × 10^+02^	2.77 × 10^+02^
	std	1.02 × 10^+01^	1.26 × 10^+01^	**5.74 × 10^+00^**	2.86 × 10^+01^	5.69 × 10^+01^	1.28 × 10^+01^	4.60 × 10^+01^	1.19 × 10^+01^	1.74 × 10^+01^
	rank	5	3	**1**	7	2	8	4	6	9
F22	mean	1.00 × 10^+02^	1.01 × 10^+02^	**1.00 × 10^+02^**	1.73 × 10^+02^	1.00 × 10^+02^	5.16 × 10^+02^	1.01 × 10^+02^	9.64 × 10^+02^	1.54 × 10^+03^
	std	1.83 × 10^−06^	1.44 × 10^+00^	**9.00 × 10^−13^**	3.60 × 10^+02^	9.47 × 10^−01^	1.11 × 10^+03^	1.13 × 10^+00^	1.20 × 10^+03^	1.76 × 10^+03^
	rank	2	5	**1**	6	3	7	4	8	9
F23	mean	3.90 × 10^+02^	3.95 × 10^+02^	**3.73 × 10^+02^**	4.13 × 10^+02^	3.87 × 10^+02^	4.16 × 10^+02^	4.09 × 10^+02^	3.97 × 10^+02^	4.36 × 10^+02^
	std	1.20 × 10^+01^	1.78 × 10^+01^	**1.03 × 10^+01^**	1.70 × 10^+01^	5.48 × 10^+01^	1.79 × 10^+01^	1.19 × 10^+01^	1.63 × 10^+01^	2.12 × 10^+01^
	rank	3	4	**1**	7	2	8	6	5	9
F24	mean	4.60 × 10^+02^	4.62 × 10^+02^	**4.47 × 10^+02^**	4.90 × 10^+02^	4.53 × 10^+02^	4.84 × 10^+02^	4.79 × 10^+02^	4.79 × 10^+02^	5.10 × 10^+02^
	std	1.11 × 10^+01^	1.73 × 10^+01^	**7.72 × 10^+00^**	5.40 × 10^+01^	8.44 × 10^+01^	2.37 × 10^+01^	4.40 × 10^+01^	1.34 × 10^+01^	2.38 × 10^+01^
	rank	3	4	**1**	8	2	7	6	5	9
F25	mean	**3.80 × 10^+02^**	3.89 × 10^+02^	3.87 × 10^+02^	3.87 × 10^+02^	3.87 × 10^+02^	3.87 × 10^+02^	3.87 × 10^+02^	3.87 × 10^+02^	3.89 × 10^+02^
	std	**6.27 × 10^−01^**	7.05 × 10^+00^	1.01 × 10^+00^	1.23 × 10^+00^	1.15 × 10^+00^	1.61 × 10^+00^	1.39 × 10^+00^	1.04 × 10^+00^	7.03 × 10^+00^
	rank	**1**	8	2	4	6	3	7	5	9
F26	mean	1.34 × 10^+03^	1.29 × 10^+03^	1.19 × 10^+03^	1.35 × 10^+03^	**3.61 × 10^+02^**	1.65 × 10^+03^	6.10 × 10^+02^	1.50 × 10^+03^	1.79 × 10^+03^
	std	3.67 × 10^+02^	4.28 × 10^+02^	8.65 × 10^+01^	6.43 × 10^+02^	**3.41 × 10^+02^**	5.28 × 10^+02^	4.75 × 10^+02^	2.71 × 10^+02^	5.76 × 10^+02^
	rank	5	4	3	6	**1**	8	2	7	9
F27	mean	**5.05 × 10^+02^**	5.41 × 10^+02^	5.16 × 10^+02^	5.21 × 10^+02^	5.11 × 10^+02^	5.30 × 10^+02^	5.18 × 10^+02^	5.18 × 10^+02^	5.33 × 10^+02^
	std	**1.53 × 10^+01^**	1.39 × 10^+01^	7.35 × 10^+00^	8.59 × 10^+00^	6.68 × 10^+00^	1.18 × 10^+01^	5.99 × 10^+00^	9.32 × 10^+00^	1.20 × 10^+01^
	rank	**1**	9	3	6	2	7	4	5	8
F28	mean	3.55 × 10^+02^	3.40 × 10^+02^	3.90 × 10^+02^	3.60 × 10^+02^	4.00 × 10^+02^	**3.15 × 10^+02^**	3.59 × 10^+02^	4.14 × 10^+02^	4.11 × 10^+02^
	std	6.75 × 10^+01^	5.95 × 10^+01^	3.75 × 10^+01^	6.12 × 10^+01^	2.46 × 10^+01^	**3.67 × 10^+01^**	6.11 × 10^+01^	1.90 × 10^+01^	2.44 × 10^+01^
	rank	3	2	6	5	7	**1**	4	9	8
F29	mean	5.48 × 10^+02^	6.42 × 10^+02^	**4.87 × 10^+02^**	6.64 × 10^+02^	5.04 × 10^+02^	6.53 × 10^+02^	5.90 × 10^+02^	5.99 × 10^+02^	6.90 × 10^+02^
	std	7.71 × 10^+01^	1.27 × 10^+02^	**2.19 × 10^+01^**	1.44 × 10^+02^	5.69 × 10^+01^	1.70 × 10^+02^	9.40 × 10^+01^	1.24 × 10^+02^	1.70 × 10^+02^
	rank	3	6	**1**	8	2	7	4	5	9
F30	mean	**3.38 × 10^+03^**	5.07 × 10^+03^	3.70 × 10^+03^	7.91 × 10^+03^	4.18 × 10^+03^	4.59 × 10^+03^	4.78 × 10^+03^	6.11 × 10^+03^	5.24 × 10^+03^
	std	**1.08 × 10^+03^**	1.62 × 10^+03^	1.04 × 10^+03^	4.44 × 10^+03^	1.39 × 10^+03^	2.25 × 10^+03^	2.18 × 10^+03^	3.22 × 10^+03^	2.54 × 10^+03^
	rank	**1**	6	2	9	3	4	5	8	7
**Average rank**		**2.45**	5.07	2.83	5.52	4.38	5.97	4.31	6.24	8.24
**Final rank**		**1**	5	2	6	4	7	3	8	9
**Best/2nd Best/Worst**		14/3/0	0/4/3	11/5/0	1/0/1	2/8/1	1/3/2	0/4/0	0/2/7	0/0/15

**Table 8:** Wilcoxon rank sum test results for 30D CEC2017 test suite problems.

Functions	Pairwise Comparison SMLS-PSO Versus							
	SDPSO	HCLDMS-PSO	TSLPSO	EPSO	GL-PSO	HCLPSO	OLPSO	LDWPSO
F1	+	+	+	+	+	+	+	+
F3	+	+	+	+	+	+	+	+
F4	+	+	+	+	+	+	+	+
F5	−	−	+	=	+	+	=	+
F6	+	=	−	+	−	−	−	+
F7	=	−	=	+	−	+	=	+
F8	−	−	+	=	+	+	−	+
F9	=	−	=	=	=	=	−	+
F10	=	=	=	−	=	−	−	+
F11	+	=	+	+	+	+	=	+
F12	+	+	+	+	+	+	+	+
F13	+	+	+	+	+	+	+	+
F14	+	+	+	+	+	+	+	+
F15	+	+	+	+	+	+	+	+
F16	=	=	+	=	+	+	+	+
F17	=	=	=	=	+	=	+	+
F18	+	+	+	+	+	+	+	+
F19	+	+	+	+	+	+	+	+
F20	+	+	+	+	+	=	+	+
F21	−	−	+	+	+	+	=	+
F22	+	=	=	+	−	=	+	=
F23	=	−	+	=	+	+	=	+
F24	=	−	+	+	+	+	+	+
F25	+	+	+	+	+	+	+	+
F26	=	−	=	−	+	−	+	+
F27	+	+	+	=	+	+	+	+
F28	=	+	=	+	−	=	+	+
F29	+	−	+	−	+	−	=	+
F30	+	+	+	+	+	+	+	+
+/=/−	17/9/3	14/6/9	21/7/1	19/7/3	23/3/3	20/6/3	19/6/4	28/1/0

**Table 9:** Ranks obtained through the Friedman test for 30D CEC2017 test suite problems.

Algorithm	SMLS-PSO	SDPSO	HCLDMS-PSO	TSLPSO	EPSO	GL-PSO	HCLPSO	OLPSO	LDWPSO
Average rank	**3.15**	5.36	3.70	5.32	4.98	5.38	4.58	5.57	6.97
Final rank	**1**	6	2	5	4	7	3	8	9
*p*-value	1.0960 × 10^−17^								

**Table 10:** The experiment results obtained in 50D CEC2017 test suite problems.

Functions	Criteria	SMLS-PSO	SDPSO	HCLDMS-PSO	TSLPSO	EPSO	GL-PSO	HCLPSO	OLPSO	LDWPSO
F1	mean	**1.09s × 10^+01^**	2.61 × 10^+03^	1.68 × 10^+03^	6.42 × 10^+03^	6.77 × 10^+04^	2.54 × 10^+03^	5.45 × 10^+03^	1.06 × 10^+04^	7.70 × 10^+03^
	std	**4.06 × 10^+01^**	3.61 × 10^+03^	2.83 × 10^+03^	7.05 × 10^+03^	5.65 × 10^+04^	3.05 × 10^+03^	5.67 × 10^+03^	6.12 × 10^+03^	8.26 × 10^+03^
	rank	**1**	4	2	6	9	3	5	8	7
F3	mean	**3.00 × 10^−06^**	3.06 × 10^+02^	2.00 × 10^+04^	6.63 × 10^+03^	9.63 × 10^+03^	1.01 × 10^+04^	2.25 × 10^+01^	1.64 × 10^+05^	1.47 × 10^+04^
	std	**2.52 × 10^−06^**	6.31 × 10^+02^	9.03 × 10^+03^	3.89 × 10^+03^	2.87 × 10^+03^	7.58 × 10^+03^	2.08 × 10^+01^	2.66 × 10^+04^	3.49 × 10^+03^
	rank	**1**	3	8	4	5	6	2	9	7
F4	mean	**8.60 × 10^−01^**	1.05 × 10^+02^	8.64 × 10^+01^	8.89 × 10^+01^	5.83 × 10^+01^	9.31 × 10^+01^	9.99 × 10^+01^	1.30 × 10^+02^	1.34 × 10^+02^
	std	**1.66 × 10^+00^**	5.30 × 10^+01^	3.45 × 10^+01^	5.07 × 10^+01^	3.74 × 10^+01^	4.75 × 10^+01^	5.22 × 10^+01^	5.56 × 10^+01^	4.98 × 10^+01^
	rank	**1**	7	3	4	2	5	6	8	9
F5	mean	9.28 × 10^+01^	8.81 × 10^+01^	**5.84 × 10^+01^**	1.33 × 10^+02^	1.15 × 10^+02^	1.24 × 10^+02^	1.39 × 10^+02^	8.32 × 10^+01^	1.85 × 10^+02^
	std	1.85 × 10^+01^	2.44 × 10^+01^	**1.12 × 10^+01^**	2.96 × 10^+01^	1.77 × 10^+01^	2.53 × 10^+01^	2.38 × 10^+01^	2.18 × 10^+01^	4.13 × 10^+01^
	rank	4	3	**1**	7	5	6	8	2	9
F6	mean	2.57 × 10^−02^	2.95 × 10^+00^	2.29 × 10^−02^	**4.59 × 10^−13^**	2.05 × 10^−02^	5.55 × 10^−03^	1.16 × 10^−05^	4.63 × 10^−05^	3.16 × 10^+00^
	std	2.63 × 10^−02^	3.08 × 10^+00^	1.35 × 10^−02^	**6.81 × 10^−14^**	6.31 × 10^−03^	4.20 × 10^−03^	8.27 × 10^−05^	2.43 × 10^−05^	2.38 × 10^+00^
	rank	7	8	6	**1**	5	4	2	3	9
F7	mean	1.74 × 10^+02^	1.59 × 10^+02^	**1.22 × 10^+02^**	1.68 × 10^+02^	1.96 × 10^+02^	1.76 × 10^+02^	1.99 × 10^+02^	2.92 × 10^+02^	2.75 × 10^+02^
	std	2.98 × 10^+01^	2.84 × 10^+01^	**1.97 × 10^+01^**	2.79 × 10^+01^	4.87 × 10^+01^	3.01 × 10^+01^	2.33 × 10^+01^	6.73 × 10^+01^	4.83 × 10^+01^
	rank	4	2	**1**	3	6	5	7	9	8
F8	mean	8.52 × 10^+01^	8.42 × 10^+01^	**5.50 × 10^+01^**	1.32 × 10^+02^	1.19 × 10^+02^	1.18 × 10^+02^	1.40 × 10^+02^	8.48 × 10^+01^	1.79 × 10^+02^
	std	1.73 × 10^+01^	1.85 × 10^+01^	**9.26 × 10^+00^**	3.20 × 10^+01^	1.87 × 10^+01^	2.29 × 10^+01^	2.42 × 10^+01^	2.24 × 10^+01^	3.19 × 10^+01^
	rank	4	2	**1**	7	6	5	8	3	9
F9	mean	7.51 × 10^+01^	2.13 × 10^+02^	**1.99 × 10^+00^**	7.74 × 10**^+02^**	2.16 × 10**^+02^**	7.71 × 10**^+02^**	8.87 × 10**^+02^**	5.06 × 10**^+00^**	2.05 × 10**^+03^**
	std	5.38 × 10^+01^	2.38 × 10^+02^	**2.00 × 10^+00^**	8.64 × 10**^+02^**	1.76 × 10**^+02^**	7.87 × 10**^+02^**	1.24 × 10**^+03^**	8.35 × 10**^+00^**	1.18 × 10**^+03^**
	rank	3	4	**1**	7	5	6	8	2	9
F10	mean	5.32 × 10**^+03^**	5.18 × 10**^+03^**	4.57 × 10**^+03^**	5.20 × 10**^+03^**	**3.69 × 10^+03^**	4.98 × 10**^+03^**	4.17 × 10**^+03^**	4.34 × 10**^+03^**	5.77 × 10**^+03^**
	std	9.67 × 10**^+02^**	7.56 × 10**^+02^**	4.45 × 10**^+02^**	7.39 × 10**^+02^**	**5.01 × 10^+02^**	7.31 × 10**^+02^**	5.64 × 10**^+02^**	7.97 × 10**^+02^**	9.56 × 10**^+02^**
	rank	8	6	4	7	**1**	5	2	3	9
F11	mean	9.11 × 10^+01^	1.79 × 10^+02^	9.81 × 10^+01^	1.17 × 10^+02^	1.44 × 10^+02^	1.06 × 10^+02^	1.12 × 10^+02^	**8.85 × 10^+01^**	1.84 × 10^+02^
	std	3.25 × 10^+01^	5.24 × 10^+01^	2.77 × 10^+01^	3.53 × 10^+01^	3.68 × 10^+01^	3.55 × 10^+01^	3.80 × 10^+01^	**3.15 × 10^+01^**	5.96 × 10^+01^
	rank	2	8	3	6	7	4	5	**1**	9
F12	mean	**6.18 × 10^+03^**	7.46 × 10^+05^	1.14 × 10^+06^	5.55 × 10^+05^	2.50 × 10^+06^	1.49 × 10^+05^	4.50 × 10^+05^	1.92 × 10^+06^	1.16 × 10^+06^
	std	**1.47 × 10^+04^**	4.30 × 10^+05^	7.11 × 10^+05^	3.90 × 10^+05^	1.81 × 10^+06^	9.63 × 10^+04^	2.60 × 10^+05^	1.13 × 10^+06^	7.21 × 10^+05^
	rank	**1**	5	6	4	9	2	3	8	7
F13	mean	**1.42 × 10^+02^**	5.74 × 10^+03^	1.15 × 10^+03^	4.27 × 10^+03^	1.72 × 10^+03^	3.49 × 10^+03^	4.93 × 10^+03^	4.62 × 10^+03^	5.93 × 10^+03^
	std	**9.33 × 10^+01^**	6.65 × 10^+03^	1.35 × 10^+03^	5.32 × 10^+03^	8.93 × 10^+02^	3.42 × 10^+03^	5.05 × 10^+03^	5.53 × 10^+03^	6.67 × 10^+03^
	rank	**1**	8	2	5	3	4	7	6	9
F14	mean	**2.88 × 10^+02^**	2.22 × 10^+04^	3.15 × 10^+04^	6.38 × 10^+04^	4.47 × 10^+04^	2.97 × 10^+04^	3.91 × 10^+04^	1.39 × 10^+05^	4.37 × 10^+04^
	std	**1.59 × 10^+02^**	1.52 × 10^+04^	2.26 × 10^+04^	6.62 × 10^+04^	2.60 × 10^+04^	2.89 × 10^+04^	3.02 × 10^+04^	1.11 × 10^+05^	4.58 × 10^+04^
	rank	**1**	2	4	8	7	3	5	9	6
F15	mean	2.38 × 10^+03^	3.15 × 10^+03^	1.83 × 10^+03^	5.46 × 10^+03^	**3.90 × 10^+02^**	5.24 × 10^+03^	3.12 × 10^+03^	7.44 × 10^+03^	7.24 × 10^+03^
	std	3.10 × 10^+03^	3.66 × 10^+03^	2.65 × 10^+03^	6.08 × 10^+03^	**2.50 × 10^+02^**	5.18 × 10^+03^	3.55 × 10^+03^	7.75 × 10^+03^	5.73 × 10^+03^
	rank	3	5	2	7	**1**	6	4	9	8
F16	mean	1.06 × 10^+03^	9.79 × 10^+02^	**7.21 × 10^+02^**	1.24 × 10^+03^	1.09 × 10^+03^	1.37 × 10^+03^	1.17 × 10^+03^	1.37 × 10^+03^	1.66 × 10^+03^
	std	3.35 × 10^+02^	3.96 × 10^+02^	**2.10 × 10^+02^**	3.26 × 10^+02^	2.41 × 10^+02^	3.96 × 10^+02^	2.21 × 10^+02^	3.69 × 10^+02^	4.37 × 10^+02^
	rank	3	2	**1**	6	4	7	5	8	9
F17	mean	7.39 × 10^+02^	8.80 × 10^+02^	**5.60 × 10^+02^**	7.99 × 10^+02^	8.00 × 10^+02^	1.03 × 10^+03^	7.62 × 10^+02^	9.64 × 10^+02^	1.21 × 10^+03^
	std	2.21 × 10^+02^	2.97 × 10^+02^	**1.63 × 10^+02^**	2.10 × 10^+02^	1.43 × 10^+02^	2.71 × 10^+02^	1.46 × 10^+02^	2.33 × 10^+02^	2.78 × 10^+02^
	rank	2	6	**1**	4	5	8	3	7	9
F18	mean	**1.08 × 10^+02^**	2.12 × 10^+05^	2.24 × 10^+05^	2.18 × 10^+05^	3.84 × 10^+05^	9.55 × 10^+04^	1.32 × 10^+05^	1.97 × 10^+06^	3.06 × 10^+05^
	std	**4.63 × 10^+01^**	2.44 × 10^+05^	1.42 × 10^+05^	1.66 × 10^+05^	3.21 × 10^+05^	5.88 × 10^+04^	1.04 × 10^+05^	1.54 × 10^+06^	1.92 × 10^+05^
	rank	**1**	4	6	5	8	2	3	9	7
F19	mean	**9.24 × 10^+01^**	1.45 × 10^+04^	6.30 × 10^+03^	1.20 × 10^+04^	2.95 × 10^+02^	1.38 × 10^+04^	1.83 × 10^+02^	1.77 × 10^+04^	1.74 × 10^+04^
	std	**9.41 × 10^+01^**	7.46 × 10^+03^	5.42 × 10^+03^	9.01 × 10^+03^	5.34 × 10^+02^	7.09 × 10^+03^	2.03 × 10^+02^	1.31 × 10^+04^	1.24 × 10^+04^
	rank	**1**	7	4	5	3	6	2	9	8
F20	mean	5.83 × 10^+02^	5.40 × 10^+02^	**2.80 × 10^+02^**	6.09 × 10^+02^	5.94 × 10^+02^	8.41 × 10^+02^	6.32 × 10^+02^	7.59 × 10^+02^	9.22 × 10^+02^
	std	2.63 × 10^+02^	1.77 × 10^+02^	**1.37 × 10^+02^**	1.88 × 10^+02^	1.65 × 10^+02^	2.33 × 10^+02^	1.56 × 10^+02^	3.03 × 10^+02^	3.33 × 10^+02^
	rank	3	2	**1**	5	4	8	6	7	9
F21	mean	2.82 × 10^+02^	2.85 × 10^+02^	**2.62 × 10^+02^**	3.48 × 10^+02^	3.19 × 10^+02^	3.28 × 10^+02^	3.46 × 10^+02^	2.93 × 10^+02^	3.91 × 10^+02^
	std	1.63 × 10^+01^	2.60 × 10^+01^	**1.07 × 10^+01^**	3.38 × 10^+01^	3.36 × 10^+01^	2.81 × 10^+01^	2.31 × 10^+01^	1.84 × 10^+01^	3.63 × 10^+01^
	rank	2	3	**1**	8	5	6	7	4	9
F22	mean	4.90 × 10^+03^	3.32 × 10^+03^	2.79 × 10^+03^	4.64 × 10^+03^	**1.87 × 10^+03^**	5.72 × 10^+03^	4.27 × 10^+03^	4.69 × 10^+03^	6.29 × 10^+03^
	std	2.30 × 10^+03^	3.03 × 10^+03^	2.59 × 10^+03^	2.57 × 10^+03^	**2.16 × 10^+03^**	8.37 × 10^+02^	1.63 × 10^+03^	1.45 × 10^+03^	1.47 × 10^+03^
	rank	7	3	2	5	**1**	8	4	6	9
F23	mean	5.15 × 10^+02^	5.70 × 10^+02^	**4.95 × 10^+02^**	6.03 × 10^+02^	5.48 × 10^+02^	5.90 × 10^+02^	5.87 × 10^+02^	5.36 × 10^+02^	6.76 × 10^+02^
	std	2.00 × 10^+01^	4.28 × 10^+01^	**1.63 × 10^+01^**	4.64 × 10^+01^	3.32 × 10^+01^	3.08 × 10^+01^	2.57 × 10^+01^	2.55 × 10^+01^	5.80 × 10^+01^
	rank	2	5	**1**	8	4	7	6	3	9
F24	mean	5.79 × 10^+02^	6.14 × 10^+02^	**5.65 × 10^+02^**	7.29 × 10^+02^	6.12 × 10^+02^	6.68 × 10^+02^	6.85 × 10^+02^	6.18 × 10^+02^	7.39 × 10^+02^
	std	2.01 × 10^+01^	4.00 × 10^+01^	**1.90 × 10^+01^**	4.99 × 10^+01^	3.61 × 10^+01^	5.50 × 10^+01^	5.97 × 10^+01^	2.55 × 10^+01^	6.13 × 10^+01^
	rank	2	4	**1**	8	3	6	7	5	9
F25	mean	**4.66 × 10^+02^**	5.41 × 10^+02^	5.32 × 10^+02^	5.31 × 10^+02^	5.12 × 10^+02^	5.43 × 10^+02^	5.44 × 10^+02^	5.46 × 10^+02^	5.50 × 10^+02^
	std	**2.86 × 10^+01^**	3.59 × 10^+01^	3.18 × 10^+01^	3.89 × 10^+01^	2.89 × 10^+01^	4.55 × 10^+01^	4.24 × 10^+01^	2.53 × 10^+01^	2.85 × 10^+01^
	rank	**1**	5	4	3	2	6	7	8	9
F26	mean	2.10 × 10^+03^	2.42 × 10^+03^	1.68 × 10^+03^	2.82 × 10^+03^	**9.71 × 10^+02^**	2.95 × 10^+03^	1.77 × 10^+03^	2.19 × 10^+03^	3.53 × 10^+03^
	std	2.10 × 10^+02^	3.67 × 10^+02^	1.30 × 10^+02^	3.83 × 10^+02^	**1.00 × 10^+03^**	4.85 × 10^+02^	1.10 × 10^+03^	2.83 × 10^+02^	5.40 × 10^+02^
	rank	4	6	2	7	**1**	8	3	5	9
F27	mean	**5.79 × 10^+02^**	8.46 × 10^+02^	6.33 × 10^+02^	7.72 × 10^+02^	6.15 × 10^+02^	7.30 × 10^+02^	6.73 × 10^+02^	6.43 × 10^+02^	8.15 × 10^+02^
	std	**4.57 × 10^+01^**	8.77 × 10^+01^	4.23 × 10^+01^	6.57 × 10^+01^	2.57 × 10^+01^	7.03 × 10^+01^	4.48 × 10^+01^	4.97 × 10^+01^	9.13 × 10^+01^
	rank	**1**	9	3	7	2	6	5	4	8
F28	mean	**4.44 × 10^+02^**	4.94 × 10^+02^	4.81 × 10^+02^	5.06 × 10^+02^	4.84 × 10^+02^	4.93 × 10^+02^	4.96 × 10^+02^	5.03 × 10^+02^	5.02 × 10^+02^
	std	**8.13 × 10^+00^**	2.46 × 10^+01^	2.29 × 10^+01^	1.49 × 10^+01^	1.89 × 10^+01^	1.61 × 10^+01^	2.60 × 10^+01^	1.92 × 10^+01^	2.48 × 10^+01^
	rank	**1**	5	2	9	3	4	6	8	7
F29	mean	**7.18 × 10^+02^**	1.04 × 10^+03^	7.42 × 10^+02^	1.16 × 10^+03^	7.55 × 10^+02^	1.04 × 10^+03^	8.20 × 10^+02^	7.60 × 10^+02^	1.19 × 10^+03^
	std	**1.68 × 10^+02^**	3.01 × 10^+02^	1.75 × 10^+02^	3.10 × 10^+02^	1.43 × 10^+02^	2.61 × 10^+02^	1.73 × 10^+02^	2.34 × 10^+02^	2.67 × 10^+02^
	rank	**1**	6	2	8	3	7	5	4	9
F30	mean	**2.96 × 10^+03^**	1.00 × 10^+06^	8.79 × 10^+05^	4.49 × 10^+06^	7.00 × 10^+05^	8.95 × 10^+05^	7.92 × 10^+05^	1.01 × 10^+06^	1.16 × 10^+06^
	std	**7.39 × 10^+02^**	1.63 × 10^+05^	1.07 × 10^+05^	1.36 × 10^+07^	4.43 × 10^+04^	1.60 × 10^+05^	7.15 × 10^+04^	3.35 × 10^+05^	3.71 × 10^+05^
	rank	**1**	6	4	9	2	5	3	7	8
**Average rank**		**2.52**	4.83	2.72	5.97	4.17	5.45	4.97	6.00	8.38
**Final rank**		**1**	4	2	7	3	6	5	8	9
**Best/2nd Best/Worst**		13/5/0	0/5/1	10/7/0	1/0/2	4/4/2	0/2/0	0/4/0	1/2/6	0/0/18

**Table 11:** Wilcoxon rank sum test results for 50D CEC2017 test suite problems.

Functions	Pairwise Comparison SMLS-PSO Versus							
	SDPSO	HCLDMS-PSO	TSLPSO	EPSO	GL-PSO	HCLPSO	OLPSO	LDWPSO
F1	+	+	+	+	+	+	+	+
F3	+	+	+	+	+	+	+	+
F4	+	+	+	+	+	+	+	+
F5	=	−	+	+	+	+	−	+
F6	+	=	−	=	−	−	−	+
F7	−	−	=	+	=	+	+	+
F8	=	−	+	+	+	+	=	+
F9	+	−	+	+	+	+	−	+
F10	=	−	=	−	−	−	−	+
F11	+	=	+	+	+	+	=	+
F12	+	+	+	+	+	+	+	+
F13	+	+	+	+	+	+	+	+
F14	+	+	+	+	+	+	+	+
F15	+	=	+	−	+	=	+	+
F16	=	−	+	=	+	+	+	+
F17	+	−	=	=	+	=	+	+
F18	+	+	+	+	+	+	+	+
F19	+	+	+	+	+	+	+	+
F20	=	−	=	=	+	=	+	+
F21	=	−	+	+	+	+	+	+
F22	=	−	=	−	=	−	−	+
F23	+	−	+	+	+	+	+	+
F24	+	−	+	+	+	+	+	+
F25	+	+	+	+	+	+	+	+
F26	+	−	+	−	+	=	=	+
F27	+	+	+	+	+	+	+	+
F28	+	+	+	+	+	+	+	+
F29	+	=	+	=	+	+	=	+
F30	+	+	+	+	+	+	+	+
+/=/−	21/7/1	12/4/13	23/5/1	20/5/4	25/2/2	24/4/3	20/4/5	29/0/0

**Table 12:** Ranks obtained through the Friedman test for 50D CEC2017 test suite problems.

Algorithm	SMLS-PSO	SDPSO	HCLDMS-PSO	TSLPSO	EPSO	GL-PSO	HCLPSO	OLPSO	LDWPSO
Average rank	**2.82**	5.13	3.48	5.81	4.61	5.48	4.94	5.38	7.37
Final rank	**1**	5	2	8	3	7	4	6	9
*p*-value	6.7239 × 10^−18^								

**Table 13:** Comparison of mean computation time(s) for CEC2017 test suite problems.

Algorithms	SMLS-PSO	SDPSO	HCLDMS-PSO	TSLPSO	EPSO	GL-PSO	HCLPSO	OLPSO	LDWPSO
30D problems	5.50	6.33	6.10	4.97	7.74	3.81	4.50	3.32	4.29
50D problems	12.75	15.46	16.94	11.52	21.05	10.22	11.18	8.84	11.48

**Table 14:** Comparison of the best fitness function value, the locatable space coverage rate, and average HDOP by rectangular layout, diamond layout, random layout, and 9 different PSO variants optimization layout models.

Algorithms	Rectangular Layout	Diamond Layout	Random Layout	SMLS-PSO	SDPSO	HCLDMS-PSO
Fitness	9.6336	1.1434	1.3113	**1.0900 ± 5.12** × **10^−05^**	1.1014 ± 4.70 × **10^−03^**	1.0998 ± 2.90 × **10^−03^**
Cover_rate	7.62%	89.12%	73.44%	**95.69% ± 8.68** × **10^−05^**	94.17% ± 6.60 × **10^−03^**	94.98% ± 4.10 × **10^−03^**
Average HDOP	1.5292	1.1934	1.1937	**1.1950 ± 9.87** × **10^−05^**	1.1934 ± 4.90 × **10^−03^**	1.2093 ± 9.70 × **10^−03^**
Rank				**1**	6	4
Wilcoxon rank sum test					+	+
Algorithms	**TSLPSO**	**EPSO**	**GL-PSO**	**HCLPSO**	**OLPSO**	**LDWPSO**
Fitness	1.0939 ± 2.90 × **10^−03^**	1.0963 ± 1.30 × **10^−03^**	1.1135 ± 9.90 × **10^−03^**	1.1005 ± 3.00 × **10^−03^**	1.1241 ± 1.32 × **10^−02^**	1.1590 ± 6.19 × **10^−02^**
Cover_rate	95.04% ± 5.60 × **10^−03^**	95.15% ± 1.60 × **10^−03^**	92.73% ± 1.16 × **10^−02^**	94.25% ± 5.00 × **10^−03^**	91.84% ± 1.43 × **10^−02^**	89.19% ± 6.17 × **10^−02^**
Average HDOP	1.1911 ± 5.00 × **10^−03^**	1.2021 ± 2.80 × **10^−03^**	1.1953 ± 1.12 × **10^−03^**	1.1925 ± 5.90 × **10^−03^**	1.2057 ± 9.30 × **10^−03^**	1.2367 ± 2.63 × **10^−02^**
Rank	2	3	7	5	8	9
Wilcoxon rank sum test	+	+	+	+	+	+

**Table 15:** Statistics of valid positioning epochs under various working conditions in dynamic trolley test with different layout modes.

Layout Mode	GNSS Epochs	Non-Occlusion		Metal Occlusion		Human Body Occlusion	
		Valid Epochs	Percentage	Valid Epochs	Percentage	Valid Epochs	Percentage
Rectangular layout	4926	2035	41.31%	1054	21.39%	1457	29.57%
SMLS-PSO optimized layout	5459	5135	94.06%	4441	81.35%	4974	91.11%

**Table 16:** Statistics of position errors for dynamic trolley test under different layout modes.

Layout Mode	Non-Occlusion		Metal Occlusion		Human Body Occlusion	
	Mean/m	RMS/m	Mean/m	RMS/m	Mean/m	RMS/m
Rectangular layout	0.660	0.704	0.976	1.085	0.799	0.892
SMLS-PSO optimized layout	0.659	0.725	1.018	1.134	0.897	0.998

## Data Availability

The datasets generated and/or analyzed during the current study are available from the corresponding author on reasonable request.

## Abbreviations

The following abbreviations are used in this manuscript:

PSO	Particle Swarm Optimization
BFGS	Broyden–Fletcher–Goldfarb–Shanno
CEC	Congress on Evolutionary Computation
*pbest*	personal history best position
*gbest*	global best position
CLPSO	Comprehensive learning PSO
EAs	Evolutionary algorithms
OLPSO	Orthogonal learning PSO
SMLS-PSO	Self-adaptive Multi-Learning Strategy PSO
SL-PSO	Social Learning PSO
GL-PSO	Genetic Learning PSO
DLPSO	Dimensional Learning Strategy PSO
HCLDMS-PSO	Heterogeneous comprehensive learning and dynamic multi-swarm PSO
DMS-PSO	Dynamic Multi-Swarm PSO
TSLPSO	Two-swarm learning PSO
LDWPSO	Linearly decreasing inertia weight PSO
GOPSO	Generalized opposition-based learning PSO
LP	Learning Period
HGCLPSO	Heterogeneous genetic learning and comprehensive learning strategy PSO
HCLPSO	Hybrid Collaborative Learning PSO
EPSO	Ensemble Particle Swarm Optimizer
UWB	Ultra-Wide Band
HDOP	Horizontal Dilution of Precision
TOA	Time-of-Arrival
TOF	Time-of-Flight
TWR	Two-Way-Ranging

## Appendix A

**Table A1:** Simple benchmark functions list.

Function Type	Function Name	Test Function	Search Range	$f_{\min}$
Unimodal and separable	F1: Step	$f=\sum_{i=1}^{n}{\left(x_{i}+0.5\right)}^{2}$	${\left[-5.12,5.12\right]}^{D}$	0
	F2: Sphere	$f=\sum_{i=1}^{n}{x_{i}}^{2}$	${\left[-100,100\right]}^{D}$	0
	F3: SumSquares	$f=\sum_{i=1}^{n}i\cdot {x_{i}}^{2}$	${\left[-10,10\right]}^{D}$	0
	F4: Quartic	$f=\sum_{i=1}^{n}i\cdot {x_{i}}^{4}$	${\left[-1.28,1.28\right]}^{D}$	0
Unimodal and non-separable	F5: Zakharov	$f=\sum_{i=1}^{n}x_{i}^{2}+(\sum_{i=1}^{n}0.5ix_{i}{)}^{2}+(\sum_{i=1}^{n}0.5ix_{i}{)}^{4}$	${\left[-5,10\right]}^{D}$	0
	F6: Schwefel 2.22	$f=\sum_{i=1}^{n}\left|x_{i}\right|+\prod_{i=1}^{n}\left|x_{i}\right|$	${\left[-10,10\right]}^{D}$	0
	F7: Schwefel 1.2	$f=\sum_{i=1}^{n}{\left(\sum_{j=1}^{i}x_{j}\right)}^{2}$	${\left[-100,100\right]}^{D}$	0
	F8: DixonPrice	$f={\left(x_{1}-1\right)}^{2}+\sum_{i=2}^{n}i\cdot {\left(2^{x_{i}^{2}}-x_{i-1}\right)}^{2}$	${\left[-10,10\right]}^{D}$	0
Multimodal and separable	F9: Rastrigin	$f=\sum_{i=1}^{n}\left[x_{i}^{2}-10\cos(2\pi x_{i})+10\right]$	${\left[-5.12,5.12\right]}^{D}$	0
Multimodal and non-separable	F10: Schaffer6	$f=\sum_{i=1}^{n-1}0.5+\frac{\sin^{2}(\sqrt{x_{i}^{2}+x_{i+1}^{2}})-0.5}{{\left(1+0.001(x_{i}^{2}+x_{i+1}^{2})\right)}^{2}}$	${\left[-100,100\right]}^{D}$	0
	F11: Rosenbrock	$f=\sum_{i=1}^{n-1}\left[100{\left(x_{i+1}-x_{i}^{2}\right)}^{2}+{\left(1-x_{i}\right)}^{2}\right]$	${\left[-30,30\right]}^{D}$	0
	F12: Griewank	$f=\frac{1}{4000}\sum_{i=1}^{n}x_{i}^{2}-\prod_{i=1}^{n}\cos(\frac{x_{i}}{\sqrt{i}})+1$	${\left[-600,600\right]}^{D}$	0
	F13: Ackley	$\begin{array}{l} f=-20\exp(-0.2\sqrt{\frac{1}{n}\sum_{i=1}^{n}x_{i}^{2}}-\\ \exp(\frac{1}{n}\sum_{i=1}^{n}\cos(2\pi x_{i}))+20+e \end{array}$	${\left[-32,32\right]}^{D}$	0
